# Genome-wide identification and functional analysis of lincRNAs acting as miRNA targets or decoys in maize

**DOI:** 10.1186/s12864-015-2024-0

**Published:** 2015-10-15

**Authors:** Chunyan Fan, Zhiqiang Hao, Jiahong Yan, Guanglin Li

**Affiliations:** College of Life Science, Shaanxi Normal University, Xi’an, 710119 China

**Keywords:** Maize, miRNAs, lincRNAs, Targets, Decoys

## Abstract

**Background:**

Long intergenic noncoding RNAs (lincRNAs) are endogenous non-coding RNAs (ncRNAs) that are transcribed from ‘intergenic’ regions of the genome and may play critical roles in regulating gene expression through multiple RNA-mediated mechanisms. MicroRNAs (miRNAs) are single-stranded small ncRNAs of approximately 21–24 nucleotide (nt) that are involved in transcriptional and post-transcriptional gene regulation. While miRNAs functioning as mRNA repressors have been studied in detail, the influence of miRNAs on lincRNAs has seldom been investigated in plants.

**Methods:**

LincRNAs as miRNA targets or decoys were predicted via GSTAr.pl script with a set of rules, and lincRNAs as miRNA targets were validated by degradome data. Conservation analysis of lincRNAs as miRNA targets or decoys were conducted using BLASTN and MAFFT. The function of lincRNAs as miRNA targets were predicted via a lincRNA-mRNA co-expression network, and the function of lincRNAs as miRNA decoys were predicted according to the competing endogenous RNA (ceRNA) hypothesis.

**Results:**

In this work, we developed a computational method and systematically predicted 466 lincRNAs as 165 miRNA targets and 86 lincRNAs as 58 miRNA decoys in maize *(Zea mays L*.). Furthermore, 34 lincRNAs predicted as 33 miRNA targets were validated based on degradome data. We found that lincRNAs acting as miRNA targets or decoys are a common phenomenon, which indicates that the regulated networks of miRNAs also involve lincRNAs. To elucidate the function of lincRNAs, we reconstructed a miRNA-regulated network involving 78 miRNAs, 117 lincRNAs and 8834 mRNAs. Based on the lincRNA-mRNA co-expression network and the competing endogenous RNA hypothesis, we predicted that 34 lincRNAs that function as miRNA targets and 86 lincRNAs that function as miRNA decoys participate in cellular and metabolic processes, and play role in catalytic activity and molecular binding functions.

**Conclusions:**

This work provides a comprehensive view of miRNA-regulated networks and indicates that lincRNAs can participate in a layer of regulatory interactions as miRNA targets or decoys in plants, which will enable in-depth functional analysis of lincRNAs.

**Electronic supplementary material:**

The online version of this article (doi:10.1186/s12864-015-2024-0) contains supplementary material, which is available to authorized users.

## Background

Long noncoding RNAs (lncRNAs) are generally long transcripts of more than 200 nucleotide (nt) that lack a coding sequence (CDS) or open reading frame (ORF) [1, 2]. Despite exhibiting lower expression levels compared with mRNAs, lncRNAs can regulate gene expression at the transcriptional and post-transcriptional levels [3–7]. As one of the largest classes of lncRNAs, long intergenic noncoding RNAs (lincRNAs) are endogenous lncRNAs that are transcribed from ‘intergenic’ regions of the genome. They play critical roles in regulating multiple important biological processes in humans and other animals, including cell cycle regulation, immune surveillance and embryonic stem cell differentiation [8–14], while they primarily participate in the environmental stimulus response, vernalization and nodulation in plants, including *Arabidopsis thaliana* [15], *Triticum aestivum* [16], *Cucumis sativus* [17], *Setaria italic* [18], *Populus trichocarpa* [19] and *Zea mays* [20–22]. However, compared with animal lincRNAs, the functions of plant lincRNAs and their regulatory roles remain largely undiscovered.

Unlike lincRNAs, plant microRNAs (miRNAs) are approximately 21–24 nucleotide (nt) single-stranded, small non-coding RNAs that typically form near-perfect duplexes with their targets and mediate cleavage or translation repression at the post-transcriptional level [23, 24]. They play vital roles in regulating a broad range of biological metabolic processes, including roles in plant development, flowering time, leaf morphogenesis, hormone signaling and responses to environmental stresses, such as phosphate or/and sulfate stress [25–30]. miRNAs usually regulate the expression of their mRNA targets through cleavage in plants [31, 32]. However, recent studies suggest that miRNAs function in a more sophisticated way than was initially assumed. In addition to protein-coding RNAs acting as miRNA targets, lincRNAs can also be directly targeted by miRNAs for cleavage [19, 33–35].

More interestingly, lincRNAs can also serve as miRNA decoys, miRNA sponges, target mimicry, or target mimics to interfere with the miRNA-mediated regulation of their mRNA targets. Similar to the sequence-dependent interactions of miRNAs with their mRNA targets, miRNA decoys also rely on the sequence-dependent interaction of miRNAs with lincRNAs, except for the bulges in the middle of miRNA-lincRNA duplexes. If lincRNAs acting as miRNA decoys and mRNAs acting as miRNA targets can be bound by the same miRNAs, then lincRNAs could function as competing endogenous RNAs (ceRNAs); they could directly interact with the specific miRNA and sequester it in a type of target mimicry to protect target mRNAs from repression, which is known as the “ceRNA hypothesis” [36, 37]. In animals, the long noncoding RNA linc-MD1 can act as a miR133 and miR135 sponge and up-regulate muscle-specific expression of the respective miR133 and miR135 targets MAML1 and MEF2C [38]. In plants, the classical example of an miRNA decoy is *IPS1*, which is a long non-coding RNA that contains an ath-miR399 decoy site and can serve as an miRNA decoy to inactivate ath-miR399 and up-regulate the expression of the ath-miR399 primary target *PHO2* [39]. In rice, it has been reported that two lincRNAs that act as decoys of miR160 and miR164 can regulate floral and/or seed development [40]. Recently, 25 miRNA decoys from *Arabidopsis* and 94 miRNA decoys from rice were identified; overexpressing the decoys of miR160 and miRNA166 can alter plant development, indicating that ncRNAs, short ORF encoding genes and intergenic sequences acting as miRNA decoys are functional in plants [41].

Maize (*Zea mays L.*) is one of the most important crops worldwide. It serves as a food source for people around the world and as a model organism in genetics research [42]. With the release of the maize genome, increasing amounts of transcriptome data; degradome data; and specific data on miRNAs, lincRNAs and mRNAs have been accumulated. It is now possible for us to investigate the function of lincRNAs as miRNA targets or decoys in maize. Here, lincRNAs acting as miRNA targets were initially identified based on degradome data, and lincRNAs that may act as miRNA decoys were subsequently predicted. To explore the function of lincRNAs acting as miRNA targets or decoys, a genome-scale network among miRNAs, lincRNAs acting as miRNA targets, lincRNAs acting as miRNA decoys, and mRNAs was first constructed. Then, the functions of lincRNAs acting as miRNA targets were predicted and annotated via a co-expression network between lincRNAs and mRNAs, and the functions of lincRNAs acting as miRNA decoys were predicted and annotated according to the ceRNA hypothesis. Our research demonstrates that lincRNAs can act as miRNA targets or decoys to mediate the regulation of gene expression, and the annotation of lincRNA functions will facilitate the validation of the lincRNA functions in the future.

## Methods

### LincRNA and cDNA data

Primary data on lincRNAs were first integrated from three published studies on maize, consisting of 1704 high-confidence lncRNAs, 439 lincRNAs, and 664 putative maize lncRNAs [20–22]. Then, lncRNAs that were not located in intergenic regions and lincRNAs that were small RNA precursors were filtered out, and a total of 1831 lincRNAs were obtained and used in further analyses (Additional file 1). To distinguish the lincRNAs from these three data sources, the first authors’ names were added to the IDs of the lincRNAs. Maize cDNA data were downloaded from MaizeGDB ftp://ftp.ensemblgenomes.org/pub/plants/release-22/fasta/zea_mays/.)

### miRNA data

Data on mature miRNAs were downloaded from miRBase (version 21: June 2014, http://www.mirbase.org/) [43, 44], and 321 maize miRNAs were extracted. A total of 203 unique miRNAs were obtained after merging these sequences with different miRNA IDs.

### Degradome data

The degradome data from maize were downloaded from NCBI’s Gene Expression Omnibus (GEO) with the accession numbers of SRX222260, SRX222262, SRX222264, SRX222266 (http://www.ncbi.nlm.nih.gov/sra/?term=SRP018376) [45–47]. The raw reads from the above data were first processed using the FASTAX-Toolkit to trim adapter sequences with many “N” and ignored reads that were less than 18 nt. Then, the redundant reads were merged and 3268059, 4106567, 2682186 and 2965163 unique reads were obtained from the optional nitrate root tip, low nitrate root tip, low nitrate leaf and optional nitrate leaf, respectively (Additional file 2).

### Prediction of miRNA targets

The miRNA targets of lincRNAs or cDNAs were predicted using GSTAr.pl script, and the minimum free energy (MFE) of miRNA-lincRNA or miRNA-cDNA duplexes was calculated with the RNAhybrid program [48–50]. Then, a modified version of the CleaveLand4 program was used to identify the potential cleavage sites of miRNAs in the corresponding targets based on degradome data http://sites.psu.edu/axtell/software/cleaveland4/) [51]. To obtain high-quality lincRNAs acting as miRNA targets and to distinguish those lincRNAs acting as miRNA decoys, the following rules were used: at most, one mismatch or indel was allowed between the 9^th^ and 12^th^ positions of the 5′ end of miRNA sequences, the total number of bulges or mismatches in the other regions was not allowed to exceed 4 nt, and no continuous mismatches were allowed [41, 51]. In addition, target plots indicating the abundance of each distinct read for the lincRNAs acting as miRNA targets were generated.

### Prediction of miRNA decoys

LincRNAs potentially acting as miRNA decoys were predicted based on Wu’s methods with a slight modification [41, 52]. Generally, the following set of rules was used: (1) the number of mismatches or indels should be larger than 1 and less than 6 between the 9^th^ and 12^th^ positions of the 5′ end of the miRNA sequences; (2) perfect nucleotide pairing was required between the 2^nd^ and 8^th^ positions of the 5′ end of miRNA sequences; and (3) the number of mismatches and indels should be no more than 4 in other regions. These rules were implemented using in-house Perl scripts.

### Conservation analysis of lincRNAs acting as miRNA targets or decoys

To investigate the conservation of lincRNAs acting as miRNA targets or decoys, five genomes of other monocotyledons (monocots) (*Sorghum bicolor, Setaria italica, Panicum virgatum, Oryza sativa* and *Brachypodium distachyon*) were downloaded from Phytozome (v9.1) (http://www.phytozome.net/) [53], and the lincRNA regions that paired with miRNA targets or decoys were searched against the 5 monocot genomes using BLASTN with a cutoff threshold of an E-value less than 1e-1 [54]. Then, the significantly matched regions plus their flanking regions (100 bp in total) were obtained [55]. Finally, multiple sequence alignment was conducted with MAFFT v6.864b, using parameter settings of maxiterate 1000 and localpair [56]. If the identities between the conserved sites were greater than 80%, then the conserved sites were highlighted.

### Construction of miRNA-lincRNA-mRNA networks

To infer the function of lincRNAs, networks were constructed based on the complementary pairs between miRNAs and lincRNAs and between miRNAs and mRNAs. The nodes in the networks consisted of miRNAs, lincRNAs acting as miRNA targets, lincRNAs acting as miRNA decoys, mRNAs acting as miRNA targets, and mRNAs acting as miRNA decoys. The miRNA-lincRNA-mRNA networks were visualized with Cytoscape 3.1.1 [57].

### Functional prediction of lincRNAs acting as miRNA targets based on the lincRNA-mRNA co-expression networks

Fifty-four datasets, including 30 RNA-seq experiments performed in 13 different tissues (leaf, immature ear, immature tassel, seed, endosperm, embryo, embryo sac, anther, ovule, pollen, silk, root and shoot apical tissues), were applied to construct a co-expression network between lincRNAs acting as miRNA targets and mRNA genes [58–63]. The construction method was similar to that of Liao [64] and Hao [17]. In general, the pipeline for constructing the co-expression network was as follows: (1) genes, including mRNAs and lincRNAs, whose variances ranked in the top 75 % of the expression profiles were retained; (2) the *p*-value of Pearson’s correlation coefficient (*Pcc*) was calculated for each pair of genes using Fisher’s asymptotic test in the *WGCNA* library of R [65], and these values were adjusted using the Bonferroni correction method; and (3) co-expression relationships showing adjusted *p*-values of less than 0.05 and ranking in the top 5 % and bottom 5 % of *Pcc* were selected for further analysis. The Bonferroni multiples test was executed using the *multtest* package from R. The co-expression networks were also visualized using Cytoscape [57].

Based on the co-expression network between lincRNAs acting as miRNA targets and mRNAs, we used the AgriGO toolkit and input the list of mRNA genes to predict the function of these lincRNAs [66].

### Functional prediction of lincRNAs acting as miRNA decoys based on miRNA-lincRNA-mRNA networks

Based on the ceRNA hypothesis and gene ontology (GO) analysis, the function of lincRNAs acting as miRNA decoys can be speculated based on the miRNA-lincRNA-mRNA networks. AgriGO, an integrated web-based GO analysis toolkit, was employed for the functional annotation and enrichment analysis [66]. The IDs of all of the listed mRNAs connected with lincRNAs acting as miRNA decoys were submitted for GO analysis, and the overrepresented GO terms in the “biological process”, “cellular component” and “molecular function” categories were obtained using Fisher’s exact test and the Bonferroni multiples test (*P*-value < 0.05).

## Results

### Identification of lincRNAs as putative miRNA targets

Previous research has suggested that miRNAs play roles in regulating the expression of mRNAs, but the comprehensive patterns of miRNA regulation of lincRNAs remain unknown. To systematically investigate the miRNA-mediated regulatory mechanism of lincRNAs, a method for predicting miRNA targets among lincRNAs was applied (see Materials and Methods). The results revealed 789 miRNA-lincRNA interactions (Additional file 3). In total, 466 lincRNA targets were predicted for 165 miRNAs in *Zea mays*.

**Fig. 1:**
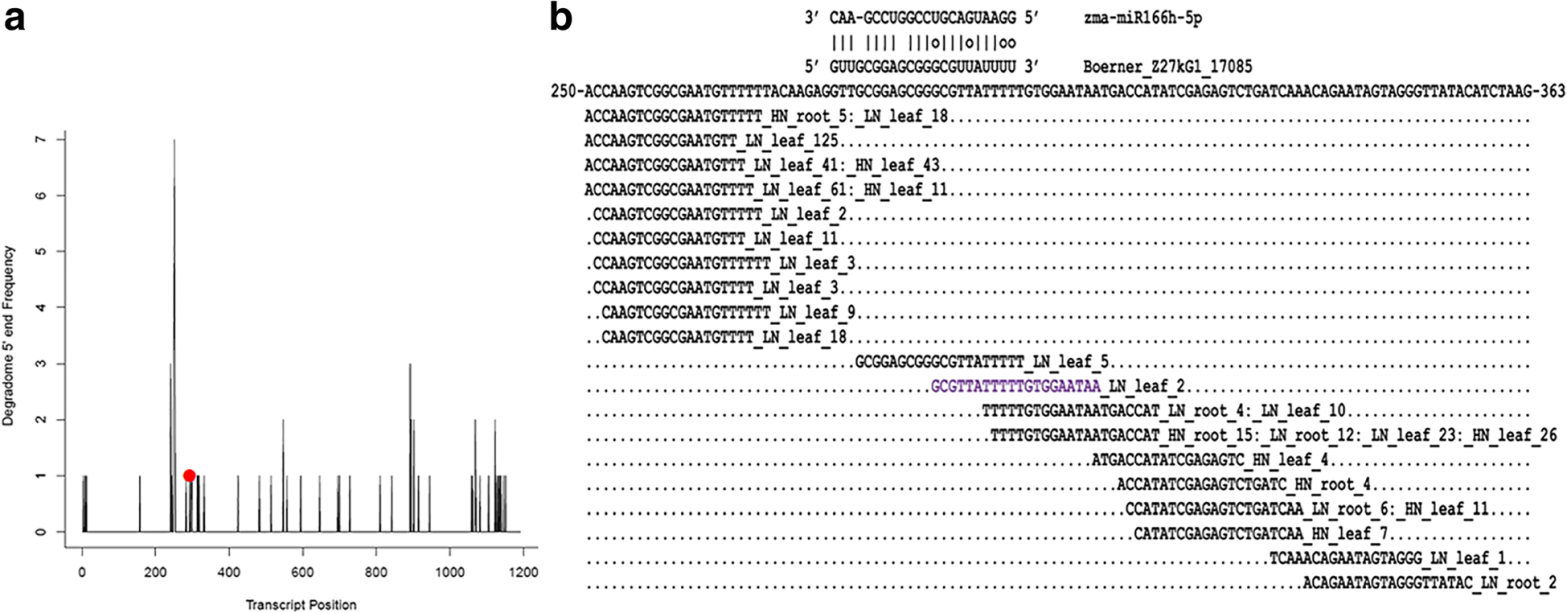
Target plots (t-plots) of the confirmed zma-miR166h-5p target and the distribution of degradome reads among lincRNAs. **a** Cleavage characteristics of Boerner_Z27kG1_17085, which functions as a zma-miR166h-5p target. The abundance of each sequenced read is plotted as a function of the position of its 5′ end in the transcripts. The peaks of the signatures at the validated cleavage sites of the corresponding miRNAs are shown in red (dots). **b** miRNA:lincRNA alignment: the reads of the degradome with 5′ ends at the indicated positions are shown in black, while the reads at position 9, 10, 11, or 12 of the inset miRNA target alignment are shown in purple (the cleavage site was counted from the 5′ end of the purple reads, which was position 10 in this example)

To eliminate potential false-positive lincRNAs predicted as miRNA targets, we applied degradome reads to validate miRNA targets using a modified version of the CleaveLand pipeline [51]. The results showed that 42 miRNA-lincRNA duplexes were supported by the degradome reads, which were formed by 33 miRNAs and 34 lincRNAs (Table 1, Additional file 4). When the degradome reads were mapped on each lincRNA, the abundance of the degradome reads at each position of the lincRNAs and the cleaved positions in each lincRNA could be obtained. For example, the abundance of degradome reads and cleavage sites in the lincRNA Boerner_Z27kG1_17085, which can act as a target of zma-miR166h-5p, is shown in Fig. 1.

**Table 1:** LincRNAs acting as miRNA targets validated using degradome data

zma-miRNAs^a^	Transcripts^b^	start-end^c^	MFEratio^d^	Category^e^	*P*-value^f^	Degradome data file^g^
zma-miR156e-3p	Li_TCONS_00080887	75-95	0.674311926605505	4	0.071329710792311	LN_root
zma-miR156e-3p	zhang_TCONS_00012690	489-511	0.768348623853211	4	0.020921336970078	LN_root
zma-miR156h-3p	zhang_TCONS_00012690	625-646	0.713936430317848	4/4/4	0.0514855197295749/0.046703544571294/0.0376430084254001	LN_root/LN_leaf/HN_root
zma-miR159d-3p:zma-miR159c-3p	Boerner_Z27kG1_14953	633-654	0.723192019950125	4	0.0382439063811821	HN_leaf
zma-miR159e-5p	Boerner_Z27kG1_09751	293-312	0.686602870813397	4/4	0.046703544571294/0.0376430084254001	LN_leaf/HN_root
zma-miR159e-5p	Boerner_Z27kG1_15115	817-837	0.657894736842105	4	0.0750252163870715	HN_leaf
zma-miR160b-3p:zma-miR160g-3p	Boerner_Z27kG1_08283	74-94	0.691588785046729	4	0.0302295095199341	HN_root
zma-miR160c-3p	Boerner_Z27kG1_16361	1423-1444	0.689156626506024	4	0.0824909413024753	LN_leaf
zma-miR160c-3p	Boerner_Z27kG1_23317	2302-2323	0.667469879518072	4/2/4	0.155614651330728/0.0297578023818319/0.117310766689232	LN_root/LN_leaf/HN_leaf
zma-miR162-5p	Boerner_Z27kG1_13892	28-48	0.659605911330049	4/4	0.155614651330728/0.115544761245953	LN_root/HN_root
zma-miR164b-3p	Boerner_Z27kG1_01046	97-117	0.67175572519084	1/0/0/0	0.0137287981463647/0.00691182420446057/0.00821020675558204/0.00827523084432658	LN_root/LN_leaf/HN_root/HN_leaf
zma-miR164d-3p	Boerner_Z27kG1_22106	573-593	0.693506493506494	4	0.181974073806857	LN_root
zma-miR164e-3p	Boerner_Z27kG1_03819	109-130	0.660831509846827	4	0.141916540983859	LN_leaf
zma-miR166h-5p	Boerner_Z27kG1_17085	280-301	0.688836104513064	4	0.0282897431510059	LN_leaf
zma-miR166i-5p	Boerner_Z27kG1_06707	85-104	0.757009345794392	4	0.0189498889103007	LN_leaf
zma-miR166i-5p	Boerner_Z27kG1_17308	553-572	0.77803738317757	4/2/4/3	0.0105159612050723/0.00188631556542906/0.00764459114629912/0.00047103572965379	LN_root/LN_leaf/HN_root/HN_leaf
zma-miR166n-5p	Boerner_Z27kG1_01291	759-780	0.65281173594132	4	0.262254475002389	HN_leaf
zma-miR169c-3p	Boerner_Z27kG1_22188	252-273	0.686635944700461	4	0.0312172902072148	LN_root
zma-miR169f-3p	Boerner_Z27kG1_15675	450-471	0.696517412935323	0	0.00163062708112194	LN_leaf
zma-miR169i-3p:zma-miR169j-3p:zma-miR169k-3p	Boerner_Z27kG1_23086	819-837	0.665753424657534	4	0.457851673892433	LN_leaf
zma-miR169l-3p	Boerner_Z27kG1_06005	556-575	0.661498708010336	4	0.0736720564421364	LN_leaf
zma-miR169m-3p	zhang_TCONS_00011169	341-360	0.660220994475138	2/3/3	0.04068724565891/0.0034421015727073/0.0103116935935227	LN_leaf/HN_root/HN_leaf
zma-miR171b-5p	Boerner_Z27kG1_16154	453-472	0.654285714285714	4	0.128405843607718	LN_root
zma-miR2118d	Boerner_Z27kG1_20838	4-26	0.660674157303371	4	0.0907587348469249	LN_root
zma-miR2275a-3p	Li_TCONS_00089775	205-227	0.669724770642202	4	0.108447036911749	LN_leaf
zma-miR394b-3p:zma-miR394a-3p	Boerner_Z27kG1_16154	1242-1260	0.691516709511568	4/2/4/4	0.0414049715995405/0.00752393997803091/0.0302295095199341/0.0307139613443539	LN_root/LN_leaf/HN_root/HN_leaf
zma-miR395o-3p	Boerner_Z27kG1_21671	386-406	0.67479674796748	4	0.00764459114629912	HN_root
zma-miR399e-5p	Boerner_Z27kG1_03819	175-196	0.652173913043478	4/4/4/2	0.301930016223076/0.277645717435721/0.22965334757042/0.0560077959605969	LN_root/LN_leaf/HN_root/HN_leaf
zma-miR399e-5p	Boerner_Z27kG1_15755	72-93	0.678743961352657	4	0.166191501641553	LN_leaf
zma-miR399e-5p	Boerner_Z27kG1_22850	800-821	0.70048309178744	4	0.0457153546791773	HN_leaf
zma-miR408b-3p:zma-miR408a	Boerner_Z27kG1_01046	94-115	0.655097613882863	2/0/2/0	0.0743714637805463/0.00893560703937002/0.0431631594963204/0.0106960643831452	LN_root/LN_leaf/HN_root/HN_leaf
zma-miR408b-5p	zhang_TCONS_00011169	185-204	0.675438596491228	4/4/3	0.108447036911749/0.0879746832508639/0.0056378079964241	LN_leaf/HN_root/HN_leaf
zma-miR444a:zma-miR444b	Boerner_Z27kG1_20838	5-25	0.728291316526611	4/0	0.0414049715995405/0.000941738699572037	LN_root/HN_root
zma-miR482-3p	Boerner_Z27kG1_08283	600-621	0.696022727272727	2	0.0781478318804442	HN_leaf
zma-miR482-3p	Boerner_Z27kG1_22204	94-112	0.735795454545454	4/4	0.207510626541854/0.155345100314279	LN_root/HN_root
zma-miR482-3p	Li_TCONS_00030374	285-304	0.732954545454545	4	0.170699850059004	HN_leaf
zma-miR482-3p	Li_TCONS_00055761	33-52	0.784090909090909	4	0.0531287606730582	HN_leaf
zma-miR528a-3p:zma-miR528b-3p	Boerner_Z27kG1_01046	97-118	0.68957345971564	1/0/0/0	0.0109982003430358/0.00610115440783998/0.00657357579419915/0.00730523957678519	LN_root/LN_leaf/HN_root/HN_leaf
zma-miR528a-3p:zma-miR528b-3p	Boerner_Z27kG1_08632	462-482	0.665876777251185	4	0.368800900766741	HN_leaf
zma-miR528a-3p:zma-miR528b-3p	Boerner_Z27kG1_23730	573-593	0.665876777251185	4	0.348799968161408	HN_leaf
zma-miR529-3p	Boerner_Z27kG1_17308	93-112	0.650872817955112	3	0.025580206396939	HN_leaf
zma-miR529-5p	zhang_TCONS_00077805	55-76	0.723785166240409	4	0.0312172902072148	LN_root
zma-miR827-5p	Boerner_Z27kG1_13480	1365-1385	0.654434250764526	4	0.249470323366813	LN_leaf

^a^miRNA data from miRBase 21.0. ^b^Targeted lincRNA genes for the miRNAs. ^c^The starting and terminating sites on the lincRNAs when it is bound by miRNAs. ^d^MFEsite/MFEperfect, the calculation based on the method in Tafer et al.[50]. ^e^Classification of the splicing signal of the alignments; the classifications corresponded to the degradome data files. ^f^ *p*-value for the degradome reads in different degradome data files. ^g^The evidence file for the alignments

### Conservation of lincRNAs as miRNA targets between six monocotyledons

**Fig. 2:**
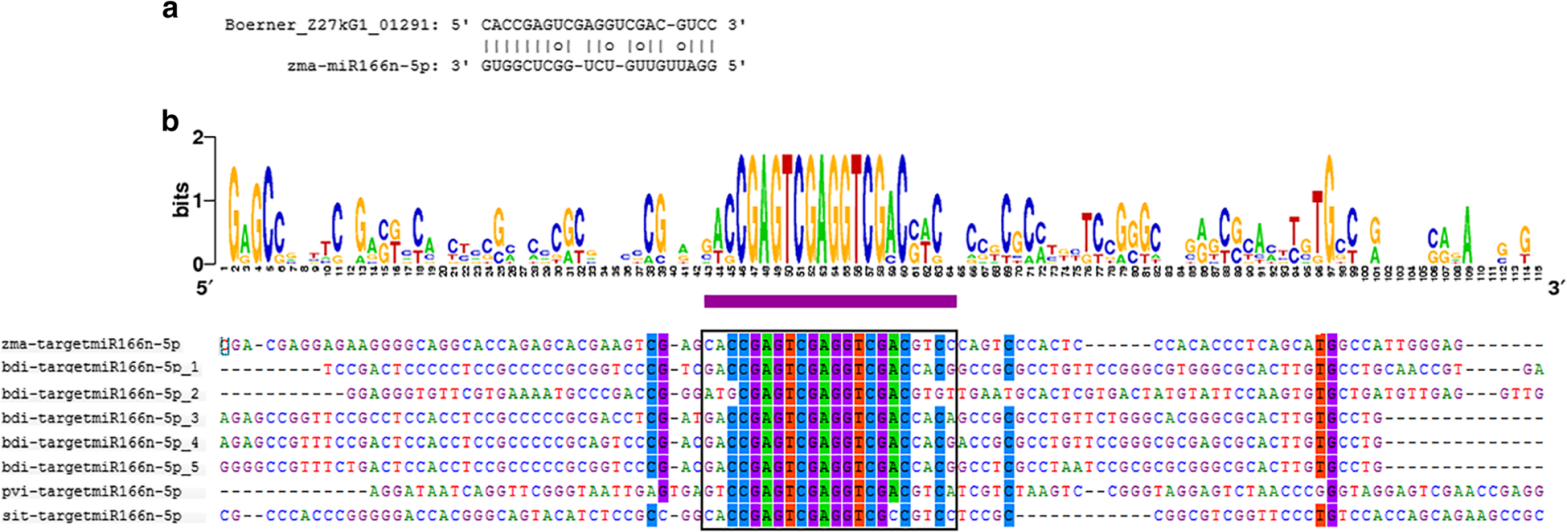
zma-miR166n-5p target sites in lincRNAs in maize. **a** The predicted alignment between zma-miR166n-5p and its target in lincRNA. **b** Sequence alignments of lincRNA targets and the surrounding regions for zma-miR166n-5p in maize and 3 other species. The target sites pairing with zma-miR166n-5p are underlined with black boxes. The conservation status of the sequences was analyzed and presented using Weblogo. The logo consists of stacks of symbols, with one stack for each position in the sequence. The overall height of the stack indicates the sequence conservation at that position, while the height of symbols within the stacks indicates the relative frequency of nucleic acids at that position (bdi: B. distachyon; pvi: P. virgatum; sit: S. italica)

To investigate the conservation of lincRNAs as miRNA targets, the lincRNA regions that paired with miRNAs were searched against the 5 genomes of monocots, and the significant matched regions plus their flanking regions were obtained. Conservation analysis was performed, and 12 of 33 miRNAs were found to show conserved target regions in lincRNAs among maize and three to five other species. For example, the sequence logo and multiple sequence alignment of zma-miR166n-5p targets in lincRNAs provide a precise description of the conservation of these target regions (Fig. 2). However, the lincRNA regions outside of the predicted miRNA binding sites were not conserved, except for the lincRNAs targeted by zma-miR160b/g-3p and zma-miR169l-3p, which were conserved among 4 and 5 species, respectively (Additional file 5). In summary, lincRNAs acting as miRNA targets are a common phenomenon among monocots.

### Identification of lincRNAs acting as miRNA decoys

Previous studies have shown that the duplexes formed by miRNAs and miRNA decoys usually contain bulges or mismatches in the middle of the miRNA binding sites, which is thought to block the interaction between miRNAs and their specific mRNA targets [41, 52]. GSTAr.pl can efficiently identify sites with large bulges in the alignments between miRNAs and lincRNAs. Therefore, we used a computational pipeline to identify lincRNAs acting as miRNA decoys in maize. In total, we found that 86 lincRNAs that may act as miRNA decoys could be bound by 58 miRNAs and formed 104 miRNA-lincRNA duplexes (Table 2, Additional file 6).

**Table 2:** LincRNAs acting as miRNA decoys

zma-miRNAs^a^	Transcript ^b^	start-end^c^	MFEperfect^d^	MFEsite^e^	MFEratio^f^
zma-miR156a-3p	Li_TCONS_00044513	70-90	−41.7	−27.3	0.654676258992806
zma-miR156b-3p	Li_TCONS_00088709	477-501	−40.8	−28.28	0.693137254901961
zma-miR159a-5p	Li_TCONS_00096446	109-129	−36.4	−23.8	0.653846153846154
zma-miR159b-5p:zma-miR159k-5p:zma-miR159j-5p	Boerner_Z27kG1_11801	143-162	−35.3	−23.7	0.671388101983003
zma-miR159b-5p:zma-miR159k-5p:zma-miR159j-5p	Boerner_Z27kG1_16385	143-162	−35.3	−23.7	0.671388101983003
zma-miR159b-5p:zma-miR159k-5p:zma-miR159j-5p	Li_TCONS_00012571	604-627	−35.3	−23.4	0.662889518413598
zma-miR159d-3p:zma-miR159c-3p	zhang_TCONS_00029225	1271-1291	−40.1	−27.3	0.680798004987531
zma-miR159e-3p	Boerner_Z27kG1_01522	880-899	−38.2	−25.2	0.659685863874345
zma-miR159e-3p	Boerner_Z27kG1_22626	192-211	−38.2	−25.3	0.662303664921466
zma-miR159e-3p	Li_TCONS_00012087	121-141	−38.2	−26.9	0.704188481675393
zma-miR159e-3p	zhang_TCONS_00056321	208-228	−38.2	−28.48	0.745549738219895
zma-miR159e-5p	Li_TCONS_00071752	34-50	−41.8	−27.5	0.657894736842105
zma-miR159g-3p	Boerner_Z27kG1_05478	507-526	−37.6	−25.3	0.672872340425532
zma-miR159g-3p	Boerner_Z27kG1_09702	320-340	−37.6	−27.7	0.736702127659574
zma-miR159g-5p:zma-miR159h-5p:zma-miR159i-5p	Boerner_Z27kG1_11801	143-162	−37.8	−27.2	0.719576719576720
zma-miR159g-5p:zma-miR159h-5p:zma-miR159i-5p	Boerner_Z27kG1_16385	143-162	−37.8	−27.2	0.719576719576720
zma-miR159g-5p:zma-miR159h-5p:zma-miR159i-5p	Li_TCONS_00012571	604-627	−37.8	−27.3	0.722222222222222
zma-miR159g-5p:zma-miR159h-5p:zma-miR159i-5p	Li_TCONS_00059484	191-211	−37.8	−24.88	0.658201058201058
zma-miR160c-3p	Boerner_Z27kG1_01735	212-233	−41.5	−27.4	0.660240963855422
zma-miR160d-3p	Boerner_Z27kG1_01291	1077-1102	−44.4	−30.1	0.677927927927928
zma-miR160f-3p	Li_TCONS_00062998	301-321	−46.8	−31	0.662393162393162
zma-miR162-5p	Li_TCONS_00020299	306-326	−40.6	−27.1	0.667487684729064
zma-miR164b-3p	Boerner_Z27kG1_17564	219-235	−39.3	−27.3	0.694656488549618
zma-miR164b-3p	Li_TCONS_00023489	142-160	−39.3	−27.5	0.699745547073791
zma-miR164b-3p	Li_TCONS_00023490	142-160	−39.3	−27.5	0.699745547073791
zma-miR164b-3p	Li_TCONS_00052687	45-68	−39.3	−32.1	0.816793893129771
zma-miR164b-3p	Li_TCONS_00062623	8-31	−39.3	−30.08	0.765394402035623
zma-miR164c-3p:zma-miR164h-3p	Li_TCONS_00048247	268-291	−39.7	−26.1	0.657430730478589
zma-miR164c-5p:zma-miR164a-5p:zma-miR164b-5p:zma-miR164g-5p:zma-miR164d-5p	Li_TCONS_00011715	349-371	−44.3	−32.8	0.740406320541761
zma-miR164c-5p:zma-miR164a-5p:zma-miR164b-5p:zma-miR164g-5p:zma-miR164d-5p	Li_TCONS_00031357	1-20	−44.3	−30.9	0.697516930022573
zma-miR164f-5p	Li_TCONS_00011715	349-371	−44.3	−34.1	0.769751693002257
zma-miR164f-5p	Li_TCONS_00031357	1-20	−44.3	−30.3	0.683972911963883
zma-miR164h-5p	Li_TCONS_00011715	349-371	−43.6	−31.1	0.713302752293578
zma-miR164h-5p	Li_TCONS_00031357	1-20	−43.6	−31.1	0.713302752293578
zma-miR166g-5p	Li_TCONS_00014574	19-43	−37.9	−25.6	0.675461741424802
zma-miR166n-5p	Boerner_Z27kG1_07658	926-945	−40.9	−28.9	0.706601466992665
zma-miR166n-5p	Boerner_Z27kG1_17312	433-455	−40.9	−29.2	0.713936430317848
zma-miR167e-3p	zhang_TCONS_00077767	493-515	−38.6	−25.5	0.660621761658031
zma-miR167f-3p	Boerner_Z27kG1_02792	196-221	−43.5	−28.3	0.650574712643678
zma-miR167j-3p	Li_TCONS_00096821	264-283	−32.4	−21.48	0.662962962962963
zma-miR169i-3p:zma-miR169j-3p:zma-miR169k-3p	Boerner_Z27kG1_15115	783-800	−36.5	−26.1	0.715068493150685
zma-miR169i-3p:zma-miR169j-3p:zma-miR169k-3p	Li_TCONS_00032815	227-244	−36.5	−25	0.684931506849315
zma-miR169i-3p:zma-miR169j-3p:zma-miR169k-3p	Li_TCONS_00091165	524-540	−36.5	−24.2	0.663013698630137
zma-miR169l-5p	Li_TCONS_00023317	7-30	−40.2	−27.04	0.672636815920398
zma-miR169m-3p	Li_TCONS_00034371	236-259	−36.2	−24.8	0.685082872928177
zma-miR169n-3p	Li_TCONS_00064018	104-127	−40.4	−26.88	0.665346534653465
zma-miR169n-3p	Li_TCONS_00064018	129-152	−40.4	−26.88	0.665346534653465
zma-miR169n-3p	Li_TCONS_00096947	82-105	−40.4	−26.88	0.665346534653465
zma-miR169o-3p	Boerner_Z27kG1_03458	184-205	−40.8	−29.1	0.713235294117647
zma-miR169q-3p	Boerner_Z27kG1_03458	189-205	−39.5	−26.5	0.670886075949367
zma-miR169q-3p	Li_TCONS_00041379	300-317	−39.5	−26.3	0.665822784810127
zma-miR169q-3p	Li_TCONS_00064018	104-127	−39.5	−27.4	0.693670886075949
zma-miR169q-3p	Li_TCONS_00064018	129-152	−39.5	−27.4	0.693670886075949
zma-miR169q-3p	Li_TCONS_00096947	82-105	−39.5	−27.4	0.693670886075949
zma-miR169q-3p	zhang_TCONS_00028666	216-234	−39.5	−26.9	0.681012658227848
zma-miR169q-3p	zhang_TCONS_00056448	36-59	−39.5	−25.7	0.650632911392405
zma-miR171a-5p	Boerner_Z27kG1_20123	1069-1091	−35.8	−23.4	0.653631284916201
zma-miR171f-5p	Boerner_Z27kG1_04122	35-55	−38.8	−25.4	0.654639175257732
zma-miR171f-5p	Li_TCONS_00027786	158-178	−38.8	−26.1	0.672680412371134
zma-miR171f-5p	Li_TCONS_00096642	338-357	−38.8	−25.7	0.662371134020619
zma-miR171h-3p:zma-miR171k-3p	Boerner_Z27kG1_10860	672-691	−37.4	−24.7	0.660427807486631
zma-miR171h-3p:zma-miR171k-3p	Li_TCONS_00082779	252-270	−37.4	−24.8	0.663101604278075
zma-miR171k-5p:zma-miR171h-5p	Boerner_Z27kG1_00580	730-750	−35.4	−23.6	0.666666666666667
zma-miR171n-5p	Boerner_Z27kG1_20123	1069-1091	−35.8	−25.9	0.723463687150838
zma-miR172a:zma-miR172c-3p:zma-miR172d-3p:zma-miR172b-3p	Li_TCONS_00065651	597-618	−33.2	−23.6	0.710843373493976
zma-miR172b-5p:zma-miR172d-5p	Li_TCONS_00044327	23-44	−34.5	−22.7	0.657971014492754
zma-miR172c-5p	Li_TCONS_00044327	23-44	−36.5	−25.3	0.693150684931507
zma-miR172e	Li_TCONS_00065651	596-618	−36	−23.5	0.652777777777778
zma-miR319a-5p:zma-miR319c-5p	Li_TCONS_00081252	74-96	−39.1	−26.42	0.675703324808184
zma-miR319a-5p:zma-miR319c-5p	zhang_TCONS_00063429	16-39	−39.1	−27.16	0.694629156010230
zma-miR393c-5p:zma-miR393a-5p	Li_TCONS_00080809	144-164	−39.1	−25.7	0.657289002557545
zma-miR394b-3p:zma-miR394a-3p	Boerner_Z27kG1_10860	353-375	−38.9	−26.44	0.679691516709512
zma-miR394b-3p:zma-miR394a-3p	Li_TCONS_00072326	83-101	−38.9	−25.7	0.660668380462725
zma-miR394b-3p:zma-miR394a-3p	Li_TCONS_00089213	283-304	−38.9	−26.3	0.676092544987147
zma-miR394b-5p:zma-miR394a-5p	Boerner_Z27kG1_06309	454-473	−39.1	−26.3	0.672634271099744
zma-miR394b-5p:zma-miR394a-5p	Boerner_Z27kG1_10706	340-363	−39.1	−27.3	0.698209718670077
zma-miR395b-5p	Li_TCONS_00047895	156-179	−38.6	−28.68	0.743005181347150
zma-miR395b-5p	Li_TCONS_00047896	156-179	−38.6	−28.68	0.743005181347150
zma-miR395c-5p	Li_TCONS_00080157	364-381	−40.6	−27.7	0.682266009852217
zma-miR395i-5p	Li_TCONS_00047895	156-179	−40.4	−31.38	0.776732673267327
zma-miR395i-5p	Li_TCONS_00047896	156-179	−40.4	−31.38	0.776732673267327
zma-miR395k-5p	Boerner_Z27kG1_14168	189-212	−36.2	−23.6	0.651933701657459
zma-miR395k-5p	Boerner_Z27kG1_16395	212-235	−36.2	−23.6	0.651933701657459
zma-miR395k-5p	Boerner_Z27kG1_21452	380-403	−36.2	−23.6	0.651933701657459
zma-miR395k-5p	Li_TCONS_00051216	53-77	−36.2	−26.02	0.718784530386740
zma-miR395l-5p	Li_TCONS_00062888	247-268	−38.4	−28.6	0.744791666666667
zma-miR395n-5p	Li_TCONS_00047895	156-179	−38.3	−28.38	0.740992167101828
zma-miR395n-5p	Li_TCONS_00047896	156-179	−38.3	−28.38	0.740992167101828
zma-miR396b-3p:zma-miR396a-3p	Li_TCONS_00019831	289-309	−32.9	−23.4	0.711246200607903
zma-miR396g-5p:zma-miR396h	Boerner_Z27kG1_02332	1122-1144	−35.3	−23	0.651558073654391
zma-miR396g-5p:zma-miR396h	Li_TCONS_00081264	211-233	−35.3	−23.78	0.673654390934844
zma-miR398b-3p:zma-miR398a-3p	Li_TCONS_00081462	279-299	−45.3	−30.08	0.664017660044150
zma-miR399d-5p	Li_TCONS_00097416	279-299	−44.2	−28.88	0.653393665158371
zma-miR399e-5p	Boerner_Z27kG1_08283	467-487	−41.4	−30.6	0.739130434782609
zma-miR399e-5p	Li_TCONS_00097327	326-349	−41.4	−27	0.652173913043478
zma-miR399g-5p	Boerner_Z27kG1_13975	327-345	−49.4	−35.6	0.720647773279352
zma-miR444a:zma-miR444b	Boerner_Z27kG1_21675	448-466	−35.7	−25.2	0.705882352941176
zma-miR482-3p	Boerner_Z27kG1_19929	311-332	−35.2	−28.4	0.806818181818182
zma-miR482-3p	Li_TCONS_00031436	121-145	−35.2	−30.2	0.857954545454545
zma-miR482-3p	Li_TCONS_00056585	185-206	−35.2	−23.4	0.664772727272727
zma-miR482-3p	Li_TCONS_00072944	771-793	−35.2	−25.3	0.718750000000000
zma-miR482-3p	Li_TCONS_00097147	282-301	−35.2	−24.4	0.693181818181818
zma-miR482-5p	Li_TCONS_00011383	222-239	−36.7	−24.1	0.656675749318801
zma-miR482-5p	Li_TCONS_00024356	309-331	−36.7	−24.2	0.659400544959128
zma-miR482-5p	Li_TCONS_00074927	138-158	−36.7	−24.38	0.664305177111717
zma-miR528a-3p:zma-miR528b-3p	zhang_TCONS_00045504	663-688	−42.2	−30.6	0.725118483412322

^a^miRNA data from miRBase 21.0. ^b^Decoyed lincRNA genes for the miRNAs. ^c^The starting and terminating sites in the lincRNAs when it is bound by the miRNAs. ^d^MFE of a perfectly matched site. ^e^MFE of the alignments. ^f^MFEsite/MFEperfect, the calculation based on the method in Tafer et al [50]

### Conservation of lincRNAs as miRNA decoys between six monocotyledons

**Fig. 3:**
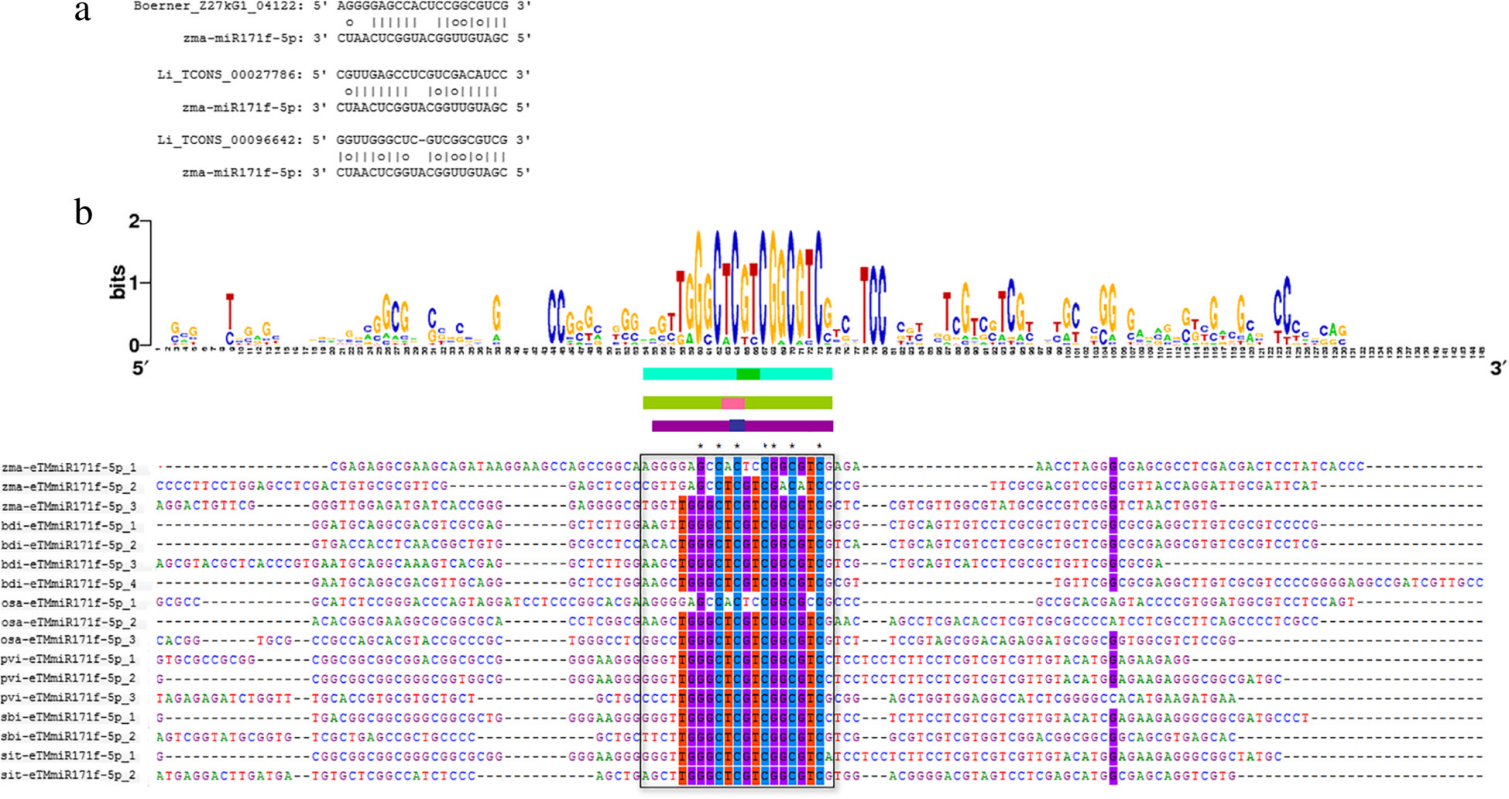
zma-miR171f-5p decoy sites in lincRNAs in maize. **a** The predicted alignments between zma-miR171f-5p and its decoys in lincRNAs. **b** Sequence alignments of decoys in lincRNAs and the surrounding regions for zma-miR171f-5p in maize and 3 other species. The decoy sites pairing with zma-miR171f-5p are underlined with a black box. The conservation status of the sequences was analyzed and presented using Weblogo (bdi: B. distachyon; osa: O. sativa; pvi: P. virgatum; sbi; S. bicolor; sit: S. italica.)

Similar to the analysis of the conservation of lincRNAs as miRNA targets, a conservation analysis of lincRNAs acting as miRNA decoys was also performed between the lincRNA regions that paired with miRNAs. Altogether, 10 of 58 miRNAs showed conserved decoy regions in lincRNAs among four to six species. For example, the sequence logo and multiple sequence alignment of zma-miR171f-5p decoys provide a precise description of the conservation of decoy regions (Fig. 3). Except for the zma-miR159e-3p and zma-miR482-3p decoys, other lincRNAs as miRNA decoy sites were conserved, but all of the surrounding regions were non-conserved (Additional file 7).

### LincRNAs may participate in miRNA-lincRNA-mRNA networks

**Fig. 4:**
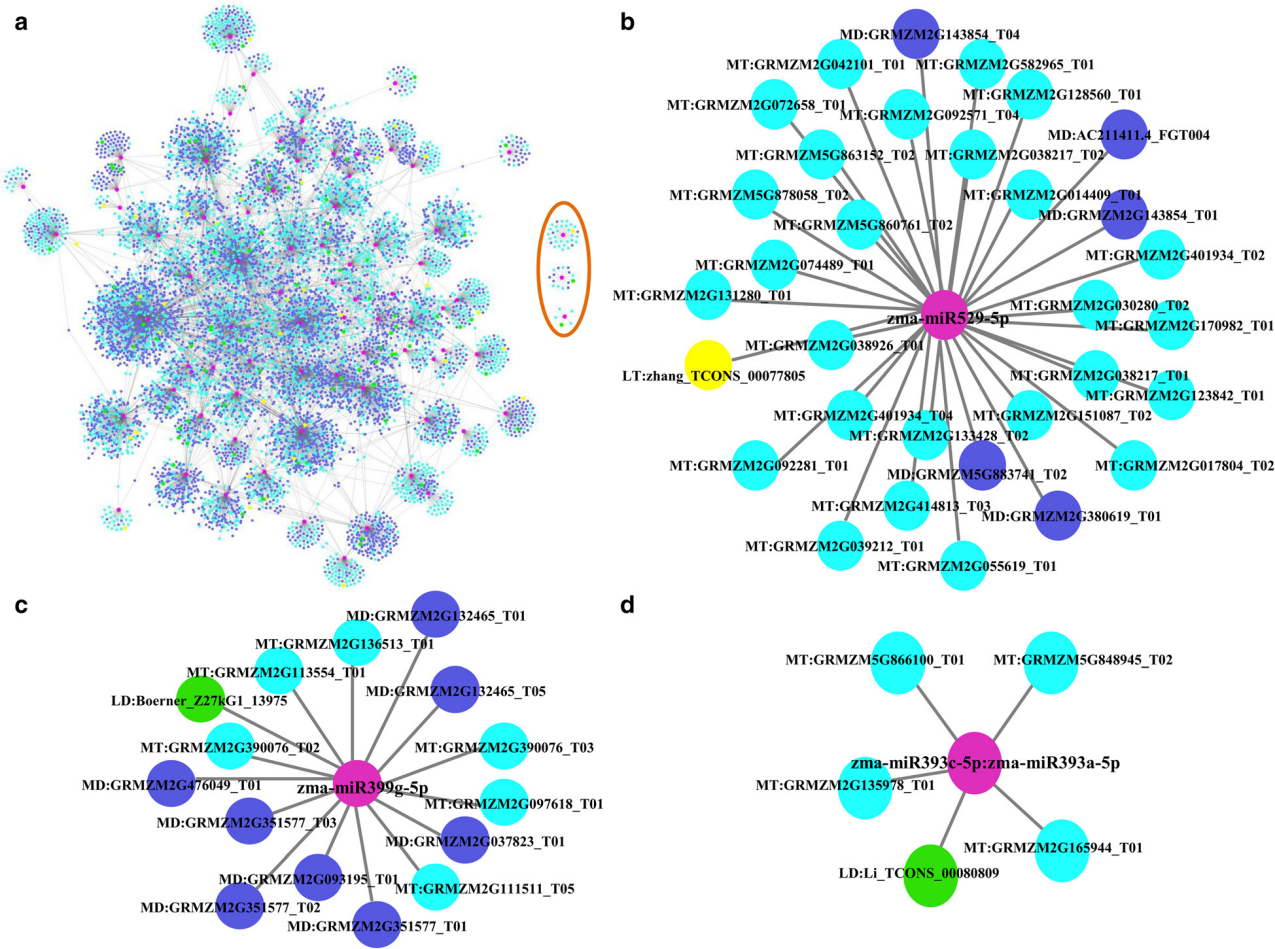
Genome-wide miRNA-regulated networks. Pink nodes: miRNAs. Yellow nodes: lincRNAs that may be miRNA targets. Green nodes: lincRNAs that may be miRNA decoys. Cyan nodes: mRNAs that may be miRNA targets. Blue nodes: mRNAs that may be miRNA decoys. Grey edges: correlations. **b**, **c** and **d** were extracted from (**a**)

Previous research has demonstrated that engineered miRNA decoys can affect the regulation of miRNAs in plants [39, 52, 67]. To investigate the function of lincRNAs acting as miRNA targets or miRNA decoys, comprehensive genome-wide networks mediated by miRNAs were constructed. The networks were composed of 9402 nodes and 10,529 edges, and the nodes included 78 miRNAs, 117 lincRNAs (lincRNAs acting as miRNA targets, lincRNAs acting as miRNA decoys) and 8834 mRNAs (mRNAs acting as miRNA targets, mRNAs acting as miRNA decoys) (Fig. 4, Additional file 8). There were 42 interactions between miRNAs and lincRNAs acting as miRNA targets, which included 33 miRNAs and 34 lincRNAs, and 104 interactions between miRNAs and lincRNAs acting as miRNA decoys, which included 58 miRNAs and 86 lincRNAs. Moreover, 3714 mRNAs as 78 miRNA targets and 5490 mRNAs as 78 miRNA decoys are also shown. Interestingly, we found that the majority of nodes participated in other miRNA-regulated networks, but only three miRNAs including zma-miR529-5p, zma-miR399g-5p and zma-miR393c-5p:zma-miR393a-5p, formed separate sub-networks.

**Fig. 5:**
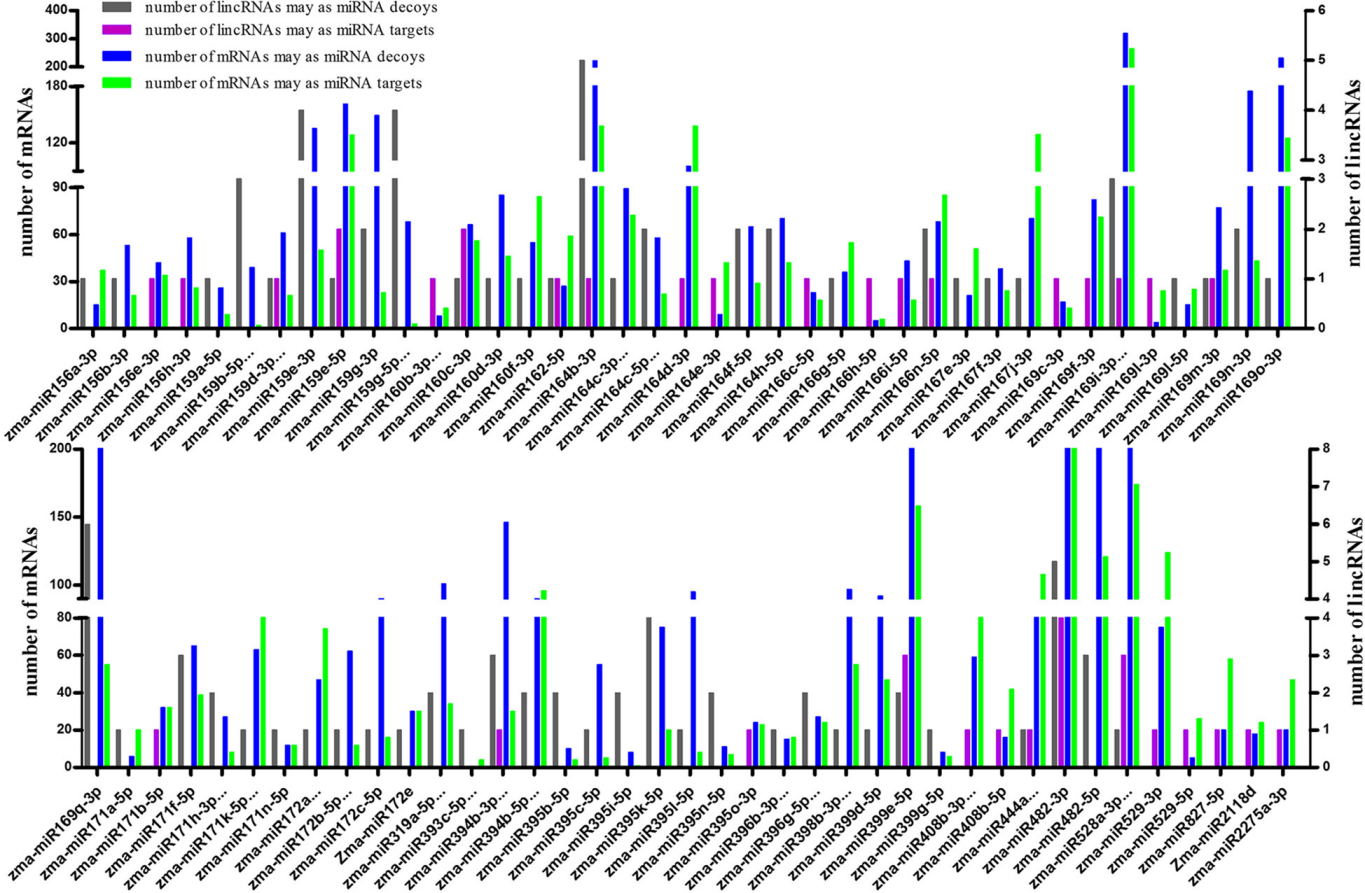
The number of alignments formed by miRNA-lincRNA and miRNA-mRNA duplexes. The X axis legend represents the miRNAs in maize. The Y axis legend indicates the number of lincRNAs or mRNAs that function as miRNA targets or decoys. The different colors of bars indicate different types of lincRNAs

To further investigate the patterns of the miRNA-lincRNA-mRNA networks, we compared the number of four types of RNAs, including lincRNAs acting as miRNA targets, lincRNAs acting as miRNA decoys, mRNAs acting as miRNA targets and mRNAs acting as miRNA decoys, and found that the numbers of the four types were unevenly distributed for each miRNA. Additionally, the number of miRNA decoys was often greater than that of miRNA targets in most sub-networks, and only a small number of sub-networks had more miRNA targets than decoys (Fig. 5).

**Fig. 6:**
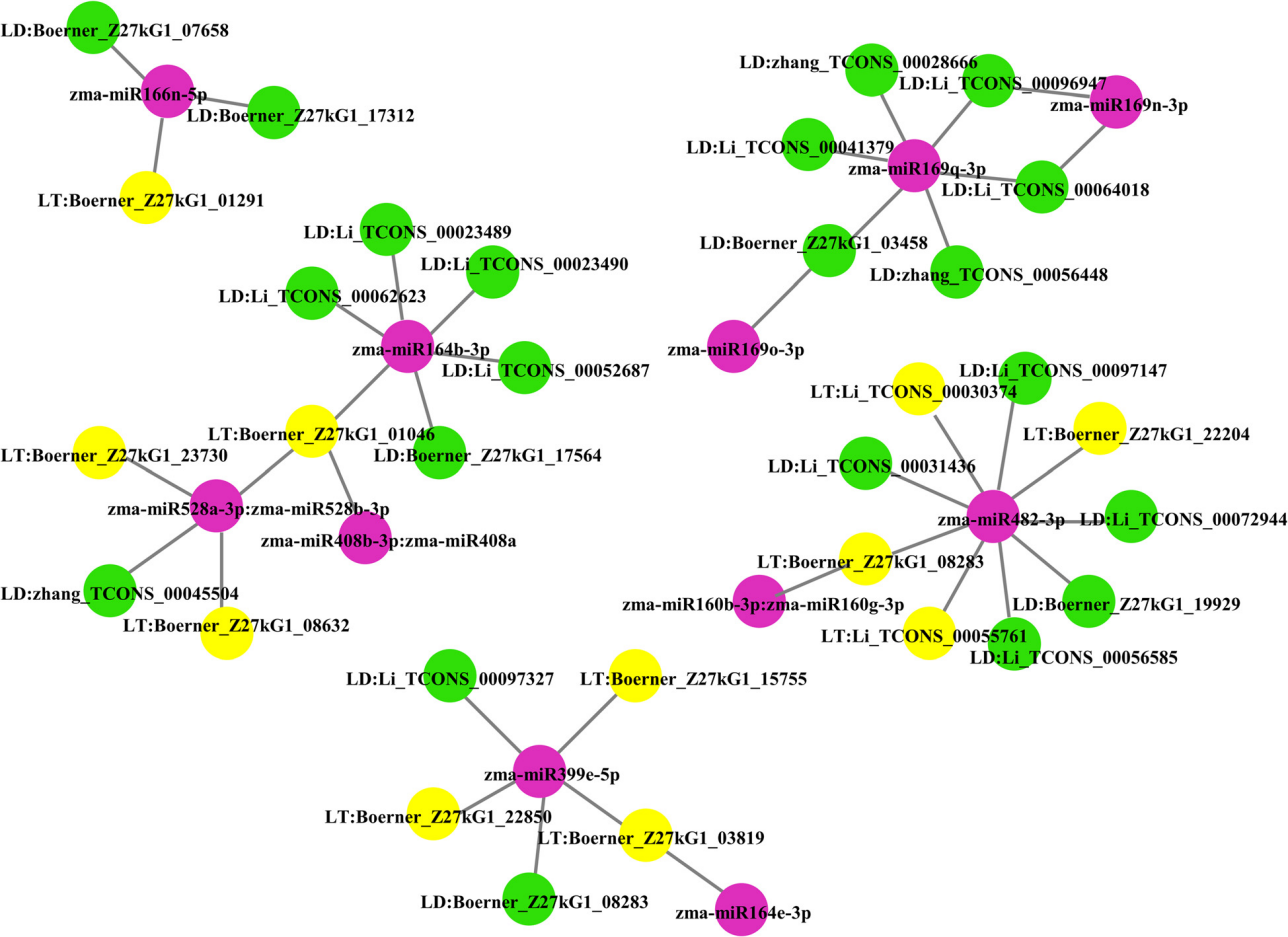
Representative regulatory networks of miRNA-lincRNA duplexes. Pink nodes: miRNAs. Yellow nodes: lincRNAs that may be miRNA targets. Green nodes: lincRNAs that may be miRNA decoys. Grey edges: correlations

Furthermore, we found that miRNAs could bind to one or more lincRNAs (Fig. 6, Additional file 9). For example, Boerner_Z27kG1_07658 and Boerner_Z27kG1_17312 acted as decoys of zma-miR166n-5p, and Boerner_Z27kG1_01291 acted as a target of this miRNA. We also found that some lincRNAs could be bound by miRNAs from the same or different miRNA families. For example, Li_TCONS_00096947 and Li_TCONS_00064018 could be bound by zma-miR169n-3p and zma-miR169q-3p, and Boerner_Z27kG1_01046 could be bound by zma-miR408b-3p:zma-miR408a, zma-miR528a-3p:zma-miR528b-3p and zma-miR164b-3p (Fig. 6). Amazingly, the same lincRNA could be used as both a miRNA target and decoy using different binding sites in the lincRNAs. For example, Boerner_Z27kG1_08283 could be a target of zma-miR160b-3p:zma-miR160g-3p and zma-miR482-3p, and it could act as a decoy for zma-miR399e-5p (Fig. 6).

### Functional prediction of lincRNAs acting as miRNA targets based on the lincRNA-mRNA co-expression network

**Fig. 7:**
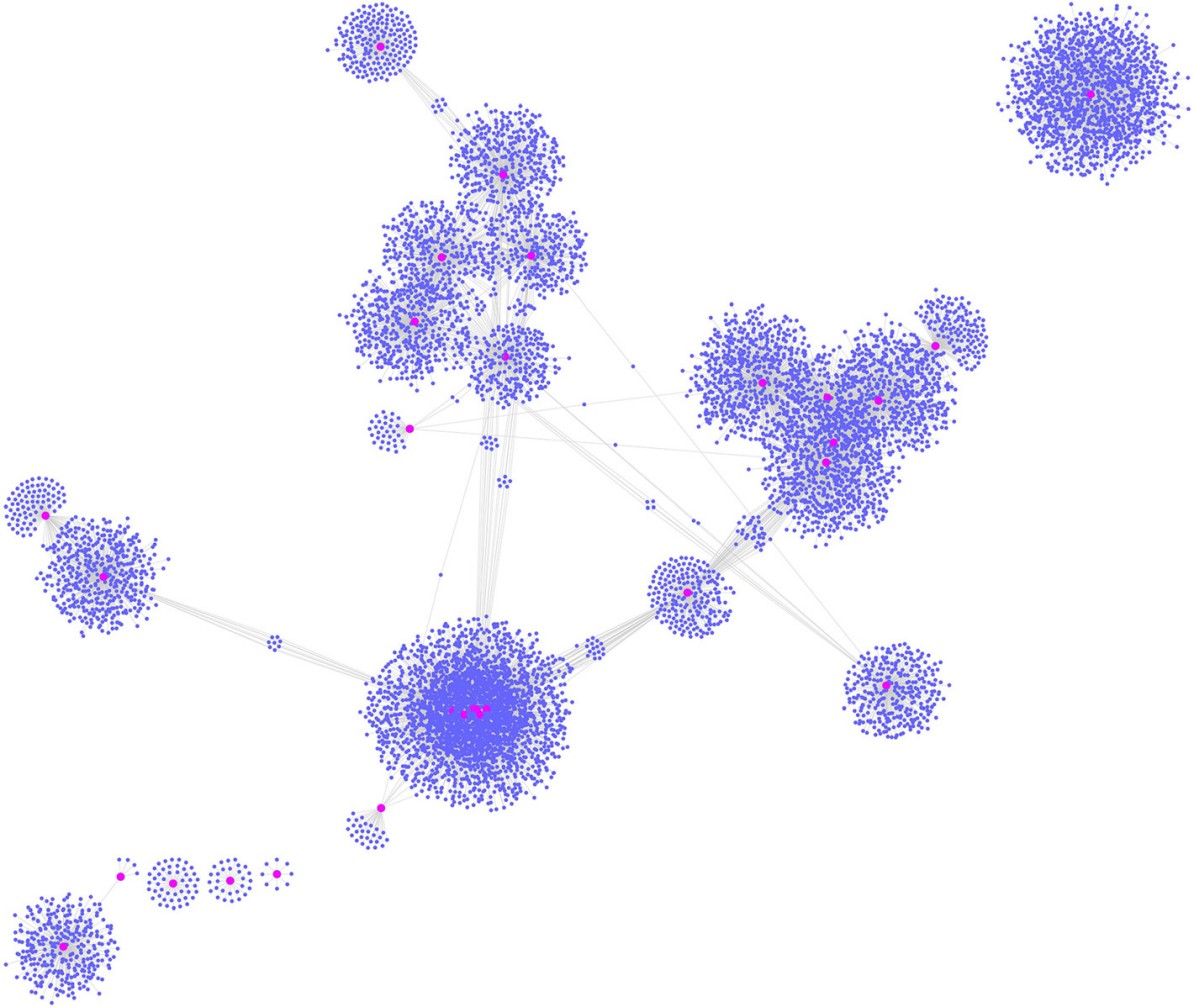
The network of lincRNAs acting as miRNA targets that are co-expressed with mRNAs. Pink nodes represent lincRNAs, and blue nodes represent mRNAs. The edges represent connected nodes that exhibit a high correlation

**Fig. 8:**
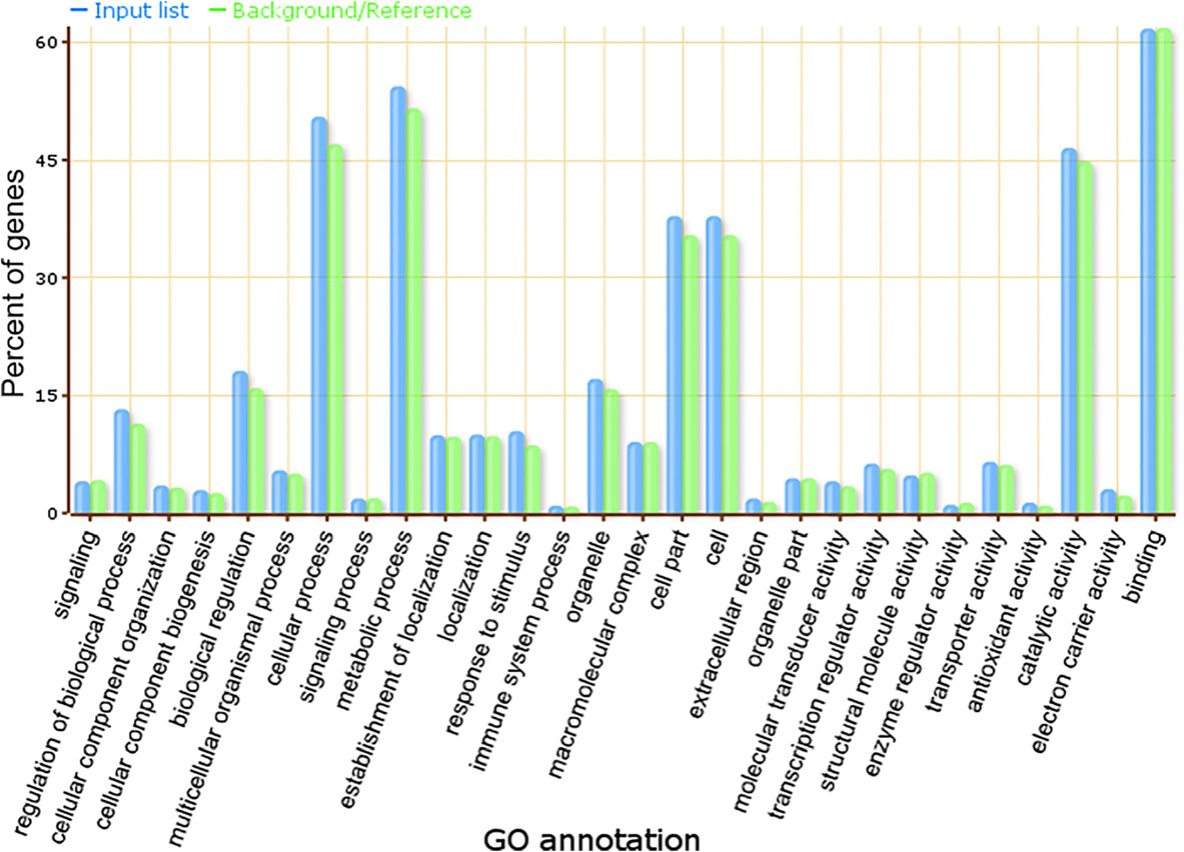
GO term enrichment analysis of lincRNAs acting as miRNA targets in maize. In the bar chart, the GO annotation is presented as the X axis legend and percent of genes as the Y axis legend. In the figure, blue bars represent the enrichment status of the mRNA GO terms among the 32 co-expressed lincRNAs, and green bars represent the percentage of the total annotated maize genes that were aligned to the GO terms. The GO analysis was performed using the AgriGO toolkit, selecting the “*Zea mays* ssp V5a” as a control set

To speculate on the functions of the 34 validated lincRNAs acting as miRNA targets, a co-expression network between lincRNAs and mRNAs was first constructed and then visualized (see materials and methods). The lincRNA-mRNA co-expression network was composed of 32 lincRNA nodes, 9043 mRNA nodes and 17968 edges (Fig. 7, Additional file 10), and we were able to infer that 32 lincRNAs could be co-expressed with 9043 mRNAs. In the network, we could see that one or more mRNAs were centered around lincRNAs and were connected to lincRNAs based on the Pearson correlation coefficient. Therefore, we could infer the function of each lincRNA based on the function of connected mRNAs. Through GO enrichment and functional analysis of the mRNAs that were co-expressed with lincRNAs, we found that lincRNAs mainly participate in cellular, metabolic and other biological processes, such as regulation of biological processes, metabolic processes, cellular processes, as well as in the response to stress (Fig. 8, Additional files 11 and 12). These lincRNAs were also highly enriched in cellular component terms including thylakoid and photosynthetic membrane (Additional files 11 and 13). Moreover, we found that the GO terms “hydrolase activity, acting on acid anhydrides”, “tetrapyrrole binding”, “iron ion binding” and “heme binding” were enriched in the “molecular function” category (Additional files 11 and 14).

### Functional prediction of lincRNAs acting as miRNA decoys based on miRNA-lincRNA-mRNA networks

**Fig. 9:**
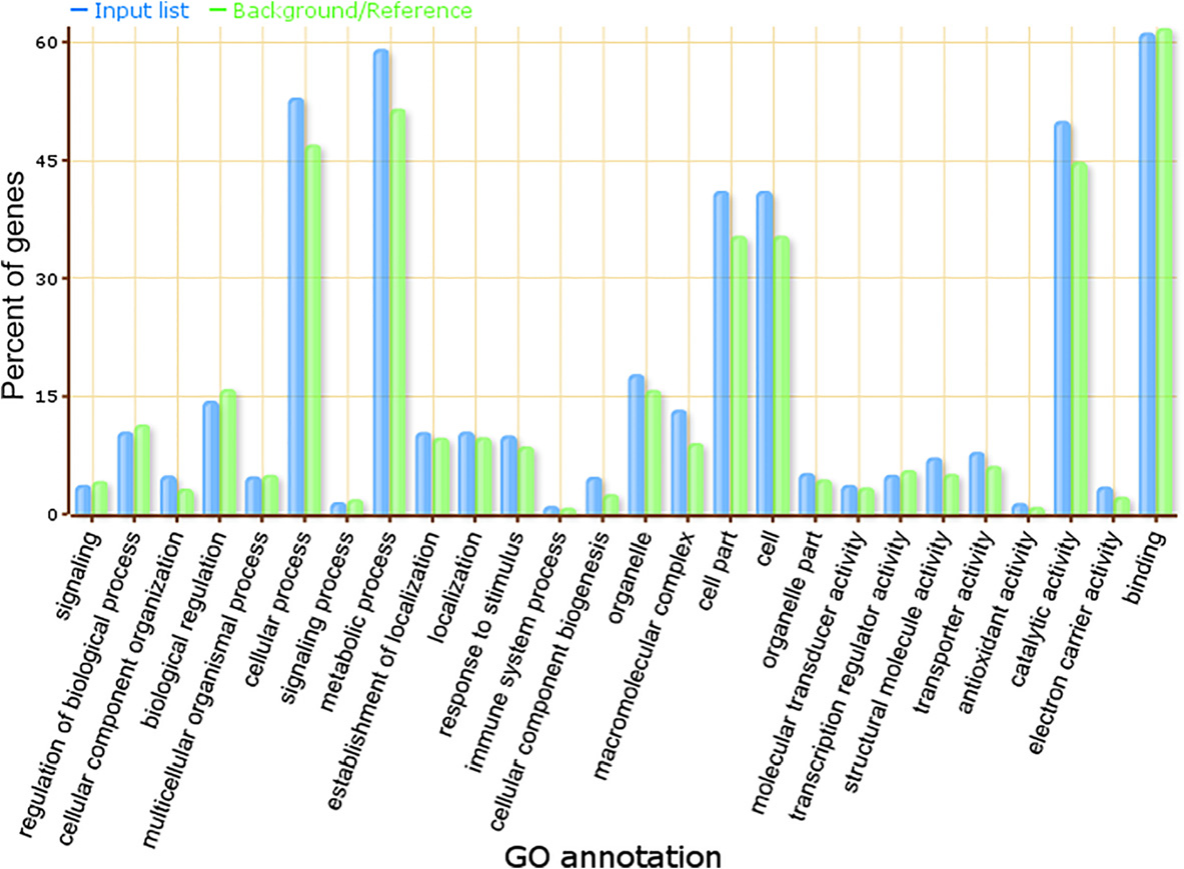
GO term enrichment analysis of lincRNAs acting as miRNA decoys in maize. In the bar chart, the GO annotation is presented on the X axis legend and the percent of genes on the Y axis legend. In the figure, blue bars represent the enrichment status of the GO terms among the 58 miRNA targets in mRNAs, and green bars represent the percentage of the total annotated maize genes that were aligned to the GO terms. The GO analysis was performed using the AgriGO toolkit, selecting the “*Zea mays* ssp V5a” as a control set

Based on the ceRNA hypothesis, which suggests that when lincRNAs acting as miRNA decoys and mRNAs are targeted by the same miRNAs, the function of the lincRNAs acting as miRNA decoys can be inferred from the mRNAs, we speculated on the function of 86 lincRNAs acting as 58 miRNA decoys. After using the AgriGO toolkit to perform GO analysis of mRNAs that could be targeted by the same miRNAs acting on lincRNAs [66], we found that lincRNAs acting as miRNA decoys were involved in multiple biological processes, participated in the formation of many cellular components, and influenced the activities of molecular functions (Fig. 9, Additional file 15). They were mainly involved in cellular and metabolic processes, and the molecular functions of lincRNAs acting as miRNA decoys were focused on catalytic activity and binding functions (Fig. 9).

To obtain a global function of lincRNAs acting as miRNA decoys, we performed enrichment analysis again, and found that these 86 lincRNAs may participate in diverse biological processes, such as cellular component organization; cellular component biogenesis; cellular processes; and metabolic processes, including macromolecular complex subunit organization, nucleosome assembly, DNA packing, superoxide metabolic processes, ribosome biogenesis, oxidation reduction, biosynthetic processes and translation (Additional file 16). They could also be involved in the formation of cells, macromolecular complexes, cell projections, cytoplasm, microtubules and protein-DNA complexes (Additional file 17). Moreover, these lincRNAs might modulate the effects of multiple molecular functions, including binding, structural molecular activity, transporter activity, catalytic activity, and electron carrier activity, and they may exhibit translation elongation factor activity, unfolded protein binding activity, monooxygenase activity, ammonia-lyase activity and GTPase activity (Additional file 18).

## Discussion

### lincRNAs can be direct miRNA targets in maize

With their importance in regulating gene expression, lincRNAs have garnered significant attention in the life science field. Although increasing lincRNAs have been predicted and identified in plants [15–22], the relationship between miRNAs and lincRNAs have seldom been investigated by comparing the mRNAs as miRNA targets [19, 33, 34]. Recently, 51 lincRNAs were identified as putative targets of 30 miRNAs in *Populus trichocarpa* [19], but the evidence of lincRNAs acting as miRNA targets in plants are still lacking.

In plants, degradome sequencing is a new technology to identify and validate targets of miRNAs [68–72], and it has been used to directly validate miRNA targets in plants. Currently, only mRNAs as miRNA targets, but not lincRNAs as miRNA targets, have been validated by degradome data. Thus, using degradome data, we validated 34 lincRNAs as 33 miRNA targets, which indicates that, similar to mRNAs acting as miRNA targets, lincRNAs can also directly act as miRNA targets.

### LincRNAs can also be miRNA decoys in maize

Functional target mimicries (miRNA decoys) were first studied in *Arabidopsis* [39]; consequently, computational methods have been used to identify miRNA decoys, but most of the identified miRNA decoys were protein-coding genes [52, 73, 74]. Only few studies were performed in ncRNAs as putative miRNA decoys [19, 41, 55], and no lincRNAs as miRNA decoys had previously been investigated in maize.

In our study, we found that a portion of lincRNAs could not be directly cleaved by the miRNA-associated silencing complex due to the existence of mismatches or large bulges at the 9^th^ to 12^th^ nucleotide positions of the miRNA-lincRNA pairing site. Using bioinformatics, we identified 86 lincRNAs acting as 58 miRNA decoys in maize and found that the miRNA decoy sites were conserved; however, most of the flanking regions of the miRNA decoy sites were not conserved. Our results indicate that lincRNAs acting as miRNA decoys widely exist in plants, which supports previously published data that lincRNAs as miRNA decoys could also be regulators of miRNA [19, 41].

### The potential function of lincRNAs as miRNA targets or decoys

To investigate the function of maize lincRNAs acting as miRNA targets or decoys, two methods were used in this study: a co-expression network and the ceRNA hypothesis. The co-expression network, which is commonly used to predict gene function [64, 75, 76], was used to predict the function of lincRNAs as miRNA targets. By using the co-expression network, we predicted the function of 32 maize lincRNAs, and these lincRNAs were enriched in signaling processes, the regulation of biological processes, multicellular organismal processes, metabolic processes and immune system processes. Interestingly, these lincRNAs were enriched in multiple molecular functions, mainly in the catalytic activity and binding categories. Furthermore, when comparing with drought response lincRNAs previously reported, we found that three lincRNAs as miRNA targets in stress category were differentially expressed between the control and drought-stressed leaves (Additional file 19), which indicated that lincRNAs as miRNA targets may be involved in drought-stress [22].

The ceRNA hypothesis implies a network relationship between miRNAs, lincRNAs as miRNA decoys, and mRNA as miRNA targets; in these networks, lincRNAs could act as miRNA decoys, sequestering miRNAs and thereby favoring the expression of repressed mRNA targets [36, 77], and such networks can be used to predict the function of lincRNAs as miRNA decoys. Here, the functions of 86 lincRNAs acting as 56 miRNA decoys were predicted, and it was found that they can inhibit miRNA functions in a spatial- or temporal-specific manner, thus contributing to the regulation of transcript complexity in maize. Furthermore, when comparing the lincRNAs as miRNA decoys in the stress category using the previously reported drought response lincRNAs, 7 lincRNAs as miRNA decoys had been investigated previously and were differentially expressed between the control and drought-stressed leaves (Additional file 19), which indicated that lincRNAs associated with drought stress could potentially regulate miRNAs through lincRNAs as miRNA decoys.

Of the 1831 identified lincRNAs in maize, the number of lincRNAs that had the inferred function (34 lincRNAs as miRNA target, 86 lincRNAs as miRNA decoys) was still limited, which is consistent with the diverse mechanism of action of lincRNAs [15, 22]. We think that the lincRNAs as miRNA targets or miRNA decoys are just one type of lincRNAs, and we hope to investigate the function of other types of lincRNAs by using other methods, such as lincRNA-protein interaction prediction. In summary, our study lays a solid foundation for elucidating the regulatory mechanisms of miRNAs in maize and provides a source for exploring the function of lincRNAs in the future.

## Conclusions

This study employed a computational pipeline for the systematic analysis of putative miRNA-lincRNA duplexes to better understand the role of lincRNAs. We found that 42 miRNA-lincRNA duplexes remained after filtering based on degradome evidence, and they were composed of 33 miRNAs and 34 lincRNAs that may be directly cleaved by miRNAs. Furthermore, 32 of the 34 lincRNAs could be co-expressed with mRNAs, and 86 lincRNAs were predicted as miRNA decoys that may competitively bind to miRNAs. According to the obtained co-expression networks and the ceRNA hypothesis, we effectively predicted the function of lincRNAs as miRNA targets or decoys. Future experimental studies are required to elucidate the mechanisms of miRNA-lincRNA duplexes and to reveal the functions of these lincRNAs in plants.

### Availability of supporting data

The datasets supporting the results of this article are included within the article and its additional files.

## Additional files

Additional file 1:
**LincRNA information derived from three articles.** (XLS 20 kb) Additional file 2:
**Summary of degradome reads in** ***Zea mays*** **.** (XLS 20 kb) Additional file 3:
**LincRNAs predicted as putative miRNA targets.** (XLS 344 kb) Additional file 4:
**The number of degradome reads that are perfectly matched with lincRNAs identified as miRNA targets.** (XLS 2627 kb) Additional file 5:
**The sequence logos of the 12 conserved lincRNAs as miRNA targets.** (ZIP 3605 kb) Additional file 6:
**LincRNAs predicted as miRNA decoys.** (XLS 71 kb) Additional file 7:
**The sequence logos of the 10 conserved lincRNA as miRNA decoys.** (ZIP 1503 kb) Additional file 8:
**The nodes and edges information of miRNA-lincRNA-mRNA networks.** (XLS 755 kb) Additional file 9:
**The nodes and edges information of miRNA-lincRNA networks.** (XLS 31 kb) Additional file 10:
**The nodes and edges information of lincRNA-mRNA co-expression networks.** (XLS 1090 kb) Additional file 11:
**The potential function of lincRNAs as miRNA targets.** (XLS 5756 kb) Additional file 12:
**GO enrichment analysis of maize lincRNAs functioning as miRNA targets associated with “biological processes”.** (PDF 153 kb) Additional file 13:
**GO enrichment analysis of maize lincRNAs functioning as miRNA targets associated with “cellular components”.** (PDF 50 kb) Additional file 14:
**GO enrichment analysis of maize lincRNAs functioning as miRNA targets associated with “molecular functions”.** (PDF 65 kb) Additional file 15:
**The potential function of lincRNAs as miRNA decoys.** (XLS 1841 kb) Additional file 16:
**GO enrichment analysis of maize lincRNAs functioning as miRNA decoys associated with “biological processes”.** (PDF 1350 kb) Additional file 17:
**GO enrichment analysis of maize lincRNAs functioning as miRNA decoys associated with “cellular components”.** (PDF 773 kb) Additional file 18:
**GO enrichment analysis of maize lincRNAs functioning as miRNA decoys associated with “molecular functions”.** (PDF 1498 kb) Additional file 19:
**The expression of drought-stressed lincRNAs as miRNA targets or decoys.** (XLS 22 kb)

## Abbreviations

lincRNAs: Long intergenic noncoding RNAs
ncRNAs: Non-coding RNAs
miRNAs: microRNAs
nt: Nucleotide
lncRNAs: Long non-coding RNAs
CDS: Coding sequence
ORF: Open reading frame
ceRNAs: Competing endogenous RNAs
GEO: Gene expression omnibus
MFE: Minimum free energy
GO: Gene ontology
