# Supplementary material for: Genome-wide identification and functional analysis of lincRNAs acting as miRNA targets or decoys in maize
Source: BMC Genomics. 2015 Oct 15;16:793. doi: 10.1186/s12864-015-2024-0 (PMC4608266; doi:10.1186/s12864-015-2024-0)

```
Boerner_Z27kG1_01046: 5' GGUGGACGAGGUGGUGCAUGG 3'
                         |||||| o||o||| |||oo
     zma-miR164b-3p: 3' CCACCUCUUCUACC-CGUGUA 5'
```

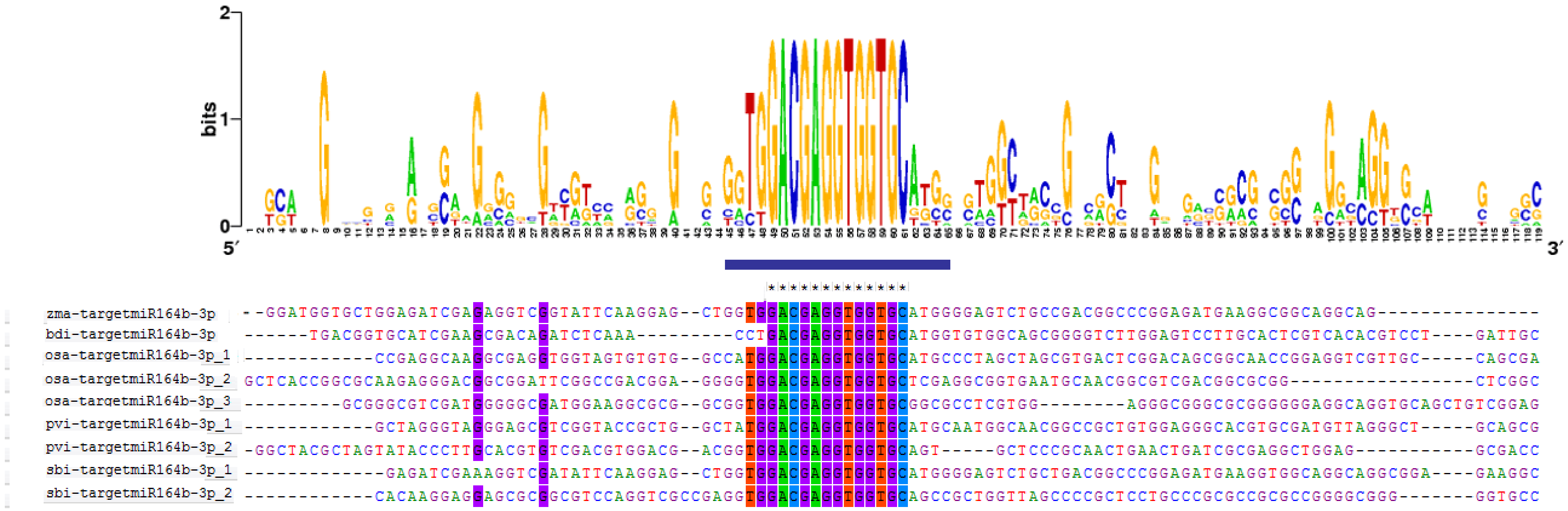

Supplement: Additional file 5: — The sequence logos of the 12 conserved lincRNAs as miRNA targets. (ZIP 3605 kb) [file 12864_2015_2024_MOESM5_ESM.zip › Additional file 5/target-164b-3p.pdf]
