# Supplementary material for: Genome-wide identification and functional analysis of lincRNAs acting as miRNA targets or decoys in maize
Source: BMC Genomics. 2015 Oct 15;16:793. doi: 10.1186/s12864-015-2024-0 (PMC4608266; doi:10.1186/s12864-015-2024-0)
Supplement: Additional file 5: — The sequence logos of the 12 conserved lincRNAs as miRNA targets. (ZIP 3605 kb) [file 12864_2015_2024_MOESM5_ESM.zip › Additional file 5/target-166n-5p.pdf]

```
Boerner_Z27kG1_01291: 5' CACCGAGUCGAGGUCGAC-GUCC 3'
                         |||||||o| ||o |o|| o|||
      zma-miR166n-5p: 3' GUGGCUCGG-UCU-GUUGUUAGG 5'
```

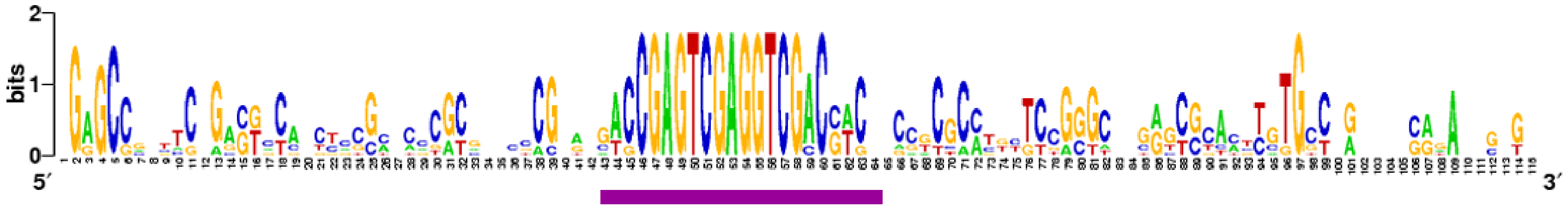


```
                         ********** ****  *                                   *
zma-targetmiR166n-5p     [illegible]GA-CGAGGAGAAGGGGCAGGCACCAGAGCACGAAGTCG-AGCACCGAGTCGAGGTCGACGTCCCAGTCCCACTC------CCACACCCTCAGCATGGCCATTGGGAG-------
bdi-targetmiR166n-5p_1   ---------TCCGACTCCCCCTCCGCCCCCGCGGTCCCG-TCGACCGAGTCGAGGTCGACCACGGCCGCGCCTGTTCCGGGCGTGGGCGCACTTGTGCCTGCAACCGT-----GA
bdi-targetmiR166n-5p_2   -----------GGAGGGTGTTCGTGAAAATGCCCGACCG-GGATGCGAGTCGAGGTCGACGTGTTGAATGCACTCGTGACTATGTATTCCAAGTGTGCTGATGTTGAG---GTTG
bdi-targetmiR166n-5p_3   AGAGCCGGTTCCGCCTCCACCTCCGCCCCCGCGACCTCG-ATGACCGAGTCGAGGTCGACCACAGCCGCGCCTGTTCTGGGCACGGGCGCACTTGTGCCTG--------------
bdi-targetmiR166n-5p_4   AGAGCCGTTTCCGACTCCACCTCCGCCCCCGCAGTCCCG-ACGACCGAGTCGAGGTCGACCACGACCGCGCCTGTTCCGGGCGCGAGCGCACTTGTGCCTG--------------
bdi-targetmiR166n-5p_5   GGGGCCGTTTCTGACTCCACCTCCGCCCCCGCGGTCCCG-ACGACCGAGTCGAGGTCGACCACGGCCTCGCCTAATCCGCGCGCGGGCGCACTTGTGCCTG--------------
pvi-targetmiR166n-5p     -------------AGGATAATCAGGTTCGGGTAATTGAGTGAGTCCGAGTCGAGGTCGACGTCATCGTCTAAGTC--CGGGTAGGAGTCTAACCCGGGTAGGAGTCGAACCGAGG
sit-targetmiR166n-5p     CG--CCCACCCGGGGGACCACGGGCAGTACATCTCCGCC-GGCACCGAGTCGAGGTCGCCGTCCTCCGC------------CGGCGTCGGTTCCCTGTCCACCAGCAGAAGCCGC
```
