# Supplementary material for: Genome-wide identification and functional analysis of lincRNAs acting as miRNA targets or decoys in maize
Source: BMC Genomics. 2015 Oct 15;16:793. doi: 10.1186/s12864-015-2024-0 (PMC4608266; doi:10.1186/s12864-015-2024-0)
Supplement: Additional file 5: — The sequence logos of the 12 conserved lincRNAs as miRNA targets. (ZIP 3605 kb) [file 12864_2015_2024_MOESM5_ESM.zip › Additional file 5/target-169l-3p.pdf]

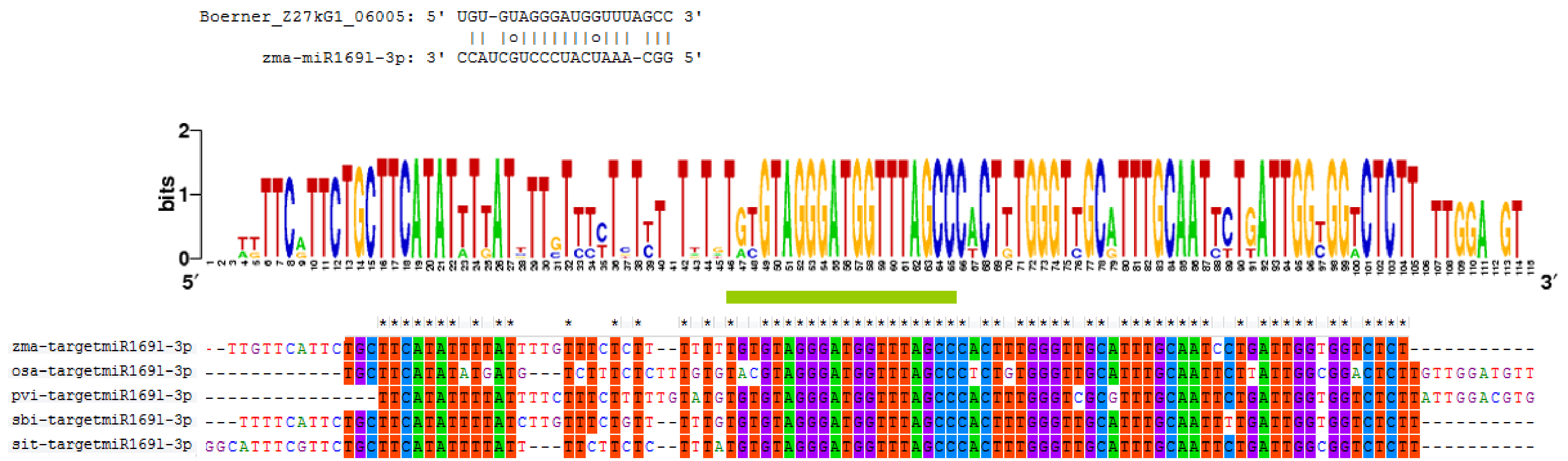

Boerner_Z27kG1_06005: 5' UGU-GUAGGGAUGGUUUAGCC 3'
zma-miR1691-3p: 3' CCAUCGUCCCUACUAAA-CGG 5'
bits
5′
3′
zma-targetmiR1691-3p
osa-targetmiR1691-3p
pvi-targetmiR1691-3p
sbi-targetmiR1691-3p
sit-targetmiR1691-3p
