# Supplementary material for: Genome-wide identification and functional analysis of lincRNAs acting as miRNA targets or decoys in maize
Source: BMC Genomics. 2015 Oct 15;16:793. doi: 10.1186/s12864-015-2024-0 (PMC4608266; doi:10.1186/s12864-015-2024-0)
Supplement: Additional file 5: — The sequence logos of the 12 conserved lincRNAs as miRNA targets. (ZIP 3605 kb) [file 12864_2015_2024_MOESM5_ESM.zip › Additional file 5/target-2118d.pdf]

```
Boerner_Z27kG1_20838: 5' AAGGCGUGGGACGGCAACGGCAU 3'
                            ||||o||||| |||| |o| |
        zma-miR2118d: 3' AUCCGUACCCU-CCGUAGUCCUU 5'
```

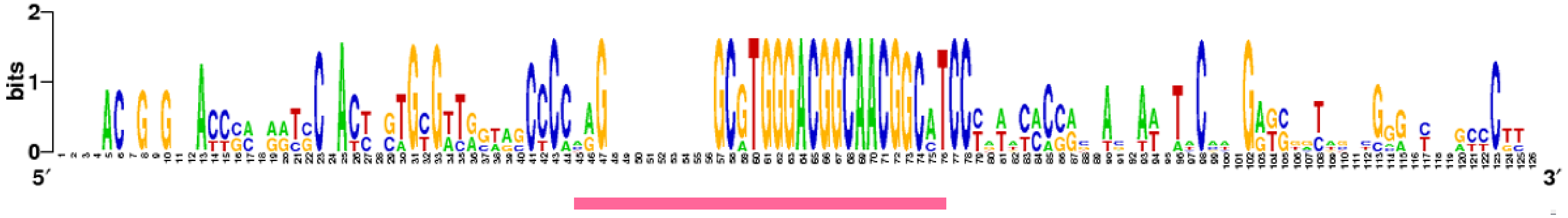


```
zma-targetmiR2118d     -----------------------------------------CCCAAG---------GCGTGGGACGGCAACGGCATCCCGAACACCACAACCAAATACAAGGAGGACCAGAAGG------------
bdi-targetmiR2118d_1   -------------CCCCGGGTGCCACTGGTGCGACAGTAGCCCCCGG---------GCGTGGGACGGCAACG----CCTGTTCCCGGGTAGGATCTTCCCAGGTCGGTTGTCGGGCTACGCCCTTA
bdi-targetmiR2118d_2   ----ACGGTGGTACCCCGGGTGCCACTTGTGCGATAGTAGCCCCTGG---------GCGTGGGACGGCAACG----CCTATTCCCGGGCAAAATCATCCTTGGTCGGTTGCTGGGCT---------
sbi-targetmiR2118d_1   TTCAACTGGGAAACCGACAACCCAACTCCTGTGTTG-----CCCAAG---------GCATGGGACGGCAACGGCATCCCAAATACCACTACCAAATACAAAGAGGTATATATCA------------
sbi-targetmiR2118d_2   ------------------------ATCGGAGCGTTGCAGCCTCACAGTGTCAGGTCGCGTGGGACGGCAACGGCCTCCTTTGTCACGTGTGTTTGTCCGTCGATCGGCACCCGGGAC--GCTCGTC
sit-targetmiR2118d     ------------ATTGATAATCCTACCCCTGTGTTG-----CCCAAG---------GCGTGGGACGGCAACGGCATCCCAAACACCACAACAAAGTACAAAGAGGTATGCTCGCATCTGATCCTCT
```
