# Supplementary material for: Genome-wide identification and functional analysis of lincRNAs acting as miRNA targets or decoys in maize
Source: BMC Genomics. 2015 Oct 15;16:793. doi: 10.1186/s12864-015-2024-0 (PMC4608266; doi:10.1186/s12864-015-2024-0)

```
Boerner_Z27kG1_20838: 5' AGGCGUGGGACGGCAACGGCA 3'
                         |o|| ||o|||oo|||| |||
       zma-miR444a/b: 3' UUCGAACUCUGUUGUUGACGU 5'
```

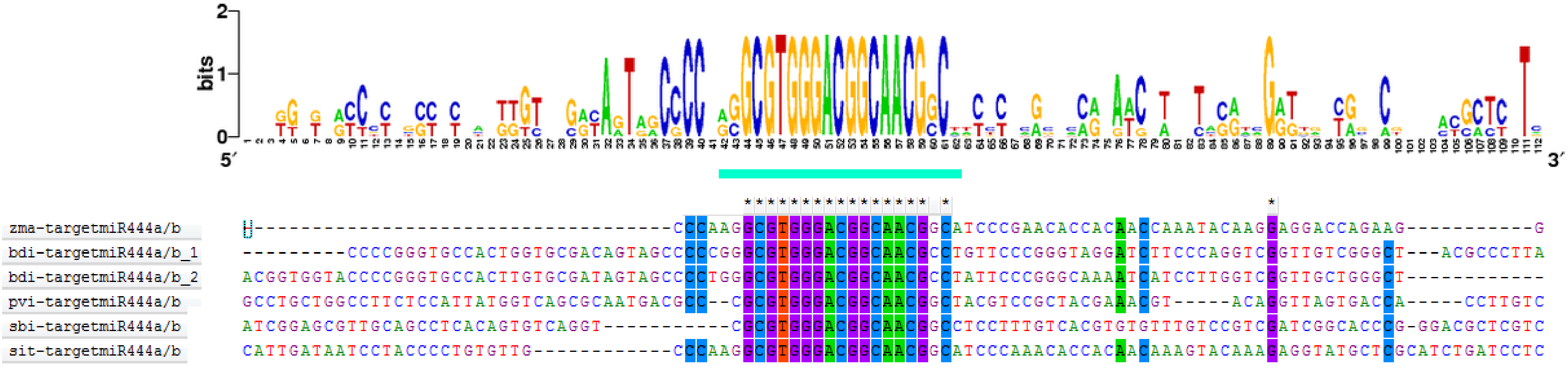

Supplement: Additional file 5: — The sequence logos of the 12 conserved lincRNAs as miRNA targets. (ZIP 3605 kb) [file 12864_2015_2024_MOESM5_ESM.zip › Additional file 5/target-444a_444b.pdf]
