# Supplementary material for: Genome-wide identification and functional analysis of lincRNAs acting as miRNA targets or decoys in maize
Source: BMC Genomics. 2015 Oct 15;16:793. doi: 10.1186/s12864-015-2024-0 (PMC4608266; doi:10.1186/s12864-015-2024-0)
Supplement: Additional file 5: — The sequence logos of the 12 conserved lincRNAs as miRNA targets. (ZIP 3605 kb) [file 12864_2015_2024_MOESM5_ESM.zip › Additional file 5/target-482-3p.pdf]

```
   Li_TCONS_00030374: 5' AGAGGGAGGGGGGGGGGGG 3'
                         |o ||||||oo oo||oo|o
        zma-miR482-3p: 3' UUACCCUCCUUGUUCCUUCU 5'

   Li_TCONS_00055761: 5' UGAGGGAGGGACAAGGUGGC 3'
                          o ||||||o|||||| o|
        zma-miR482-3p: 3' UUACCCUCCUUGUUCCUUCU 5'

 Boerner_Z27kG1_22204: 5' AGGGGGAGGAG-GAGGAGGA 3'
                          |o |||||||o o||||o||
        zma-miR482-3p: 3' UUACCCUCCUUGUUCCUUCU 5'

 Boerner_Z27kG1_08283: 5' AAGGGGACGAGGGCGAGGAGGA 3'
                          || ||||  |  |oo|o||||o||
        zma-miR482-3p: 3' UUACCCU-C-CUUGUUCCUUCU 5'
```

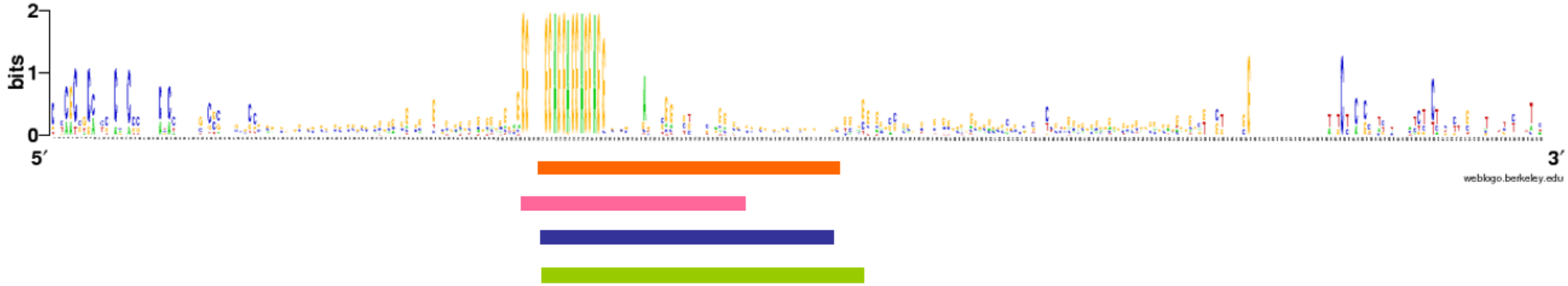

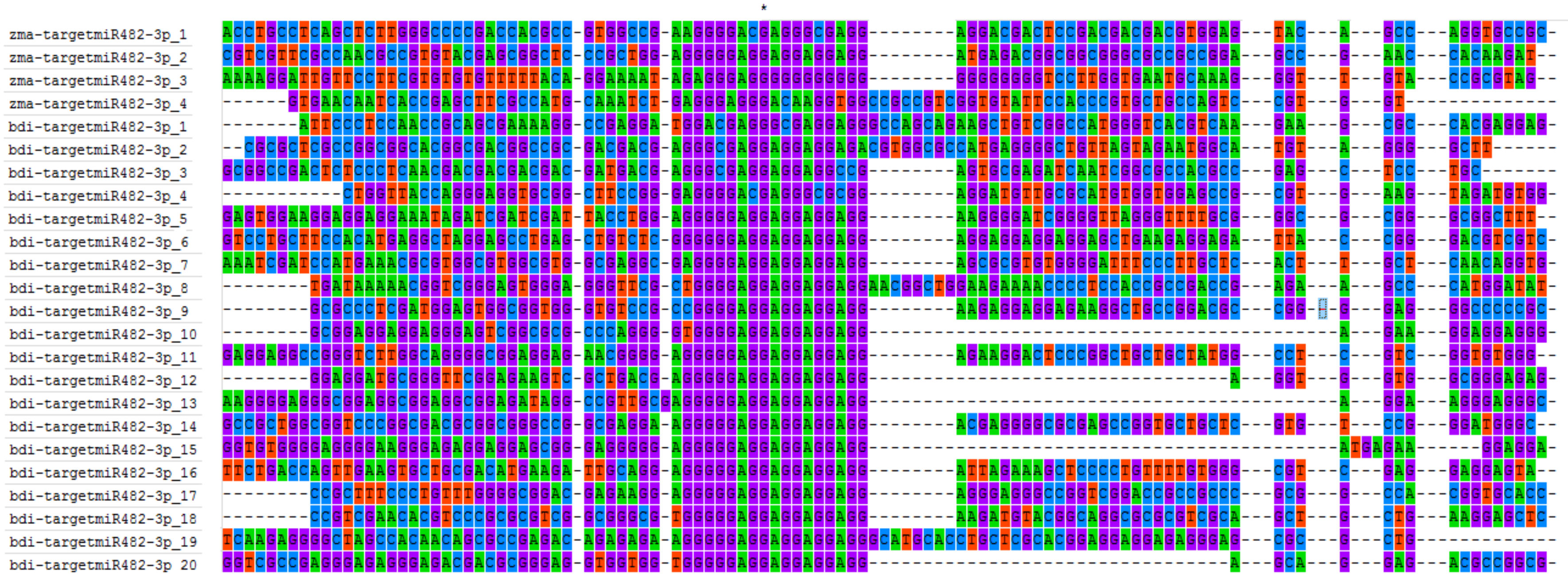

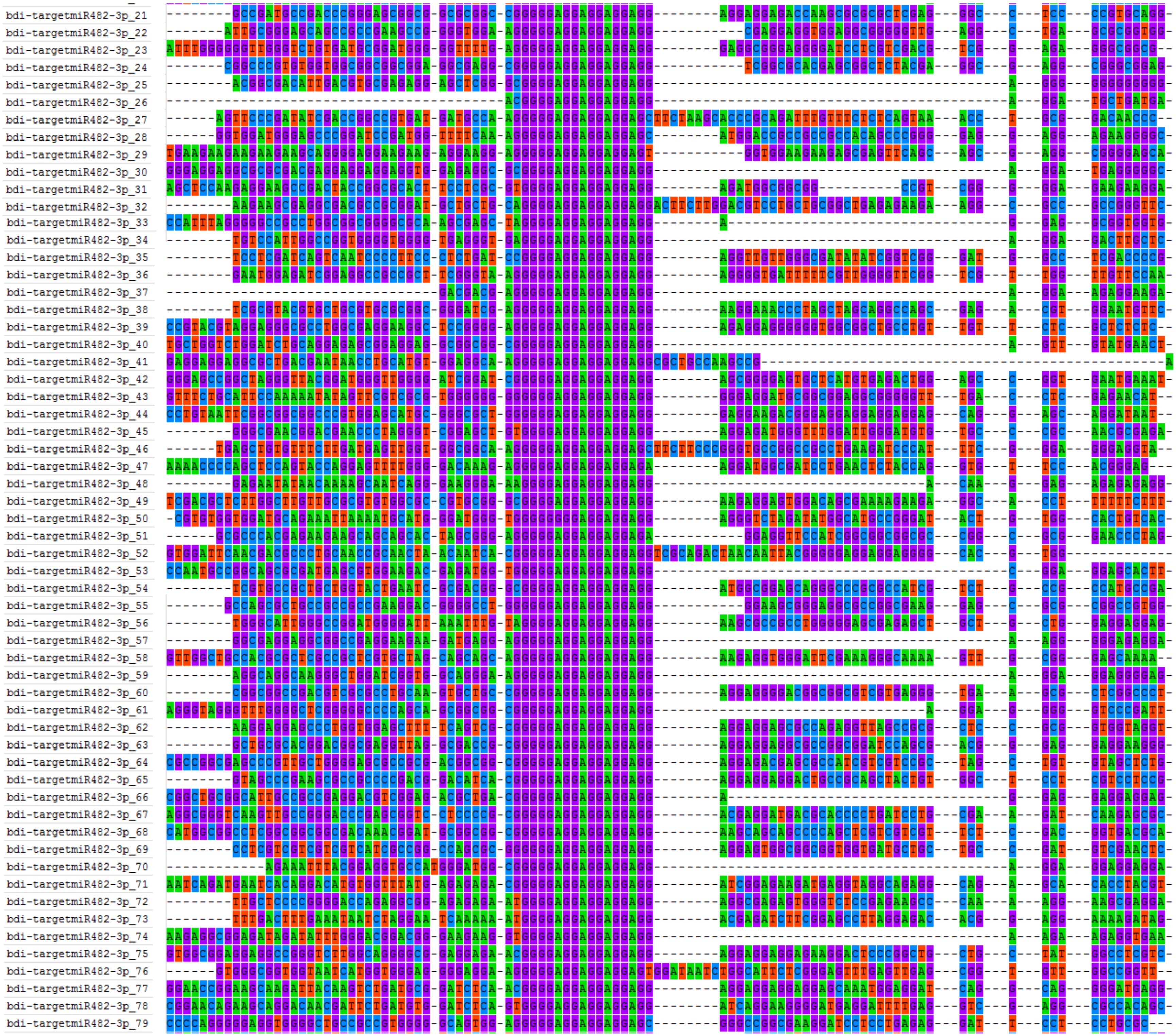
bdi-targetmiR482-3p_21
bdi-targetmiR482-3p_22
bdi-targetmiR482-3p_23
bdi-targetmiR482-3p_24
bdi-targetmiR482-3p_25
bdi-targetmiR482-3p_26
bdi-targetmiR482-3p_27
bdi-targetmiR482-3p_28
bdi-targetmiR482-3p_29
bdi-targetmiR482-3p_30
bdi-targetmiR482-3p_31
bdi-targetmiR482-3p_32
bdi-targetmiR482-3p_33
bdi-targetmiR482-3p_34
bdi-targetmiR482-3p_35
bdi-targetmiR482-3p_36
bdi-targetmiR482-3p_37
bdi-targetmiR482-3p_38
bdi-targetmiR482-3p_39
bdi-targetmiR482-3p_40
bdi-targetmiR482-3p_41
bdi-targetmiR482-3p_42
bdi-targetmiR482-3p_43
bdi-targetmiR482-3p_44
bdi-targetmiR482-3p_45
bdi-targetmiR482-3p_46
bdi-targetmiR482-3p_47
bdi-targetmiR482-3p_48
bdi-targetmiR482-3p_49
bdi-targetmiR482-3p_50
bdi-targetmiR482-3p_51
bdi-targetmiR482-3p_52
bdi-targetmiR482-3p_53
bdi-targetmiR482-3p_54
bdi-targetmiR482-3p_55
bdi-targetmiR482-3p_56
bdi-targetmiR482-3p_57
bdi-targetmiR482-3p_58
bdi-targetmiR482-3p_59
bdi-targetmiR482-3p_60
bdi-targetmiR482-3p_61
bdi-targetmiR482-3p_62
bdi-targetmiR482-3p_63
bdi-targetmiR482-3p_64
bdi-targetmiR482-3p_65
bdi-targetmiR482-3p_66
bdi-targetmiR482-3p_67
bdi-targetmiR482-3p_68
bdi-targetmiR482-3p_69
bdi-targetmiR482-3p_70
bdi-targetmiR482-3p_71
bdi-targetmiR482-3p_72
bdi-targetmiR482-3p_73
bdi-targetmiR482-3p_74
bdi-targetmiR482-3p_75
bdi-targetmiR482-3p_76
bdi-targetmiR482-3p_77
bdi-targetmiR482-3p_78
bdi-targetmiR482-3p_79

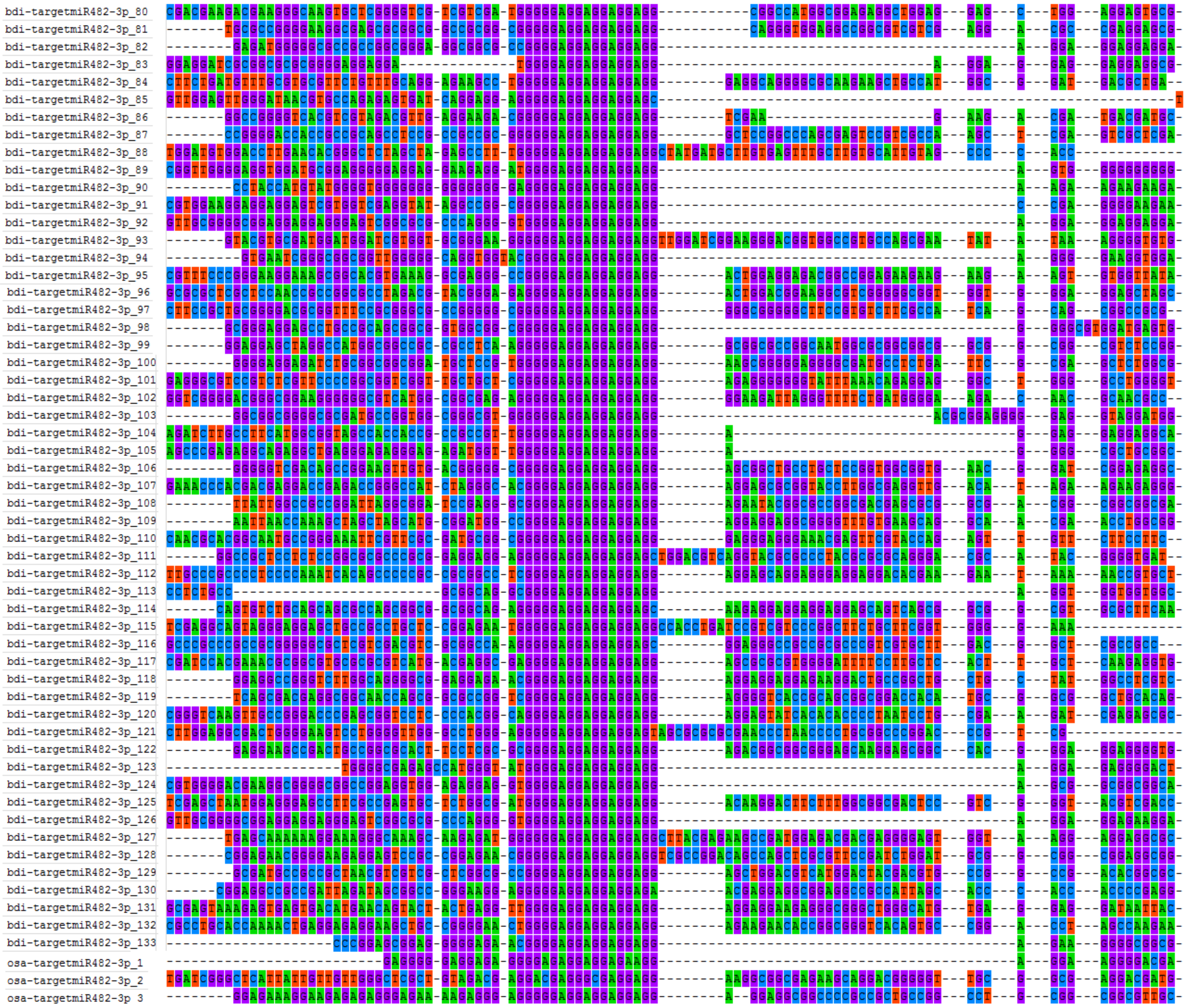
bdi-targetmiR482-3p_80
bdi-targetmiR482-3p_81
bdi-targetmiR482-3p_82
bdi-targetmiR482-3p_83
bdi-targetmiR482-3p_84
bdi-targetmiR482-3p_85
bdi-targetmiR482-3p_86
bdi-targetmiR482-3p_87
bdi-targetmiR482-3p_88
bdi-targetmiR482-3p_89
bdi-targetmiR482-3p_90
bdi-targetmiR482-3p_91
bdi-targetmiR482-3p_92
bdi-targetmiR482-3p_93
bdi-targetmiR482-3p_94
bdi-targetmiR482-3p_95
bdi-targetmiR482-3p_96
bdi-targetmiR482-3p_97
bdi-targetmiR482-3p_98
bdi-targetmiR482-3p_99
bdi-targetmiR482-3p_100
bdi-targetmiR482-3p_101
bdi-targetmiR482-3p_102
bdi-targetmiR482-3p_103
bdi-targetmiR482-3p_104
bdi-targetmiR482-3p_105
bdi-targetmiR482-3p_106
bdi-targetmiR482-3p_107
bdi-targetmiR482-3p_108
bdi-targetmiR482-3p_109
bdi-targetmiR482-3p_110
bdi-targetmiR482-3p_111
bdi-targetmiR482-3p_112
bdi-targetmiR482-3p_113
bdi-targetmiR482-3p_114
bdi-targetmiR482-3p_115
bdi-targetmiR482-3p_116
bdi-targetmiR482-3p_117
bdi-targetmiR482-3p_118
bdi-targetmiR482-3p_119
bdi-targetmiR482-3p_120
bdi-targetmiR482-3p_121
bdi-targetmiR482-3p_122
bdi-targetmiR482-3p_123
bdi-targetmiR482-3p_124
bdi-targetmiR482-3p_125
bdi-targetmiR482-3p_126
bdi-targetmiR482-3p_127
bdi-targetmiR482-3p_128
bdi-targetmiR482-3p_129
bdi-targetmiR482-3p_130
bdi-targetmiR482-3p_131
bdi-targetmiR482-3p_132
bdi-targetmiR482-3p_133
osa-targetmiR482-3p_1
osa-targetmiR482-3p_2
osa-targetmiR482-3p_3

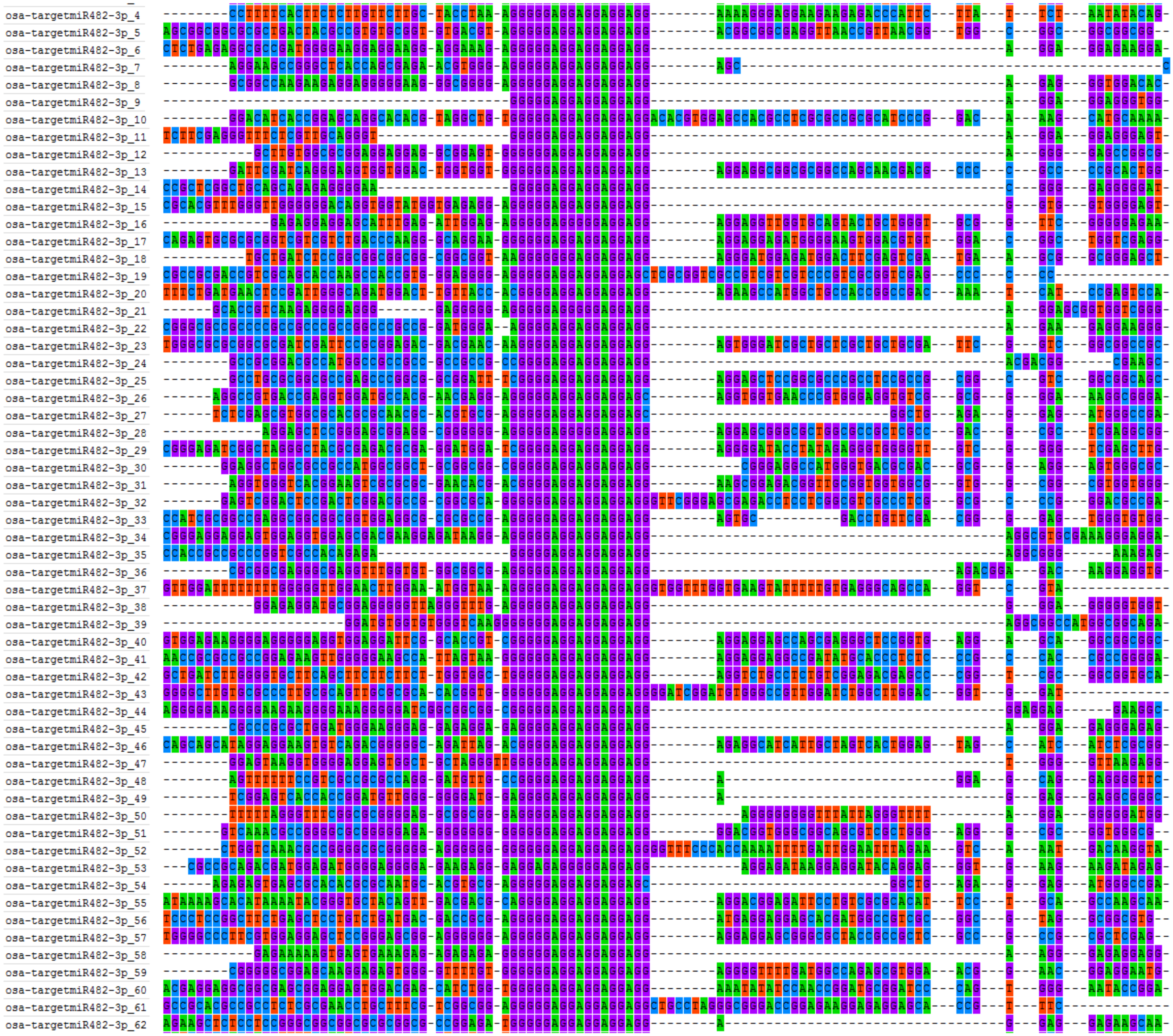
osa-targetmiR482-3p_4
osa-targetmiR482-3p_5
osa-targetmiR482-3p_6
osa-targetmiR482-3p_7
osa-targetmiR482-3p_8
osa-targetmiR482-3p_9
osa-targetmiR482-3p_10
osa-targetmiR482-3p_11
osa-targetmiR482-3p_12
osa-targetmiR482-3p_13
osa-targetmiR482-3p_14
osa-targetmiR482-3p_15
osa-targetmiR482-3p_16
osa-targetmiR482-3p_17
osa-targetmiR482-3p_18
osa-targetmiR482-3p_19
osa-targetmiR482-3p_20
osa-targetmiR482-3p_21
osa-targetmiR482-3p_22
osa-targetmiR482-3p_23
osa-targetmiR482-3p_24
osa-targetmiR482-3p_25
osa-targetmiR482-3p_26
osa-targetmiR482-3p_27
osa-targetmiR482-3p_28
osa-targetmiR482-3p_29
osa-targetmiR482-3p_30
osa-targetmiR482-3p_31
osa-targetmiR482-3p_32
osa-targetmiR482-3p_33
osa-targetmiR482-3p_34
osa-targetmiR482-3p_35
osa-targetmiR482-3p_36
osa-targetmiR482-3p_37
osa-targetmiR482-3p_38
osa-targetmiR482-3p_39
osa-targetmiR482-3p_40
osa-targetmiR482-3p_41
osa-targetmiR482-3p_42
osa-targetmiR482-3p_43
osa-targetmiR482-3p_44
osa-targetmiR482-3p_45
osa-targetmiR482-3p_46
osa-targetmiR482-3p_47
osa-targetmiR482-3p_48
osa-targetmiR482-3p_49
osa-targetmiR482-3p_50
osa-targetmiR482-3p_51
osa-targetmiR482-3p_52
osa-targetmiR482-3p_53
osa-targetmiR482-3p_54
osa-targetmiR482-3p_55
osa-targetmiR482-3p_56
osa-targetmiR482-3p_57
osa-targetmiR482-3p_58
osa-targetmiR482-3p_59
osa-targetmiR482-3p_60
osa-targetmiR482-3p_61
osa-targetmiR482-3p_62

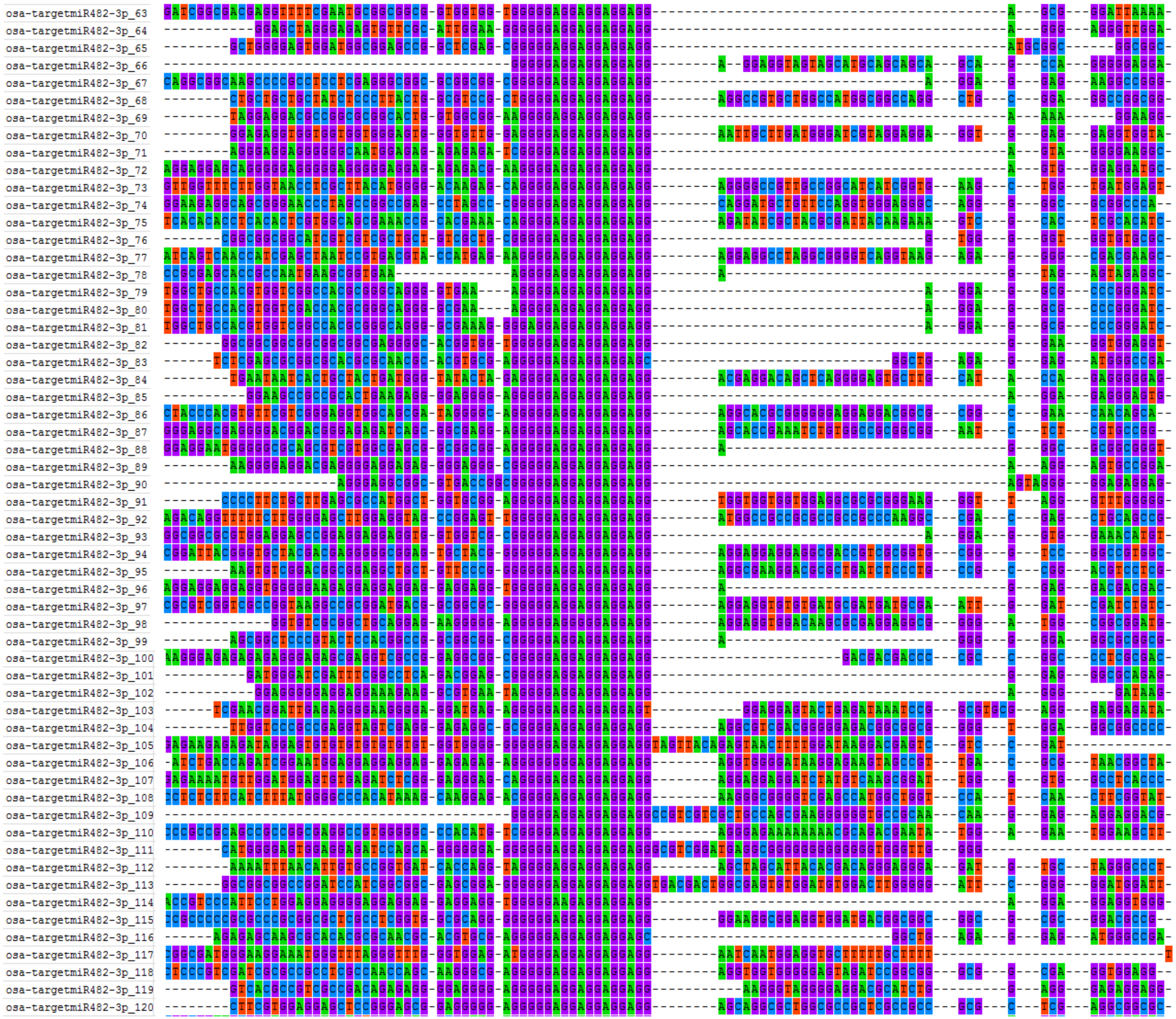
osa-targetmiR482-3p_63
osa-targetmiR482-3p_64
osa-targetmiR482-3p_65
osa-targetmiR482-3p_66
osa-targetmiR482-3p_67
osa-targetmiR482-3p_68
osa-targetmiR482-3p_69
osa-targetmiR482-3p_70
osa-targetmiR482-3p_71
osa-targetmiR482-3p_72
osa-targetmiR482-3p_73
osa-targetmiR482-3p_74
osa-targetmiR482-3p_75
osa-targetmiR482-3p_76
osa-targetmiR482-3p_77
osa-targetmiR482-3p_78
osa-targetmiR482-3p_79
osa-targetmiR482-3p_80
osa-targetmiR482-3p_81
osa-targetmiR482-3p_82
osa-targetmiR482-3p_83
osa-targetmiR482-3p_84
osa-targetmiR482-3p_85
osa-targetmiR482-3p_86
osa-targetmiR482-3p_87
osa-targetmiR482-3p_88
osa-targetmiR482-3p_89
osa-targetmiR482-3p_90
osa-targetmiR482-3p_91
osa-targetmiR482-3p_92
osa-targetmiR482-3p_93
osa-targetmiR482-3p_94
osa-targetmiR482-3p_95
osa-targetmiR482-3p_96
osa-targetmiR482-3p_97
osa-targetmiR482-3p_98
osa-targetmiR482-3p_99
osa-targetmiR482-3p_100
osa-targetmiR482-3p_101
osa-targetmiR482-3p_102
osa-targetmiR482-3p_103
osa-targetmiR482-3p_104
osa-targetmiR482-3p_105
osa-targetmiR482-3p_106
osa-targetmiR482-3p_107
osa-targetmiR482-3p_108
osa-targetmiR482-3p_109
osa-targetmiR482-3p_110
osa-targetmiR482-3p_111
osa-targetmiR482-3p_112
osa-targetmiR482-3p_113
osa-targetmiR482-3p_114
osa-targetmiR482-3p_115
osa-targetmiR482-3p_116
osa-targetmiR482-3p_117
osa-targetmiR482-3p_118
osa-targetmiR482-3p_119
osa-targetmiR482-3p_120

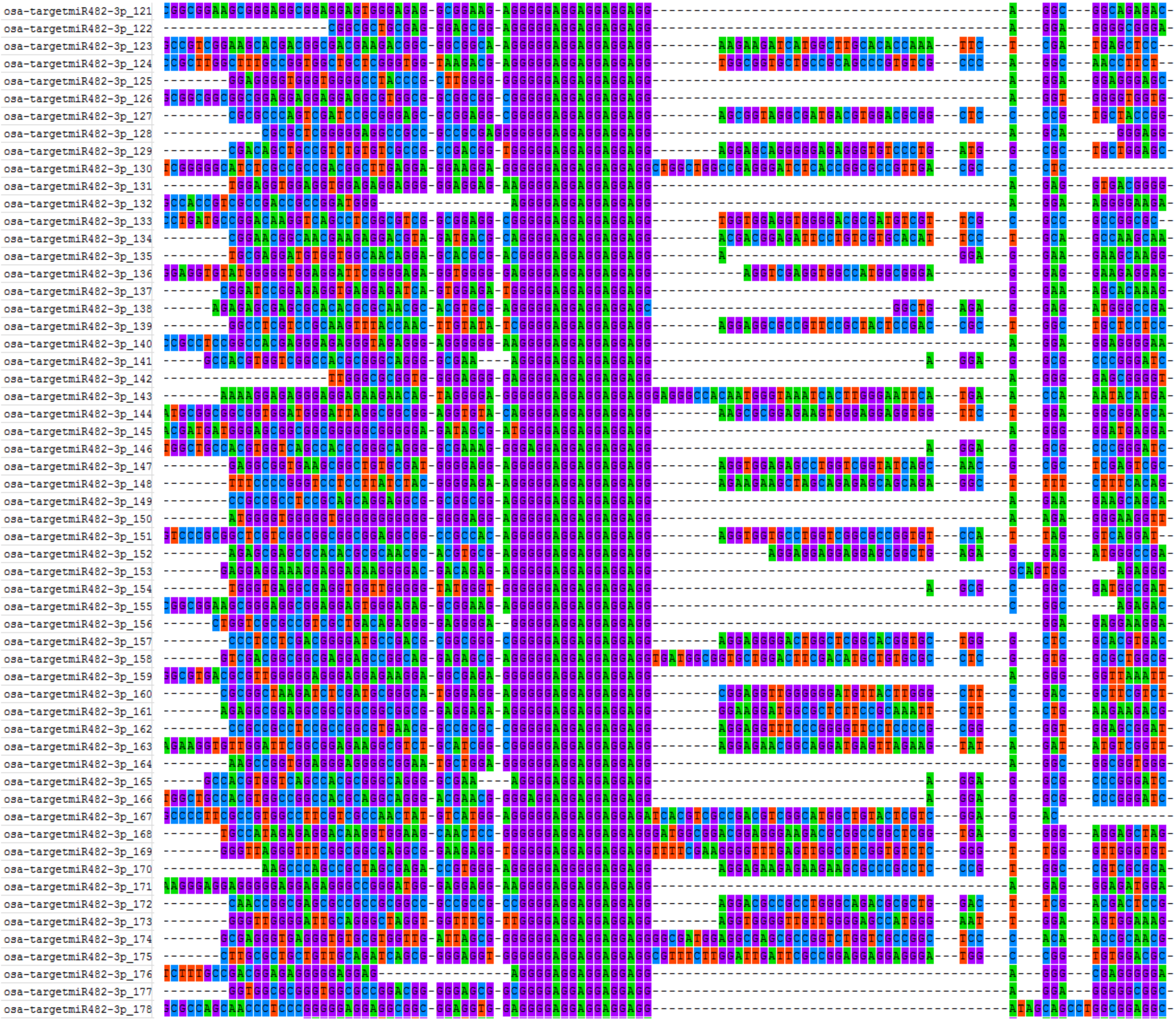

osa-targetmiR482-3p_121
osa-targetmiR482-3p_122
osa-targetmiR482-3p_123
osa-targetmiR482-3p_124
osa-targetmiR482-3p_125
osa-targetmiR482-3p_126
osa-targetmiR482-3p_127
osa-targetmiR482-3p_128
osa-targetmiR482-3p_129
osa-targetmiR482-3p_130
osa-targetmiR482-3p_131
osa-targetmiR482-3p_132
osa-targetmiR482-3p_133
osa-targetmiR482-3p_134
osa-targetmiR482-3p_135
osa-targetmiR482-3p_136
osa-targetmiR482-3p_137
osa-targetmiR482-3p_138
osa-targetmiR482-3p_139
osa-targetmiR482-3p_140
osa-targetmiR482-3p_141
osa-targetmiR482-3p_142
osa-targetmiR482-3p_143
osa-targetmiR482-3p_144
osa-targetmiR482-3p_145
osa-targetmiR482-3p_146
osa-targetmiR482-3p_147
osa-targetmiR482-3p_148
osa-targetmiR482-3p_149
osa-targetmiR482-3p_150
osa-targetmiR482-3p_151
osa-targetmiR482-3p_152
osa-targetmiR482-3p_153
osa-targetmiR482-3p_154
osa-targetmiR482-3p_155
osa-targetmiR482-3p_156
osa-targetmiR482-3p_157
osa-targetmiR482-3p_158
osa-targetmiR482-3p_159
osa-targetmiR482-3p_160
osa-targetmiR482-3p_161
osa-targetmiR482-3p_162
osa-targetmiR482-3p_163
osa-targetmiR482-3p_164
osa-targetmiR482-3p_165
osa-targetmiR482-3p_166
osa-targetmiR482-3p_167
osa-targetmiR482-3p_168
osa-targetmiR482-3p_169
osa-targetmiR482-3p_170
osa-targetmiR482-3p_171
osa-targetmiR482-3p_172
osa-targetmiR482-3p_173
osa-targetmiR482-3p_174
osa-targetmiR482-3p_175
osa-targetmiR482-3p_176
osa-targetmiR482-3p_177
osa-targetmiR482-3p_178

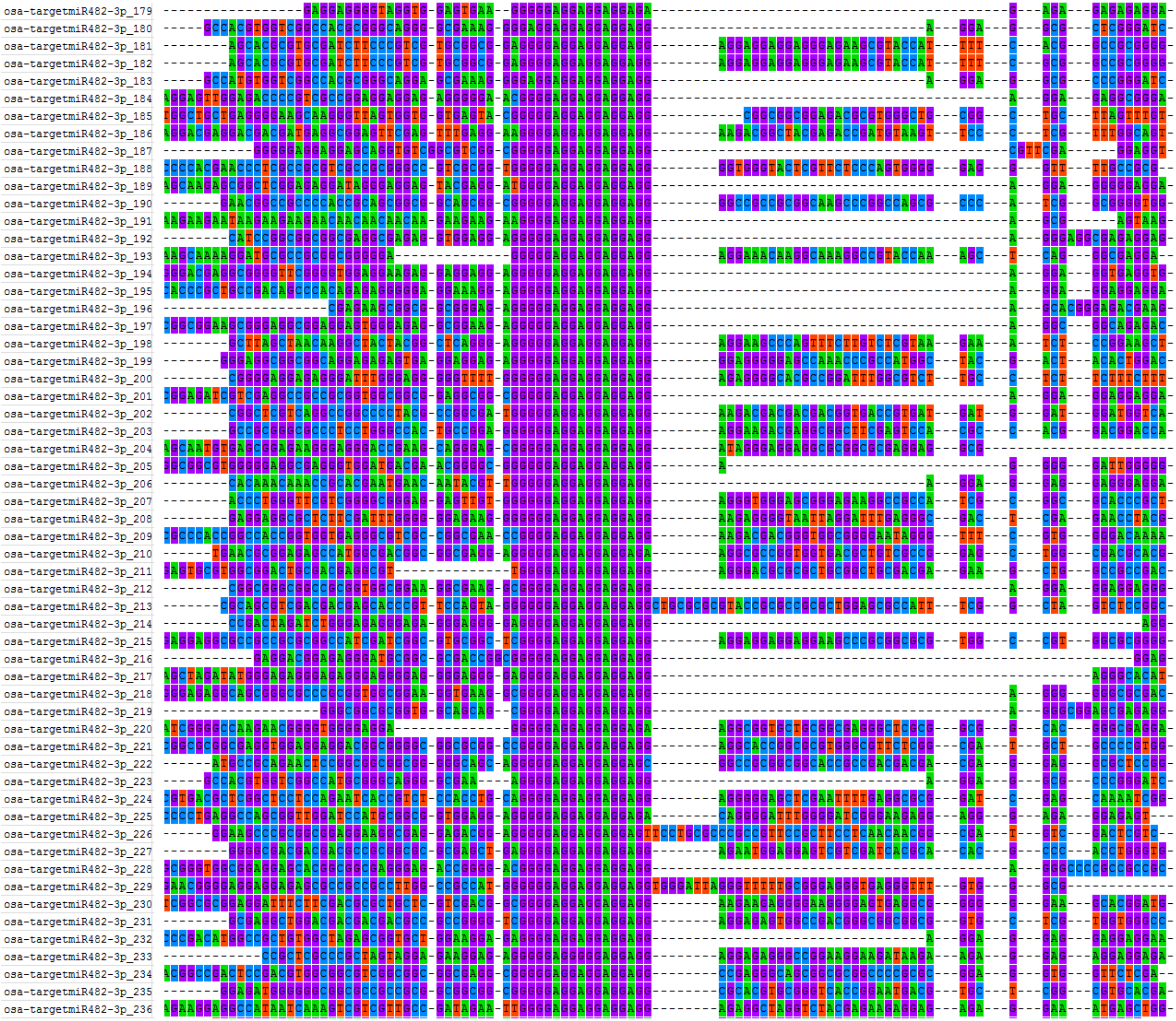
osa-targetmiR482-3p_179
osa-targetmiR482-3p_180
osa-targetmiR482-3p_181
osa-targetmiR482-3p_182
osa-targetmiR482-3p_183
osa-targetmiR482-3p_184
osa-targetmiR482-3p_185
osa-targetmiR482-3p_186
osa-targetmiR482-3p_187
osa-targetmiR482-3p_188
osa-targetmiR482-3p_189
osa-targetmiR482-3p_190
osa-targetmiR482-3p_191
osa-targetmiR482-3p_192
osa-targetmiR482-3p_193
osa-targetmiR482-3p_194
osa-targetmiR482-3p_195
osa-targetmiR482-3p_196
osa-targetmiR482-3p_197
osa-targetmiR482-3p_198
osa-targetmiR482-3p_199
osa-targetmiR482-3p_200
osa-targetmiR482-3p_201
osa-targetmiR482-3p_202
osa-targetmiR482-3p_203
osa-targetmiR482-3p_204
osa-targetmiR482-3p_205
osa-targetmiR482-3p_206
osa-targetmiR482-3p_207
osa-targetmiR482-3p_208
osa-targetmiR482-3p_209
osa-targetmiR482-3p_210
osa-targetmiR482-3p_211
osa-targetmiR482-3p_212
osa-targetmiR482-3p_213
osa-targetmiR482-3p_214
osa-targetmiR482-3p_215
osa-targetmiR482-3p_216
osa-targetmiR482-3p_217
osa-targetmiR482-3p_218
osa-targetmiR482-3p_219
osa-targetmiR482-3p_220
osa-targetmiR482-3p_221
osa-targetmiR482-3p_222
osa-targetmiR482-3p_223
osa-targetmiR482-3p_224
osa-targetmiR482-3p_225
osa-targetmiR482-3p_226
osa-targetmiR482-3p_227
osa-targetmiR482-3p_228
osa-targetmiR482-3p_229
osa-targetmiR482-3p_230
osa-targetmiR482-3p_231
osa-targetmiR482-3p_232
osa-targetmiR482-3p_233
osa-targetmiR482-3p_234
osa-targetmiR482-3p_235
osa-targetmiR482-3p_236

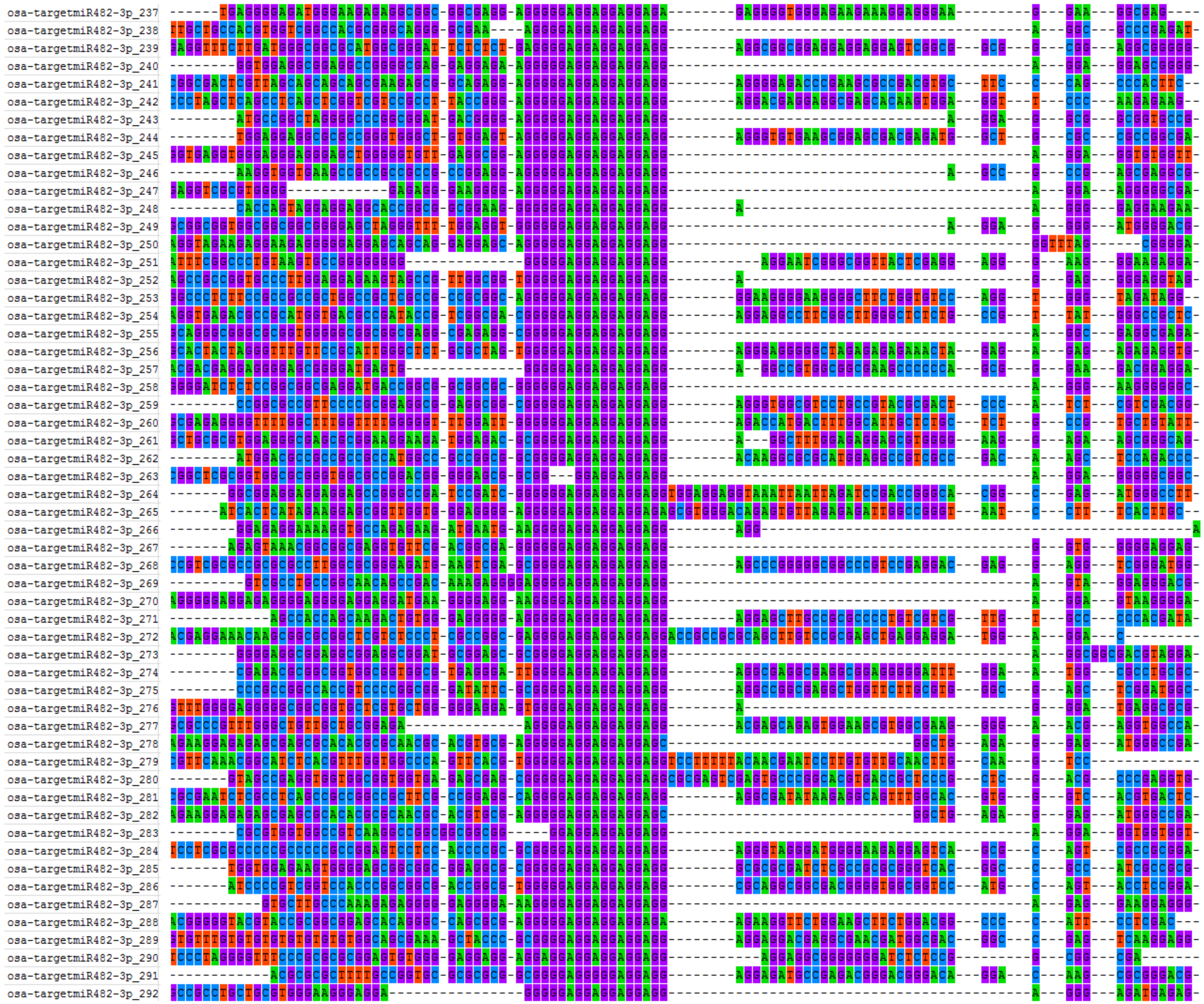
osa-targetmiR482-3p_237
osa-targetmiR482-3p_238
osa-targetmiR482-3p_239
osa-targetmiR482-3p_240
osa-targetmiR482-3p_241
osa-targetmiR482-3p_242
osa-targetmiR482-3p_243
osa-targetmiR482-3p_244
osa-targetmiR482-3p_245
osa-targetmiR482-3p_246
osa-targetmiR482-3p_247
osa-targetmiR482-3p_248
osa-targetmiR482-3p_249
osa-targetmiR482-3p_250
osa-targetmiR482-3p_251
osa-targetmiR482-3p_252
osa-targetmiR482-3p_253
osa-targetmiR482-3p_254
osa-targetmiR482-3p_255
osa-targetmiR482-3p_256
osa-targetmiR482-3p_257
osa-targetmiR482-3p_258
osa-targetmiR482-3p_259
osa-targetmiR482-3p_260
osa-targetmiR482-3p_261
osa-targetmiR482-3p_262
osa-targetmiR482-3p_263
osa-targetmiR482-3p_264
osa-targetmiR482-3p_265
osa-targetmiR482-3p_266
osa-targetmiR482-3p_267
osa-targetmiR482-3p_268
osa-targetmiR482-3p_269
osa-targetmiR482-3p_270
osa-targetmiR482-3p_271
osa-targetmiR482-3p_272
osa-targetmiR482-3p_273
osa-targetmiR482-3p_274
osa-targetmiR482-3p_275
osa-targetmiR482-3p_276
osa-targetmiR482-3p_277
osa-targetmiR482-3p_278
osa-targetmiR482-3p_279
osa-targetmiR482-3p_280
osa-targetmiR482-3p_281
osa-targetmiR482-3p_282
osa-targetmiR482-3p_283
osa-targetmiR482-3p_284
osa-targetmiR482-3p_285
osa-targetmiR482-3p_286
osa-targetmiR482-3p_287
osa-targetmiR482-3p_288
osa-targetmiR482-3p_289
osa-targetmiR482-3p_290
osa-targetmiR482-3p_291
osa-targetmiR482-3p_292

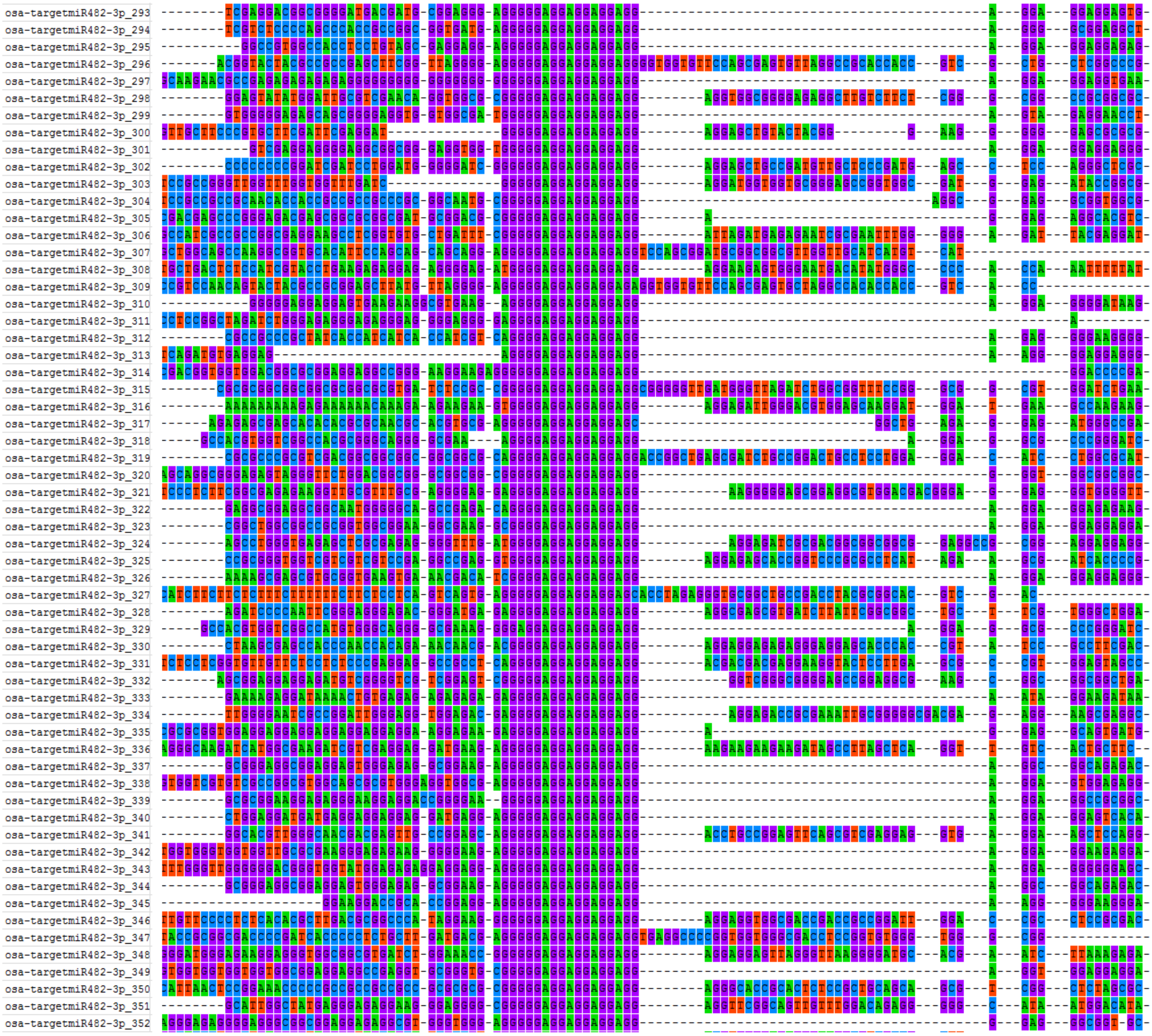
osa-targetmiR482-3p_293
osa-targetmiR482-3p_294
osa-targetmiR482-3p_295
osa-targetmiR482-3p_296
osa-targetmiR482-3p_297
osa-targetmiR482-3p_298
osa-targetmiR482-3p_299
osa-targetmiR482-3p_300
osa-targetmiR482-3p_301
osa-targetmiR482-3p_302
osa-targetmiR482-3p_303
osa-targetmiR482-3p_304
osa-targetmiR482-3p_305
osa-targetmiR482-3p_306
osa-targetmiR482-3p_307
osa-targetmiR482-3p_308
osa-targetmiR482-3p_309
osa-targetmiR482-3p_310
osa-targetmiR482-3p_311
osa-targetmiR482-3p_312
osa-targetmiR482-3p_313
osa-targetmiR482-3p_314
osa-targetmiR482-3p_315
osa-targetmiR482-3p_316
osa-targetmiR482-3p_317
osa-targetmiR482-3p_318
osa-targetmiR482-3p_319
osa-targetmiR482-3p_320
osa-targetmiR482-3p_321
osa-targetmiR482-3p_322
osa-targetmiR482-3p_323
osa-targetmiR482-3p_324
osa-targetmiR482-3p_325
osa-targetmiR482-3p_326
osa-targetmiR482-3p_327
osa-targetmiR482-3p_328
osa-targetmiR482-3p_329
osa-targetmiR482-3p_330
osa-targetmiR482-3p_331
osa-targetmiR482-3p_332
osa-targetmiR482-3p_333
osa-targetmiR482-3p_334
osa-targetmiR482-3p_335
osa-targetmiR482-3p_336
osa-targetmiR482-3p_337
osa-targetmiR482-3p_338
osa-targetmiR482-3p_339
osa-targetmiR482-3p_340
osa-targetmiR482-3p_341
osa-targetmiR482-3p_342
osa-targetmiR482-3p_343
osa-targetmiR482-3p_344
osa-targetmiR482-3p_345
osa-targetmiR482-3p_346
osa-targetmiR482-3p_347
osa-targetmiR482-3p_348
osa-targetmiR482-3p_349
osa-targetmiR482-3p_350
osa-targetmiR482-3p_351
osa-targetmiR482-3p_352

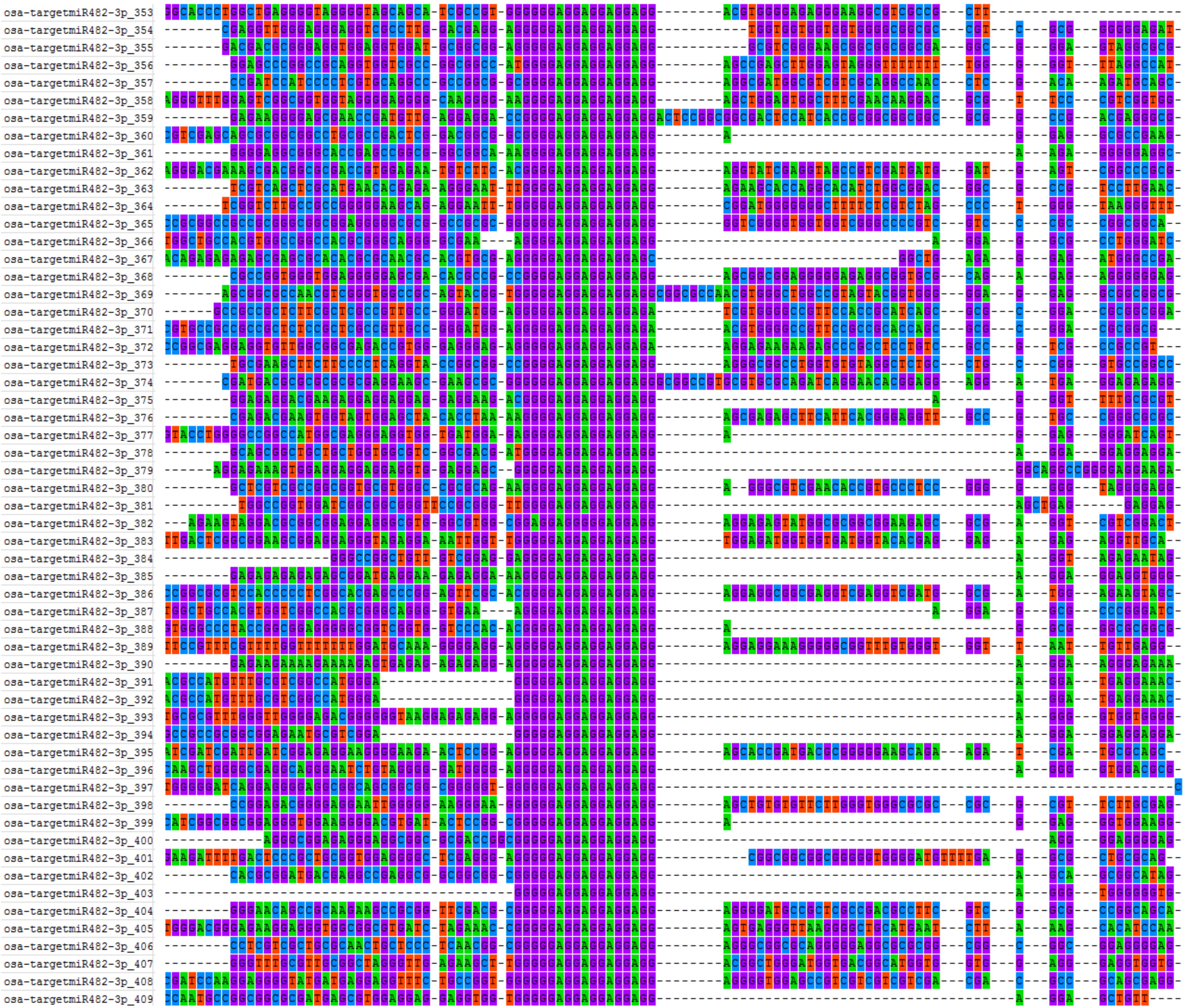
osa-targetmiR482-3p_353
osa-targetmiR482-3p_354
osa-targetmiR482-3p_355
osa-targetmiR482-3p_356
osa-targetmiR482-3p_357
osa-targetmiR482-3p_358
osa-targetmiR482-3p_359
osa-targetmiR482-3p_360
osa-targetmiR482-3p_361
osa-targetmiR482-3p_362
osa-targetmiR482-3p_363
osa-targetmiR482-3p_364
osa-targetmiR482-3p_365
osa-targetmiR482-3p_366
osa-targetmiR482-3p_367
osa-targetmiR482-3p_368
osa-targetmiR482-3p_369
osa-targetmiR482-3p_370
osa-targetmiR482-3p_371
osa-targetmiR482-3p_372
osa-targetmiR482-3p_373
osa-targetmiR482-3p_374
osa-targetmiR482-3p_375
osa-targetmiR482-3p_376
osa-targetmiR482-3p_377
osa-targetmiR482-3p_378
osa-targetmiR482-3p_379
osa-targetmiR482-3p_380
osa-targetmiR482-3p_381
osa-targetmiR482-3p_382
osa-targetmiR482-3p_383
osa-targetmiR482-3p_384
osa-targetmiR482-3p_385
osa-targetmiR482-3p_386
osa-targetmiR482-3p_387
osa-targetmiR482-3p_388
osa-targetmiR482-3p_389
osa-targetmiR482-3p_390
osa-targetmiR482-3p_391
osa-targetmiR482-3p_392
osa-targetmiR482-3p_393
osa-targetmiR482-3p_394
osa-targetmiR482-3p_395
osa-targetmiR482-3p_396
osa-targetmiR482-3p_397
osa-targetmiR482-3p_398
osa-targetmiR482-3p_399
osa-targetmiR482-3p_400
osa-targetmiR482-3p_401
osa-targetmiR482-3p_402
osa-targetmiR482-3p_403
osa-targetmiR482-3p_404
osa-targetmiR482-3p_405
osa-targetmiR482-3p_406
osa-targetmiR482-3p_407
osa-targetmiR482-3p_408
osa-targetmiR482-3p_409

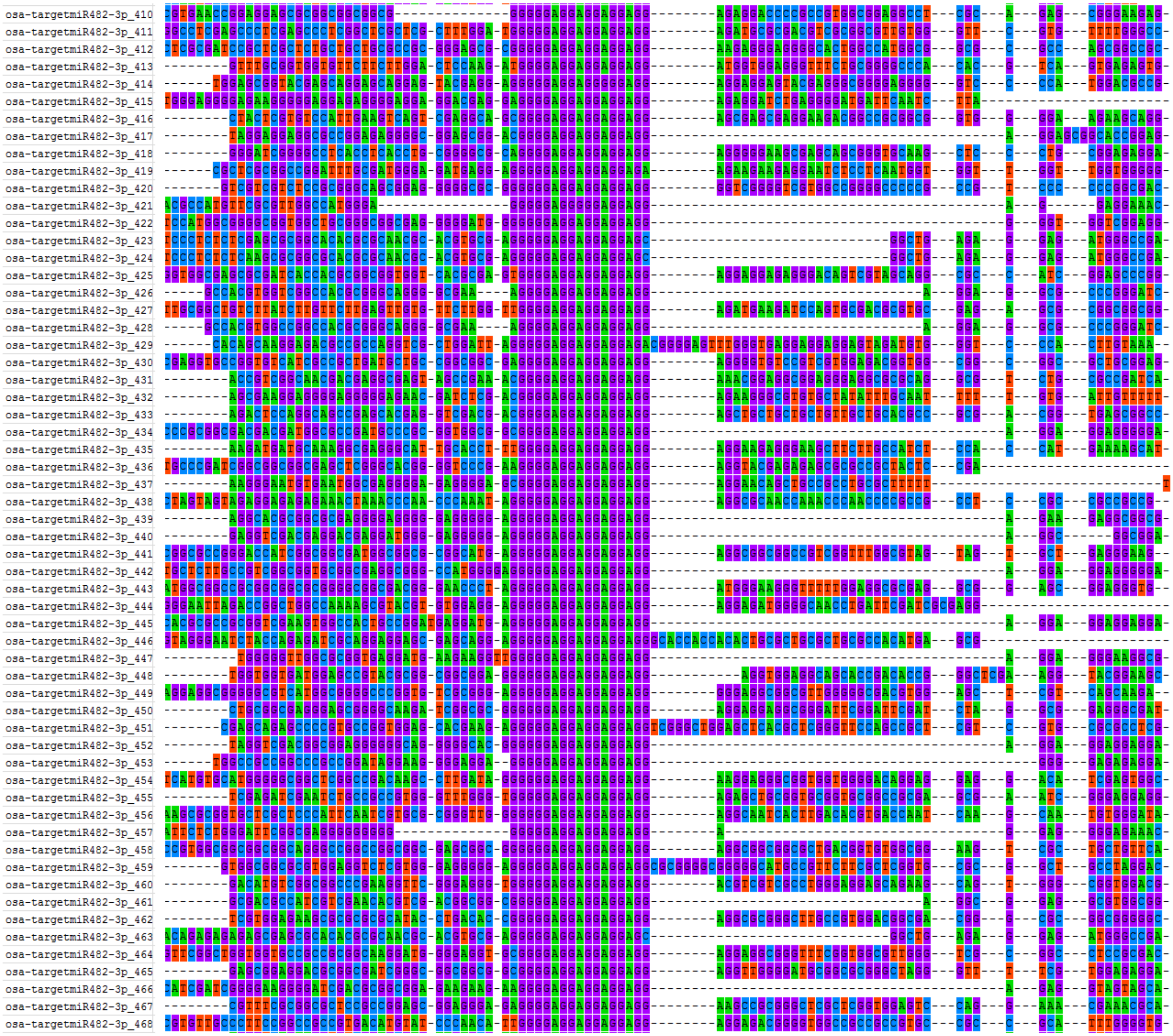
osa-targetmiR482-3p_410
osa-targetmiR482-3p_411
osa-targetmiR482-3p_412
osa-targetmiR482-3p_413
osa-targetmiR482-3p_414
osa-targetmiR482-3p_415
osa-targetmiR482-3p_416
osa-targetmiR482-3p_417
osa-targetmiR482-3p_418
osa-targetmiR482-3p_419
osa-targetmiR482-3p_420
osa-targetmiR482-3p_421
osa-targetmiR482-3p_422
osa-targetmiR482-3p_423
osa-targetmiR482-3p_424
osa-targetmiR482-3p_425
osa-targetmiR482-3p_426
osa-targetmiR482-3p_427
osa-targetmiR482-3p_428
osa-targetmiR482-3p_429
osa-targetmiR482-3p_430
osa-targetmiR482-3p_431
osa-targetmiR482-3p_432
osa-targetmiR482-3p_433
osa-targetmiR482-3p_434
osa-targetmiR482-3p_435
osa-targetmiR482-3p_436
osa-targetmiR482-3p_437
osa-targetmiR482-3p_438
osa-targetmiR482-3p_439
osa-targetmiR482-3p_440
osa-targetmiR482-3p_441
osa-targetmiR482-3p_442
osa-targetmiR482-3p_443
osa-targetmiR482-3p_444
osa-targetmiR482-3p_445
osa-targetmiR482-3p_446
osa-targetmiR482-3p_447
osa-targetmiR482-3p_448
osa-targetmiR482-3p_449
osa-targetmiR482-3p_450
osa-targetmiR482-3p_451
osa-targetmiR482-3p_452
osa-targetmiR482-3p_453
osa-targetmiR482-3p_454
osa-targetmiR482-3p_455
osa-targetmiR482-3p_456
osa-targetmiR482-3p_457
osa-targetmiR482-3p_458
osa-targetmiR482-3p_459
osa-targetmiR482-3p_460
osa-targetmiR482-3p_461
osa-targetmiR482-3p_462
osa-targetmiR482-3p_463
osa-targetmiR482-3p_464
osa-targetmiR482-3p_465
osa-targetmiR482-3p_466
osa-targetmiR482-3p_467
osa-targetmiR482-3p_468

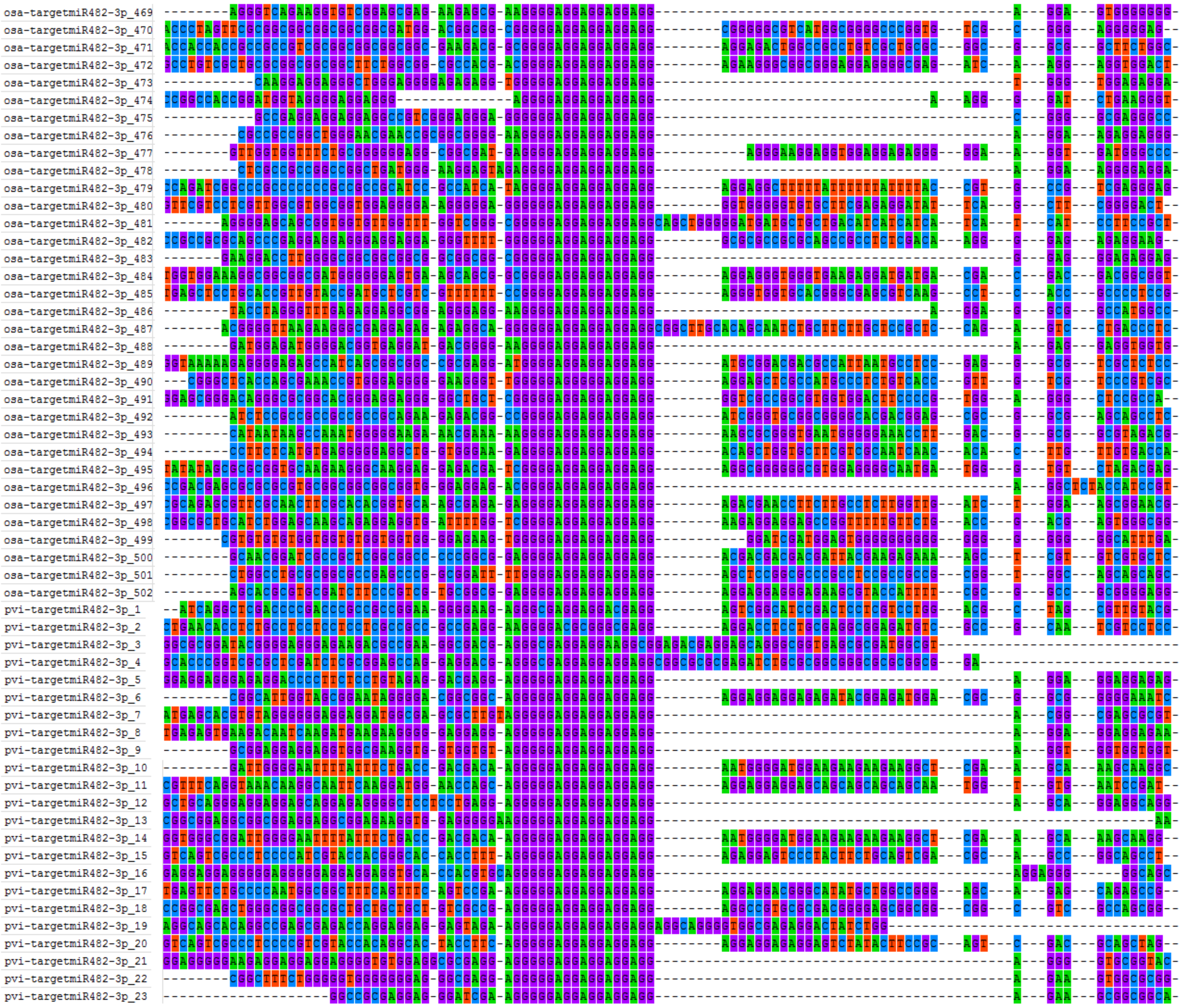
osa-targetmiR482-3p_469
osa-targetmiR482-3p_470
osa-targetmiR482-3p_471
osa-targetmiR482-3p_472
osa-targetmiR482-3p_473
osa-targetmiR482-3p_474
osa-targetmiR482-3p_475
osa-targetmiR482-3p_476
osa-targetmiR482-3p_477
osa-targetmiR482-3p_478
osa-targetmiR482-3p_479
osa-targetmiR482-3p_480
osa-targetmiR482-3p_481
osa-targetmiR482-3p_482
osa-targetmiR482-3p_483
osa-targetmiR482-3p_484
osa-targetmiR482-3p_485
osa-targetmiR482-3p_486
osa-targetmiR482-3p_487
osa-targetmiR482-3p_488
osa-targetmiR482-3p_489
osa-targetmiR482-3p_490
osa-targetmiR482-3p_491
osa-targetmiR482-3p_492
osa-targetmiR482-3p_493
osa-targetmiR482-3p_494
osa-targetmiR482-3p_495
osa-targetmiR482-3p_496
osa-targetmiR482-3p_497
osa-targetmiR482-3p_498
osa-targetmiR482-3p_499
osa-targetmiR482-3p_500
osa-targetmiR482-3p_501
osa-targetmiR482-3p_502
pvi-targetmiR482-3p_1
pvi-targetmiR482-3p_2
pvi-targetmiR482-3p_3
pvi-targetmiR482-3p_4
pvi-targetmiR482-3p_5
pvi-targetmiR482-3p_6
pvi-targetmiR482-3p_7
pvi-targetmiR482-3p_8
pvi-targetmiR482-3p_9
pvi-targetmiR482-3p_10
pvi-targetmiR482-3p_11
pvi-targetmiR482-3p_12
pvi-targetmiR482-3p_13
pvi-targetmiR482-3p_14
pvi-targetmiR482-3p_15
pvi-targetmiR482-3p_16
pvi-targetmiR482-3p_17
pvi-targetmiR482-3p_18
pvi-targetmiR482-3p_19
pvi-targetmiR482-3p_20
pvi-targetmiR482-3p_21
pvi-targetmiR482-3p_22
pvi-targetmiR482-3p_23

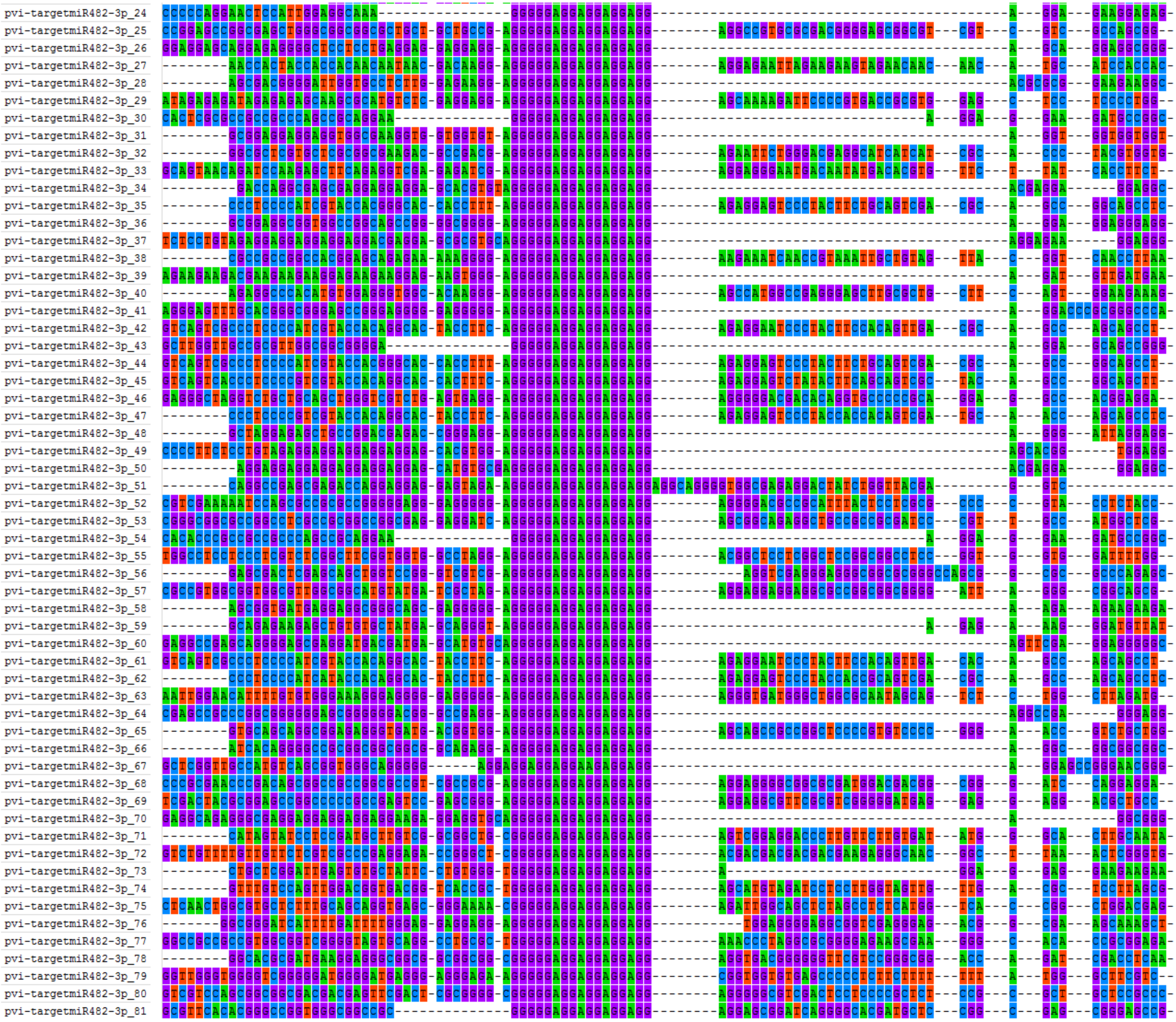
pvi-targetmiR482-3p_24
pvi-targetmiR482-3p_25
pvi-targetmiR482-3p_26
pvi-targetmiR482-3p_27
pvi-targetmiR482-3p_28
pvi-targetmiR482-3p_29
pvi-targetmiR482-3p_30
pvi-targetmiR482-3p_31
pvi-targetmiR482-3p_32
pvi-targetmiR482-3p_33
pvi-targetmiR482-3p_34
pvi-targetmiR482-3p_35
pvi-targetmiR482-3p_36
pvi-targetmiR482-3p_37
pvi-targetmiR482-3p_38
pvi-targetmiR482-3p_39
pvi-targetmiR482-3p_40
pvi-targetmiR482-3p_41
pvi-targetmiR482-3p_42
pvi-targetmiR482-3p_43
pvi-targetmiR482-3p_44
pvi-targetmiR482-3p_45
pvi-targetmiR482-3p_46
pvi-targetmiR482-3p_47
pvi-targetmiR482-3p_48
pvi-targetmiR482-3p_49
pvi-targetmiR482-3p_50
pvi-targetmiR482-3p_51
pvi-targetmiR482-3p_52
pvi-targetmiR482-3p_53
pvi-targetmiR482-3p_54
pvi-targetmiR482-3p_55
pvi-targetmiR482-3p_56
pvi-targetmiR482-3p_57
pvi-targetmiR482-3p_58
pvi-targetmiR482-3p_59
pvi-targetmiR482-3p_60
pvi-targetmiR482-3p_61
pvi-targetmiR482-3p_62
pvi-targetmiR482-3p_63
pvi-targetmiR482-3p_64
pvi-targetmiR482-3p_65
pvi-targetmiR482-3p_66
pvi-targetmiR482-3p_67
pvi-targetmiR482-3p_68
pvi-targetmiR482-3p_69
pvi-targetmiR482-3p_70
pvi-targetmiR482-3p_71
pvi-targetmiR482-3p_72
pvi-targetmiR482-3p_73
pvi-targetmiR482-3p_74
pvi-targetmiR482-3p_75
pvi-targetmiR482-3p_76
pvi-targetmiR482-3p_77
pvi-targetmiR482-3p_78
pvi-targetmiR482-3p_79
pvi-targetmiR482-3p_80
pvi-targetmiR482-3p_81

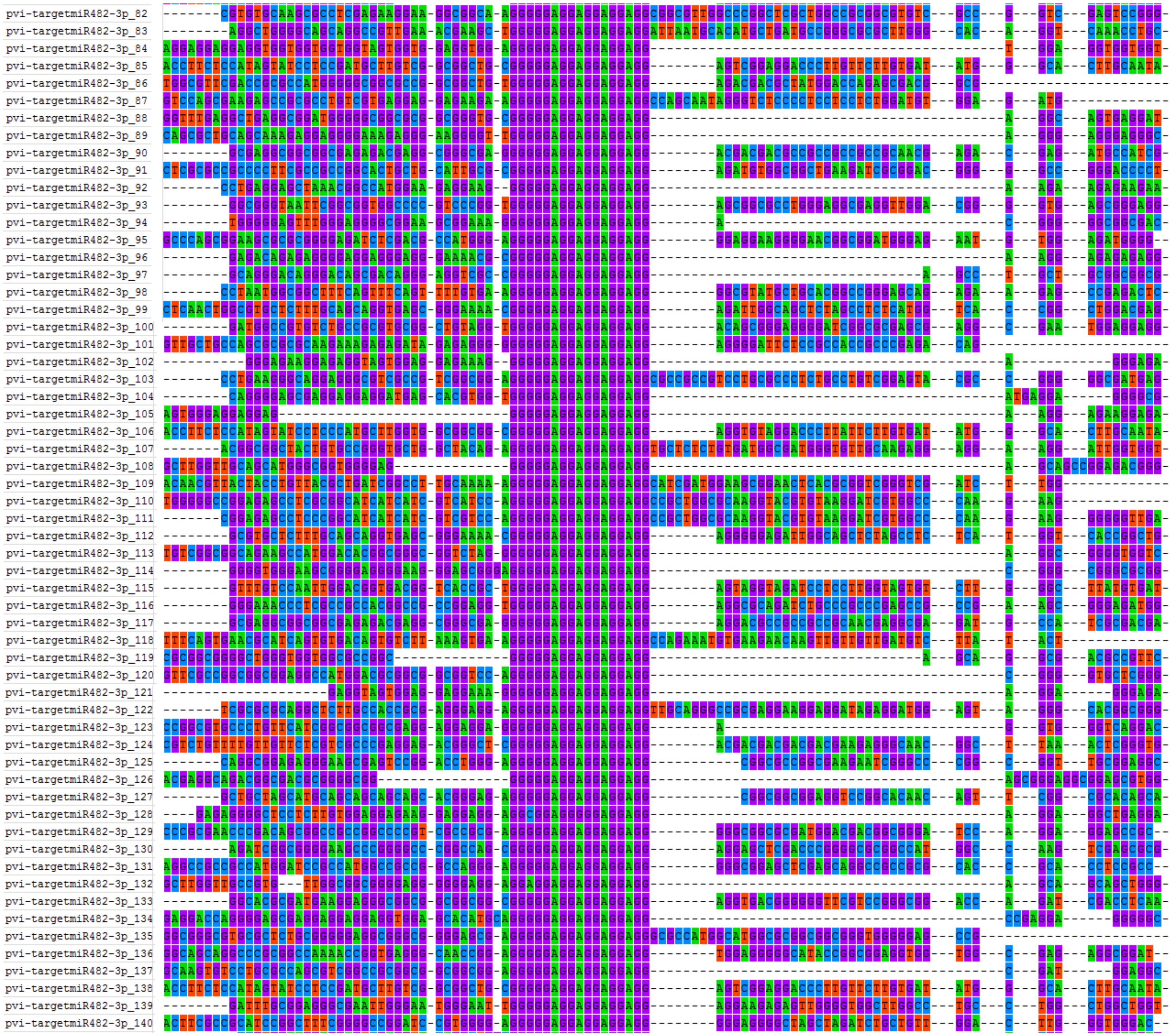

pvi-targetmiR482-3p_82
pvi-targetmiR482-3p_83
pvi-targetmiR482-3p_84
pvi-targetmiR482-3p_85
pvi-targetmiR482-3p_86
pvi-targetmiR482-3p_87
pvi-targetmiR482-3p_88
pvi-targetmiR482-3p_89
pvi-targetmiR482-3p_90
pvi-targetmiR482-3p_91
pvi-targetmiR482-3p_92
pvi-targetmiR482-3p_93
pvi-targetmiR482-3p_94
pvi-targetmiR482-3p_95
pvi-targetmiR482-3p_96
pvi-targetmiR482-3p_97
pvi-targetmiR482-3p_98
pvi-targetmiR482-3p_99
pvi-targetmiR482-3p_100
pvi-targetmiR482-3p_101
pvi-targetmiR482-3p_102
pvi-targetmiR482-3p_103
pvi-targetmiR482-3p_104
pvi-targetmiR482-3p_105
pvi-targetmiR482-3p_106
pvi-targetmiR482-3p_107
pvi-targetmiR482-3p_108
pvi-targetmiR482-3p_109
pvi-targetmiR482-3p_110
pvi-targetmiR482-3p_111
pvi-targetmiR482-3p_112
pvi-targetmiR482-3p_113
pvi-targetmiR482-3p_114
pvi-targetmiR482-3p_115
pvi-targetmiR482-3p_116
pvi-targetmiR482-3p_117
pvi-targetmiR482-3p_118
pvi-targetmiR482-3p_119
pvi-targetmiR482-3p_120
pvi-targetmiR482-3p_121
pvi-targetmiR482-3p_122
pvi-targetmiR482-3p_123
pvi-targetmiR482-3p_124
pvi-targetmiR482-3p_125
pvi-targetmiR482-3p_126
pvi-targetmiR482-3p_127
pvi-targetmiR482-3p_128
pvi-targetmiR482-3p_129
pvi-targetmiR482-3p_130
pvi-targetmiR482-3p_131
pvi-targetmiR482-3p_132
pvi-targetmiR482-3p_133
pvi-targetmiR482-3p_134
pvi-targetmiR482-3p_135
pvi-targetmiR482-3p_136
pvi-targetmiR482-3p_137
pvi-targetmiR482-3p_138
pvi-targetmiR482-3p_139
pvi-targetmiR482-3p_140

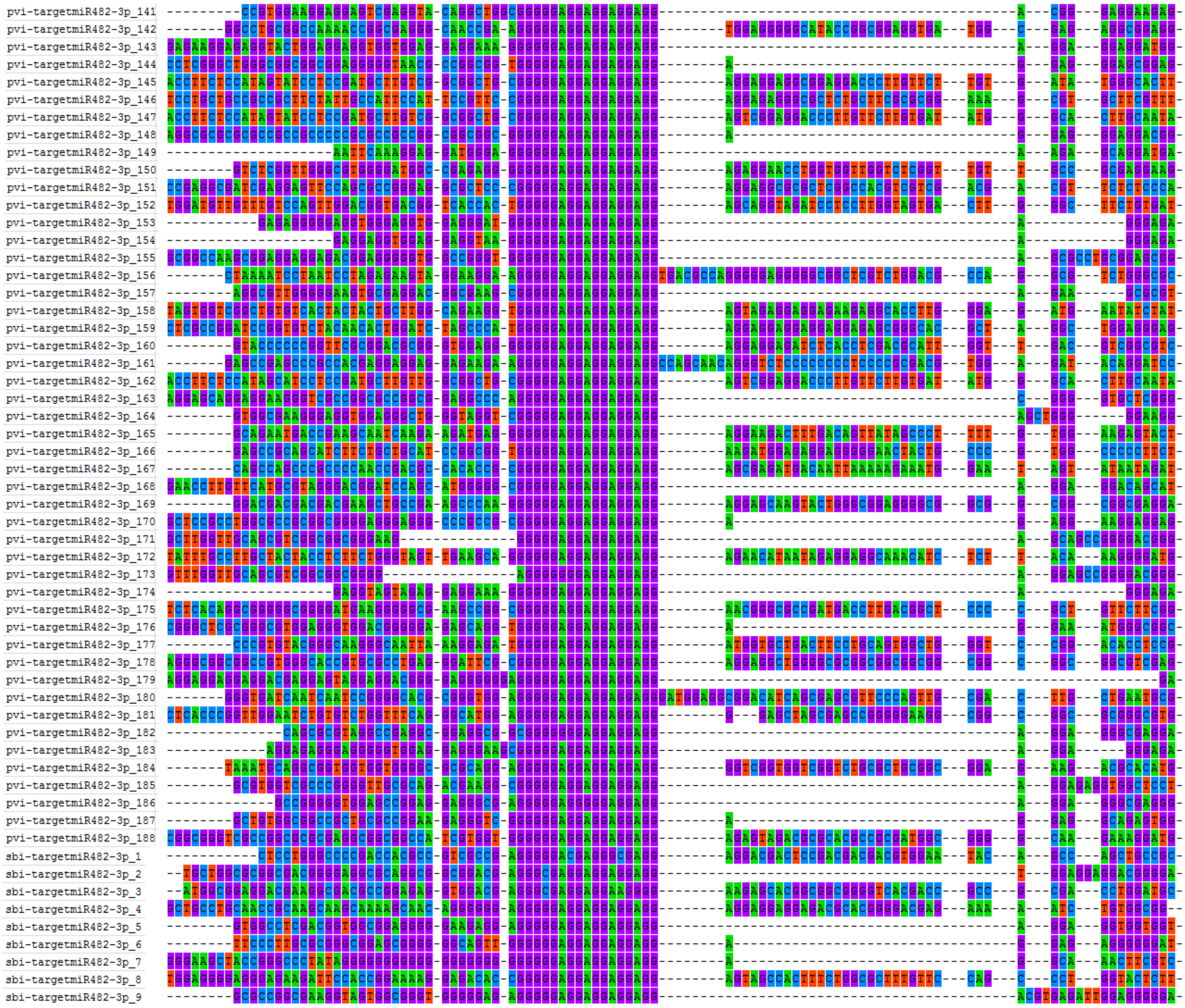
pvi-targetmiR482-3p_141
pvi-targetmiR482-3p_142
pvi-targetmiR482-3p_143
pvi-targetmiR482-3p_144
pvi-targetmiR482-3p_145
pvi-targetmiR482-3p_146
pvi-targetmiR482-3p_147
pvi-targetmiR482-3p_148
pvi-targetmiR482-3p_149
pvi-targetmiR482-3p_150
pvi-targetmiR482-3p_151
pvi-targetmiR482-3p_152
pvi-targetmiR482-3p_153
pvi-targetmiR482-3p_154
pvi-targetmiR482-3p_155
pvi-targetmiR482-3p_156
pvi-targetmiR482-3p_157
pvi-targetmiR482-3p_158
pvi-targetmiR482-3p_159
pvi-targetmiR482-3p_160
pvi-targetmiR482-3p_161
pvi-targetmiR482-3p_162
pvi-targetmiR482-3p_163
pvi-targetmiR482-3p_164
pvi-targetmiR482-3p_165
pvi-targetmiR482-3p_166
pvi-targetmiR482-3p_167
pvi-targetmiR482-3p_168
pvi-targetmiR482-3p_169
pvi-targetmiR482-3p_170
pvi-targetmiR482-3p_171
pvi-targetmiR482-3p_172
pvi-targetmiR482-3p_173
pvi-targetmiR482-3p_174
pvi-targetmiR482-3p_175
pvi-targetmiR482-3p_176
pvi-targetmiR482-3p_177
pvi-targetmiR482-3p_178
pvi-targetmiR482-3p_179
pvi-targetmiR482-3p_180
pvi-targetmiR482-3p_181
pvi-targetmiR482-3p_182
pvi-targetmiR482-3p_183
pvi-targetmiR482-3p_184
pvi-targetmiR482-3p_185
pvi-targetmiR482-3p_186
pvi-targetmiR482-3p_187
pvi-targetmiR482-3p_188
sbi-targetmiR482-3p_1
sbi-targetmiR482-3p_2
sbi-targetmiR482-3p_3
sbi-targetmiR482-3p_4
sbi-targetmiR482-3p_5
sbi-targetmiR482-3p_6
sbi-targetmiR482-3p_7
sbi-targetmiR482-3p_8
sbi-targetmiR482-3p_9

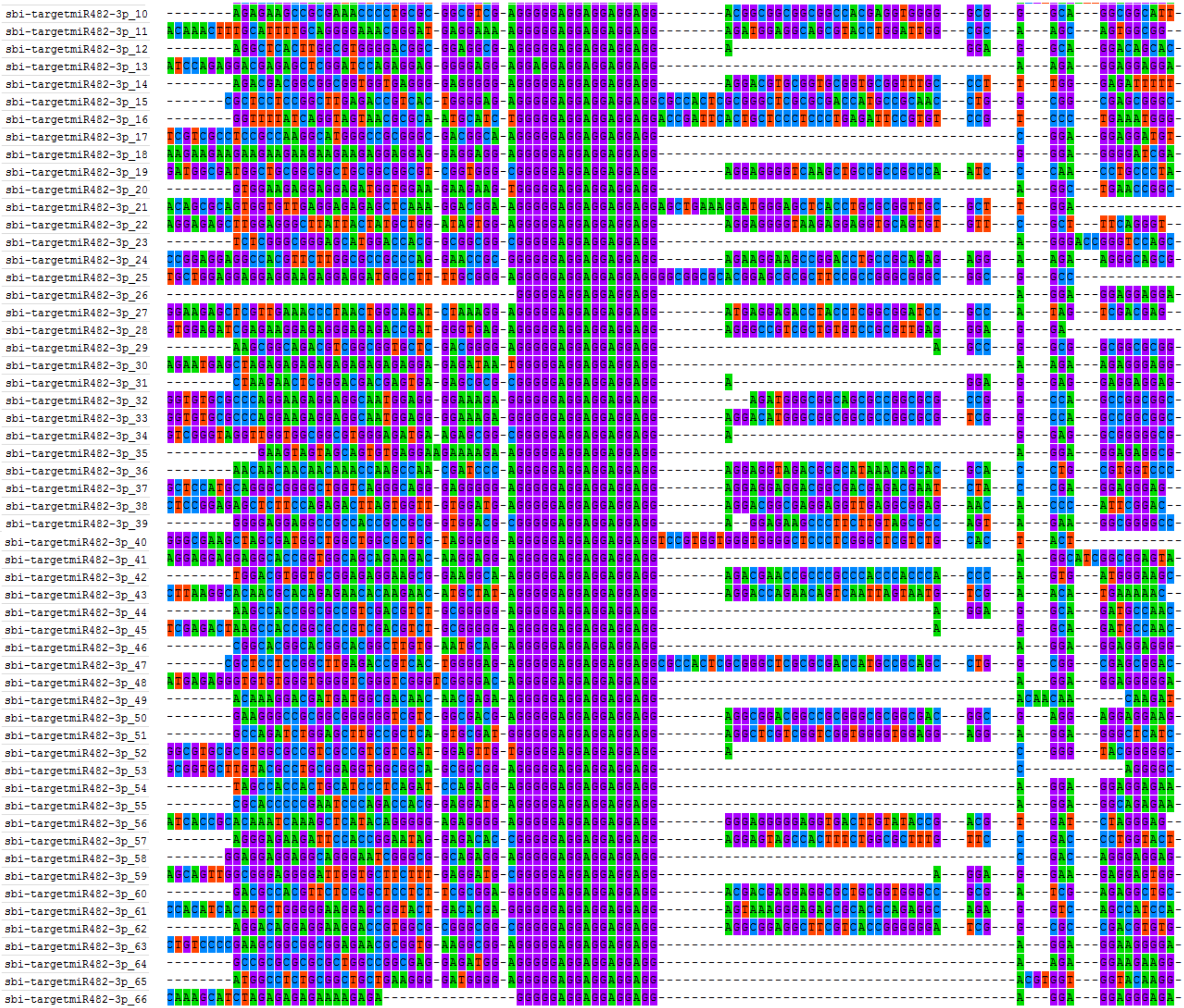
sbi-targetmiR482-3p_10
sbi-targetmiR482-3p_11
sbi-targetmiR482-3p_12
sbi-targetmiR482-3p_13
sbi-targetmiR482-3p_14
sbi-targetmiR482-3p_15
sbi-targetmiR482-3p_16
sbi-targetmiR482-3p_17
sbi-targetmiR482-3p_18
sbi-targetmiR482-3p_19
sbi-targetmiR482-3p_20
sbi-targetmiR482-3p_21
sbi-targetmiR482-3p_22
sbi-targetmiR482-3p_23
sbi-targetmiR482-3p_24
sbi-targetmiR482-3p_25
sbi-targetmiR482-3p_26
sbi-targetmiR482-3p_27
sbi-targetmiR482-3p_28
sbi-targetmiR482-3p_29
sbi-targetmiR482-3p_30
sbi-targetmiR482-3p_31
sbi-targetmiR482-3p_32
sbi-targetmiR482-3p_33
sbi-targetmiR482-3p_34
sbi-targetmiR482-3p_35
sbi-targetmiR482-3p_36
sbi-targetmiR482-3p_37
sbi-targetmiR482-3p_38
sbi-targetmiR482-3p_39
sbi-targetmiR482-3p_40
sbi-targetmiR482-3p_41
sbi-targetmiR482-3p_42
sbi-targetmiR482-3p_43
sbi-targetmiR482-3p_44
sbi-targetmiR482-3p_45
sbi-targetmiR482-3p_46
sbi-targetmiR482-3p_47
sbi-targetmiR482-3p_48
sbi-targetmiR482-3p_49
sbi-targetmiR482-3p_50
sbi-targetmiR482-3p_51
sbi-targetmiR482-3p_52
sbi-targetmiR482-3p_53
sbi-targetmiR482-3p_54
sbi-targetmiR482-3p_55
sbi-targetmiR482-3p_56
sbi-targetmiR482-3p_57
sbi-targetmiR482-3p_58
sbi-targetmiR482-3p_59
sbi-targetmiR482-3p_60
sbi-targetmiR482-3p_61
sbi-targetmiR482-3p_62
sbi-targetmiR482-3p_63
sbi-targetmiR482-3p_64
sbi-targetmiR482-3p_65
sbi-targetmiR482-3p_66

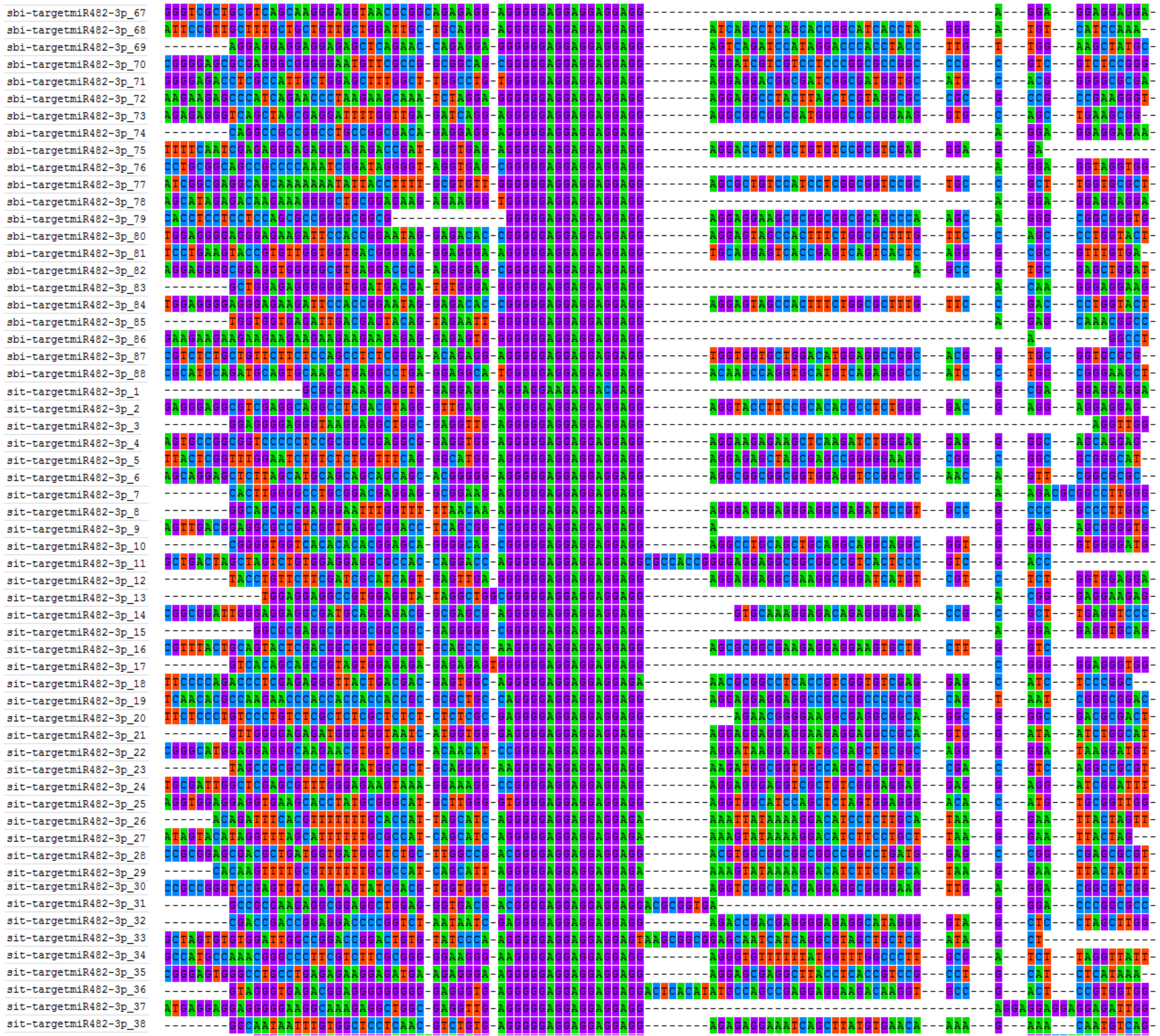
sbi-targetmiR482-3p_67
sbi-targetmiR482-3p_68
sbi-targetmiR482-3p_69
sbi-targetmiR482-3p_70
sbi-targetmiR482-3p_71
sbi-targetmiR482-3p_72
sbi-targetmiR482-3p_73
sbi-targetmiR482-3p_74
sbi-targetmiR482-3p_75
sbi-targetmiR482-3p_76
sbi-targetmiR482-3p_77
sbi-targetmiR482-3p_78
sbi-targetmiR482-3p_79
sbi-targetmiR482-3p_80
sbi-targetmiR482-3p_81
sbi-targetmiR482-3p_82
sbi-targetmiR482-3p_83
sbi-targetmiR482-3p_84
sbi-targetmiR482-3p_85
sbi-targetmiR482-3p_86
sbi-targetmiR482-3p_87
sbi-targetmiR482-3p_88
sit-targetmiR482-3p_1
sit-targetmiR482-3p_2
sit-targetmiR482-3p_3
sit-targetmiR482-3p_4
sit-targetmiR482-3p_5
sit-targetmiR482-3p_6
sit-targetmiR482-3p_7
sit-targetmiR482-3p_8
sit-targetmiR482-3p_9
sit-targetmiR482-3p_10
sit-targetmiR482-3p_11
sit-targetmiR482-3p_12
sit-targetmiR482-3p_13
sit-targetmiR482-3p_14
sit-targetmiR482-3p_15
sit-targetmiR482-3p_16
sit-targetmiR482-3p_17
sit-targetmiR482-3p_18
sit-targetmiR482-3p_19
sit-targetmiR482-3p_20
sit-targetmiR482-3p_21
sit-targetmiR482-3p_22
sit-targetmiR482-3p_23
sit-targetmiR482-3p_24
sit-targetmiR482-3p_25
sit-targetmiR482-3p_26
sit-targetmiR482-3p_27
sit-targetmiR482-3p_28
sit-targetmiR482-3p_29
sit-targetmiR482-3p_30
sit-targetmiR482-3p_31
sit-targetmiR482-3p_32
sit-targetmiR482-3p_33
sit-targetmiR482-3p_34
sit-targetmiR482-3p_35
sit-targetmiR482-3p_36
sit-targetmiR482-3p_37
sit-targetmiR482-3p_38

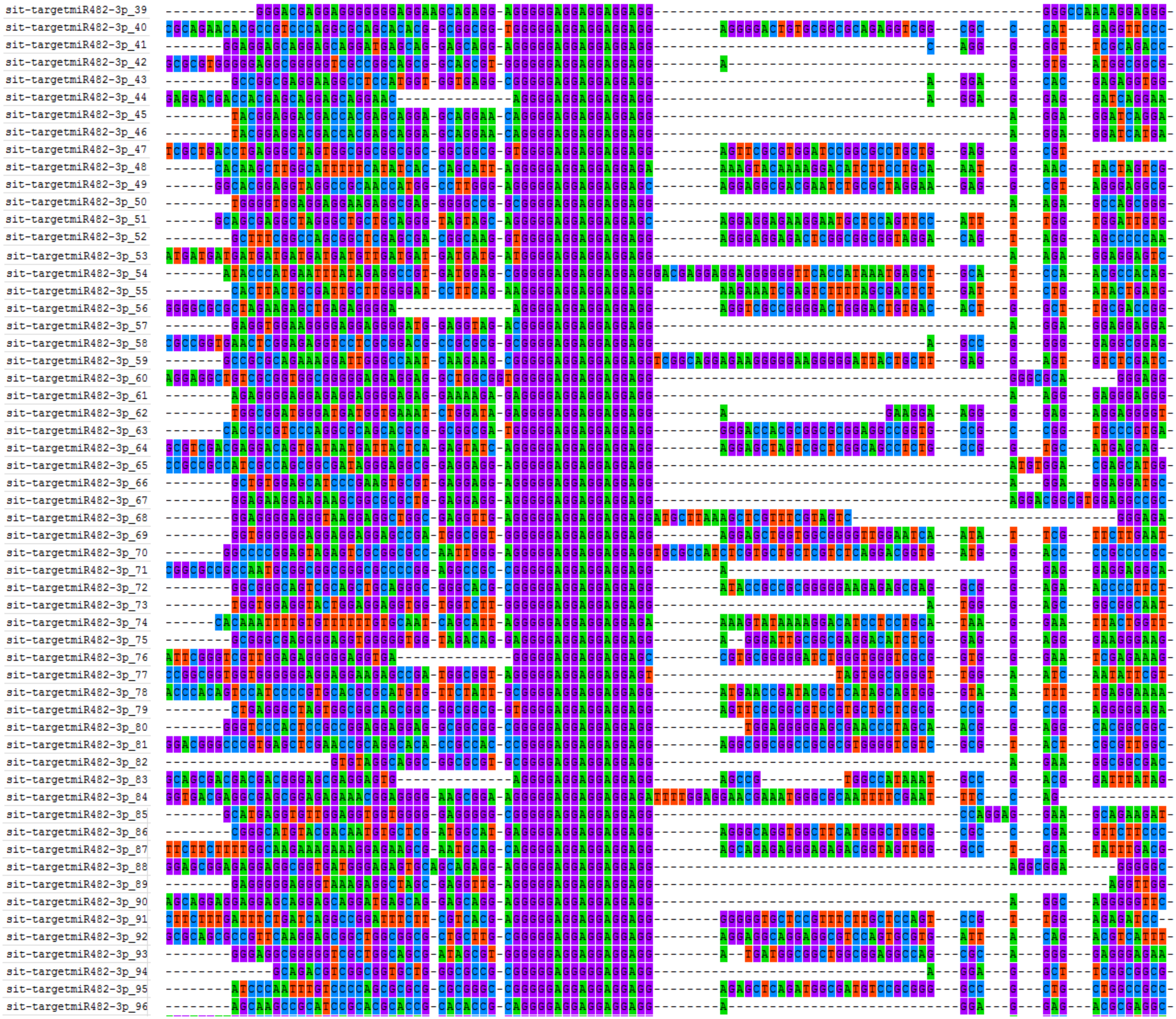
sit-targetmiR482-3p_39
sit-targetmiR482-3p_40
sit-targetmiR482-3p_41
sit-targetmiR482-3p_42
sit-targetmiR482-3p_43
sit-targetmiR482-3p_44
sit-targetmiR482-3p_45
sit-targetmiR482-3p_46
sit-targetmiR482-3p_47
sit-targetmiR482-3p_48
sit-targetmiR482-3p_49
sit-targetmiR482-3p_50
sit-targetmiR482-3p_51
sit-targetmiR482-3p_52
sit-targetmiR482-3p_53
sit-targetmiR482-3p_54
sit-targetmiR482-3p_55
sit-targetmiR482-3p_56
sit-targetmiR482-3p_57
sit-targetmiR482-3p_58
sit-targetmiR482-3p_59
sit-targetmiR482-3p_60
sit-targetmiR482-3p_61
sit-targetmiR482-3p_62
sit-targetmiR482-3p_63
sit-targetmiR482-3p_64
sit-targetmiR482-3p_65
sit-targetmiR482-3p_66
sit-targetmiR482-3p_67
sit-targetmiR482-3p_68
sit-targetmiR482-3p_69
sit-targetmiR482-3p_70
sit-targetmiR482-3p_71
sit-targetmiR482-3p_72
sit-targetmiR482-3p_73
sit-targetmiR482-3p_74
sit-targetmiR482-3p_75
sit-targetmiR482-3p_76
sit-targetmiR482-3p_77
sit-targetmiR482-3p_78
sit-targetmiR482-3p_79
sit-targetmiR482-3p_80
sit-targetmiR482-3p_81
sit-targetmiR482-3p_82
sit-targetmiR482-3p_83
sit-targetmiR482-3p_84
sit-targetmiR482-3p_85
sit-targetmiR482-3p_86
sit-targetmiR482-3p_87
sit-targetmiR482-3p_88
sit-targetmiR482-3p_89
sit-targetmiR482-3p_90
sit-targetmiR482-3p_91
sit-targetmiR482-3p_92
sit-targetmiR482-3p_93
sit-targetmiR482-3p_94
sit-targetmiR482-3p_95
sit-targetmiR482-3p_96

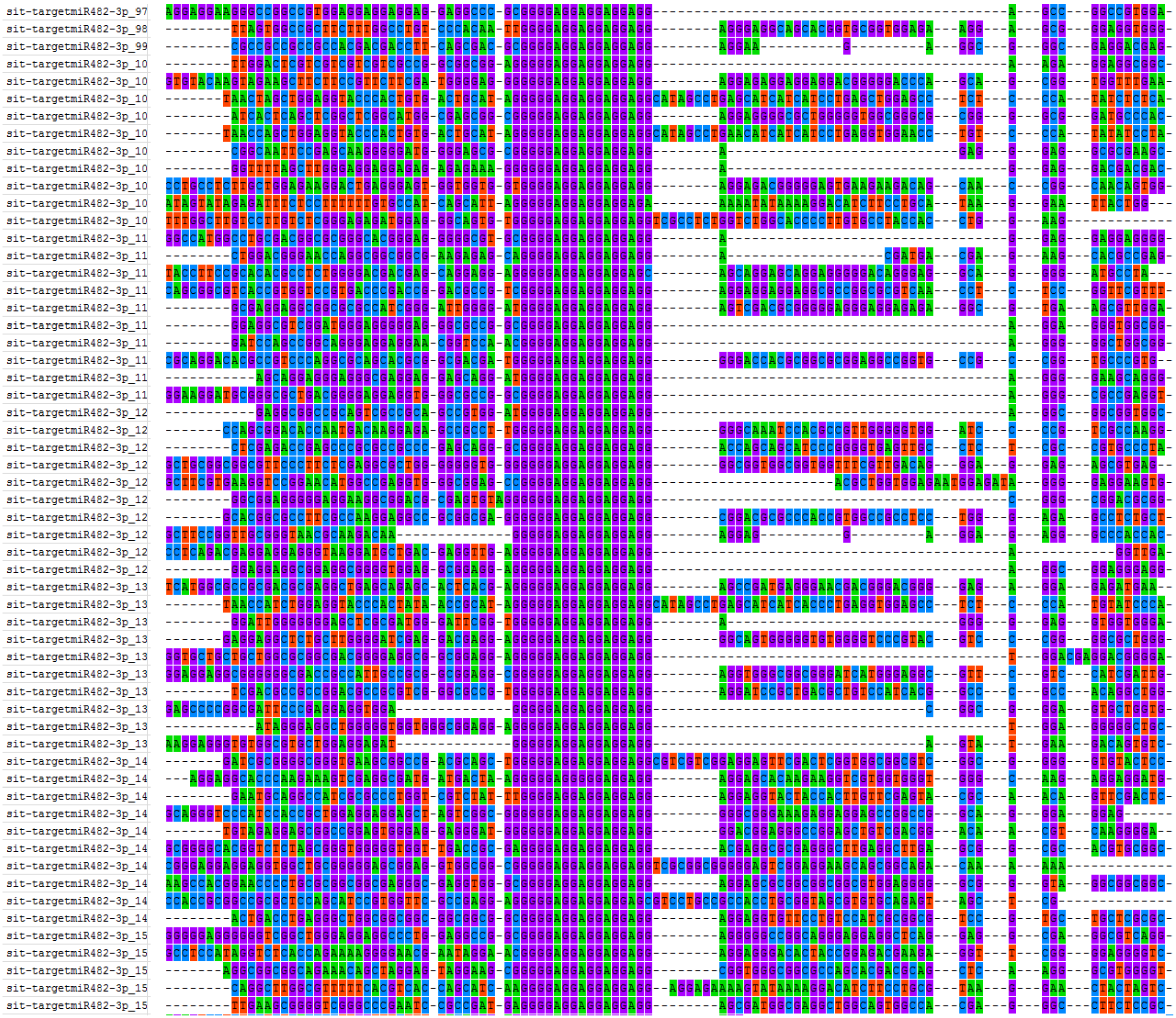
sit-targetmiR482-3p_97
sit-targetmiR482-3p_98
sit-targetmiR482-3p_99
sit-targetmiR482-3p_10
sit-targetmiR482-3p_10
sit-targetmiR482-3p_10
sit-targetmiR482-3p_10
sit-targetmiR482-3p_10
sit-targetmiR482-3p_10
sit-targetmiR482-3p_10
sit-targetmiR482-3p_10
sit-targetmiR482-3p_10
sit-targetmiR482-3p_10
sit-targetmiR482-3p_11
sit-targetmiR482-3p_11
sit-targetmiR482-3p_11
sit-targetmiR482-3p_11
sit-targetmiR482-3p_11
sit-targetmiR482-3p_11
sit-targetmiR482-3p_11
sit-targetmiR482-3p_11
sit-targetmiR482-3p_11
sit-targetmiR482-3p_11
sit-targetmiR482-3p_12
sit-targetmiR482-3p_12
sit-targetmiR482-3p_12
sit-targetmiR482-3p_12
sit-targetmiR482-3p_12
sit-targetmiR482-3p_12
sit-targetmiR482-3p_12
sit-targetmiR482-3p_12
sit-targetmiR482-3p_12
sit-targetmiR482-3p_12
sit-targetmiR482-3p_13
sit-targetmiR482-3p_13
sit-targetmiR482-3p_13
sit-targetmiR482-3p_13
sit-targetmiR482-3p_13
sit-targetmiR482-3p_13
sit-targetmiR482-3p_13
sit-targetmiR482-3p_13
sit-targetmiR482-3p_13
sit-targetmiR482-3p_13
sit-targetmiR482-3p_14
sit-targetmiR482-3p_14
sit-targetmiR482-3p_14
sit-targetmiR482-3p_14
sit-targetmiR482-3p_14
sit-targetmiR482-3p_14
sit-targetmiR482-3p_14
sit-targetmiR482-3p_14
sit-targetmiR482-3p_14
sit-targetmiR482-3p_14
sit-targetmiR482-3p_15
sit-targetmiR482-3p_15
sit-targetmiR482-3p_15
sit-targetmiR482-3p_15
sit-targetmiR482-3p_15

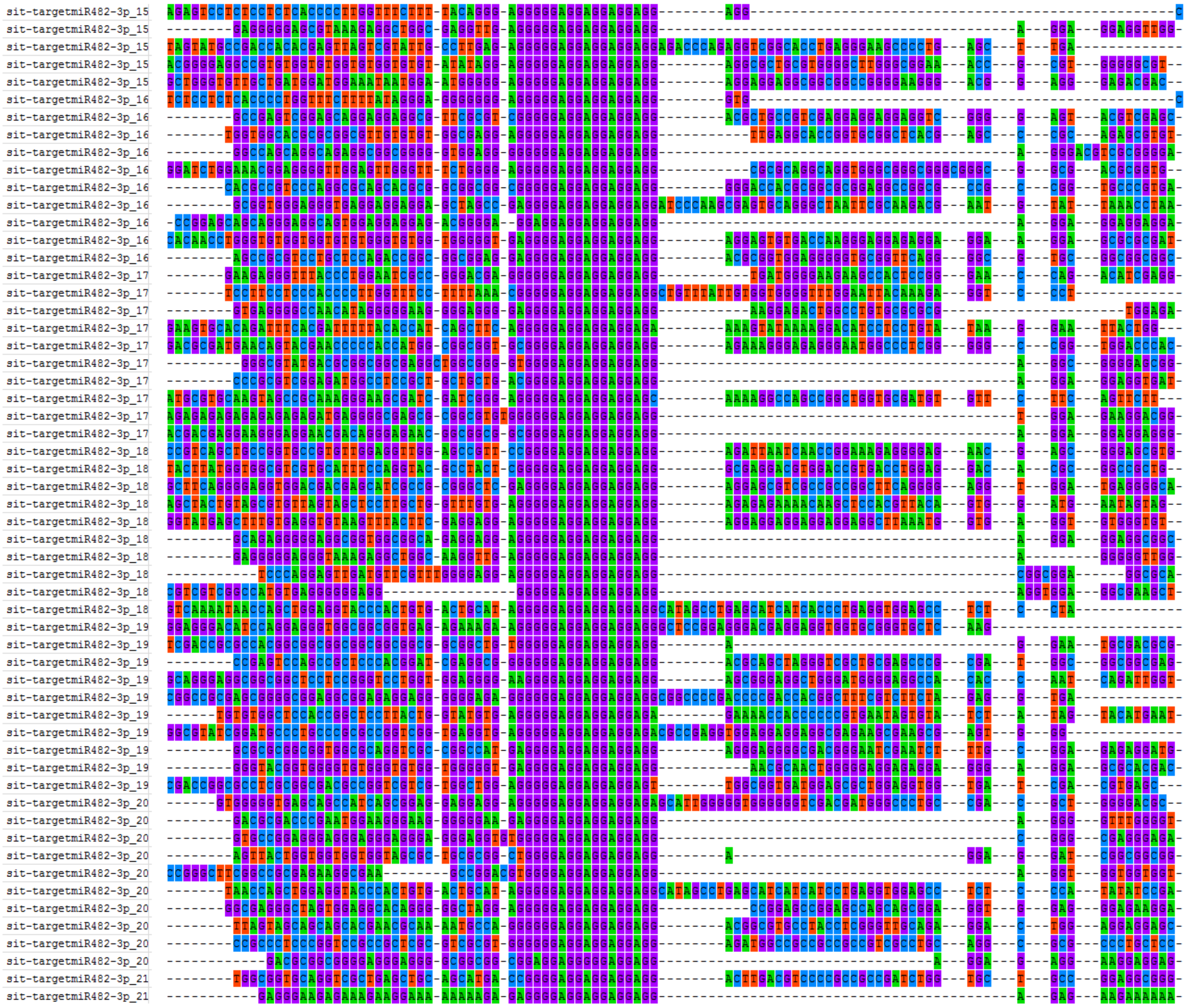

sit-targetmiR482-3p_15
sit-targetmiR482-3p_15
sit-targetmiR482-3p_15
sit-targetmiR482-3p_15
sit-targetmiR482-3p_15
sit-targetmiR482-3p_16
sit-targetmiR482-3p_16
sit-targetmiR482-3p_16
sit-targetmiR482-3p_16
sit-targetmiR482-3p_16
sit-targetmiR482-3p_16
sit-targetmiR482-3p_16
sit-targetmiR482-3p_16
sit-targetmiR482-3p_16
sit-targetmiR482-3p_16
sit-targetmiR482-3p_17
sit-targetmiR482-3p_17
sit-targetmiR482-3p_17
sit-targetmiR482-3p_17
sit-targetmiR482-3p_17
sit-targetmiR482-3p_17
sit-targetmiR482-3p_17
sit-targetmiR482-3p_17
sit-targetmiR482-3p_17
sit-targetmiR482-3p_17
sit-targetmiR482-3p_18
sit-targetmiR482-3p_18
sit-targetmiR482-3p_18
sit-targetmiR482-3p_18
sit-targetmiR482-3p_18
sit-targetmiR482-3p_18
sit-targetmiR482-3p_18
sit-targetmiR482-3p_18
sit-targetmiR482-3p_18
sit-targetmiR482-3p_18
sit-targetmiR482-3p_19
sit-targetmiR482-3p_19
sit-targetmiR482-3p_19
sit-targetmiR482-3p_19
sit-targetmiR482-3p_19
sit-targetmiR482-3p_19
sit-targetmiR482-3p_19
sit-targetmiR482-3p_19
sit-targetmiR482-3p_19
sit-targetmiR482-3p_19
sit-targetmiR482-3p_20
sit-targetmiR482-3p_20
sit-targetmiR482-3p_20
sit-targetmiR482-3p_20
sit-targetmiR482-3p_20
sit-targetmiR482-3p_20
sit-targetmiR482-3p_20
sit-targetmiR482-3p_20
sit-targetmiR482-3p_20
sit-targetmiR482-3p_20
sit-targetmiR482-3p_21
sit-targetmiR482-3p_21

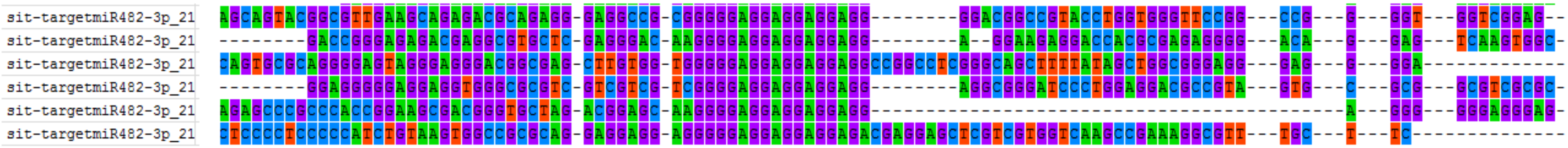
sit-targetmiR482-3p_21 AGCAGTACGGCGTTGAAGCAGAGACGCAGAGG-GAGGCCG-CGGGGGAGGAGGAGGAGG--------GGACGGCCGTACCTGGTGGGTTCCGG---CCG---G---GGT---GGTCGGAG--
sit-targetmiR482-3p_21 --------GACCGGGAGAGACGAGGCGTGCTC-GAGGGAC-AAGGGGAGGAGGAGGAGG--------A--GGAAGAGGACCACGCGAGAGGGG---ACA---G---GAG---TCAAGTGGC-
sit-targetmiR482-3p_21 CAGTGCGCAGGGGAGTAGGGAGGGACGGCGAG-CTTGTGG-TGGGGGAGGAGGAGGAGGCCGGCCTCGGGCAGCTTTTATAGCTGGCGGGAGG---GAG---G---GGA------------
sit-targetmiR482-3p_21 --------GGAGGGGGAGGAGGTGGGCGCGTC-GTCGTCG-TCGGGGAGGAGGAGGAGG--------AGGCGGGATCCCTGGAGGACGCCGTA---GTG---C---GCG---GCGTCGCGC-
sit-targetmiR482-3p_21 AGAGCCCGCCCACCGGAAGCGACGGGTGCTAG-ACGGAGC-AAGGGGAGGAGGAGGAGG--A---GGG---GGGAGGGAG-
sit-targetmiR482-3p_21 CTCCCCTCCCCCATCTGTAAGTGGCCGCGCAG-GAGGAGG-AGGGGGAGGAGGAGGAGACGAGGAGCTCGTCGTGGTCAAGCCGAAAGGCGTT---TGC---T---TC------------
