# Supplementary material for: Genome-wide identification and functional analysis of lincRNAs acting as miRNA targets or decoys in maize
Source: BMC Genomics. 2015 Oct 15;16:793. doi: 10.1186/s12864-015-2024-0 (PMC4608266; doi:10.1186/s12864-015-2024-0)

```
Boerner_Z27kG1_01046: 5' GGUGGACGAGGUGGUGCAUGGG 3'
                         oo|||| ||||oo| |||oo||
   zma-miR528a/b-3p: 3' UUACCUUCUCCGUC-CGUGUCC 5'

Boerner_Z27kG1_23730/Boerner_Z27kG1_08632: 5' AGUGGAGGCGGGGGGAGCGGG 3'
                                              |o||||o| || o|| o|o||
                        zma-miR528a/b-3p: 3' UUACCUUCUCCGUCCGUGUCC 5'
```

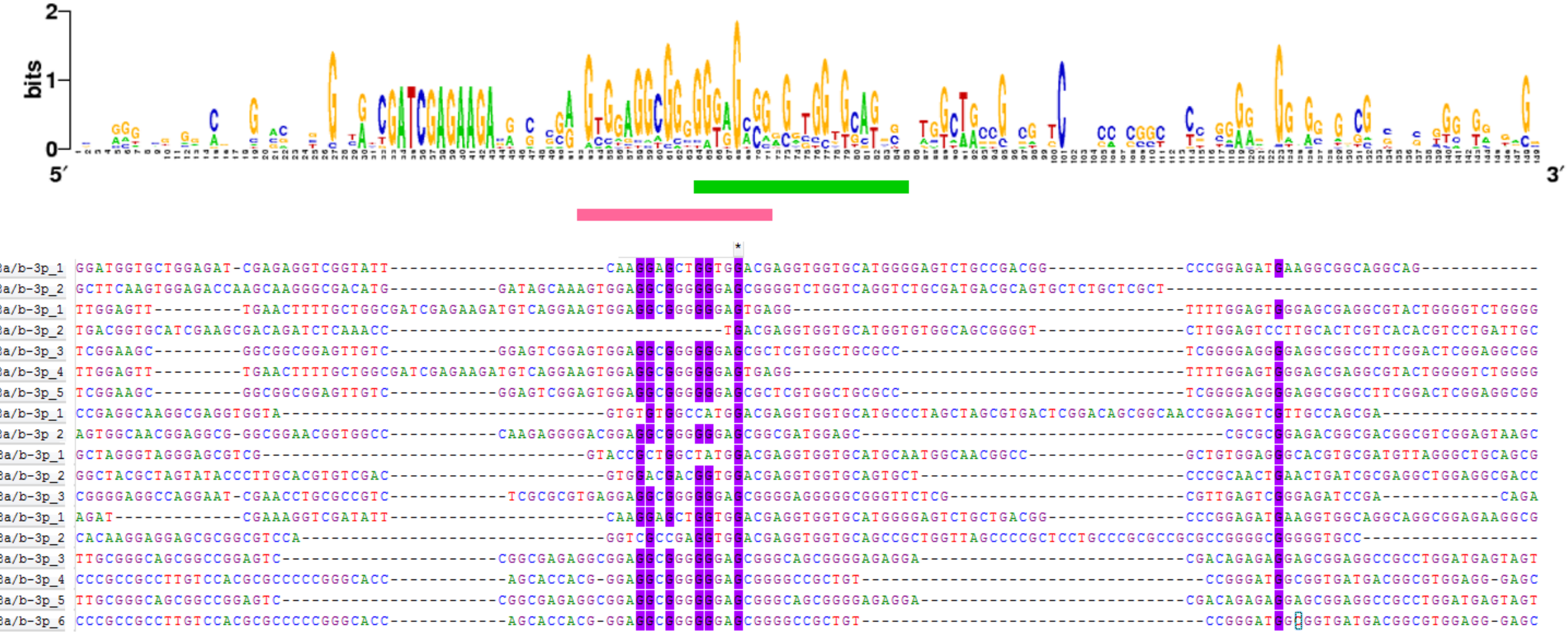

Supplement: Additional file 5: — The sequence logos of the 12 conserved lincRNAs as miRNA targets. (ZIP 3605 kb) [file 12864_2015_2024_MOESM5_ESM.zip › Additional file 5/target-528a-3p_528b-3p.pdf]
