# Supplementary material for: Genome-wide identification and functional analysis of lincRNAs acting as miRNA targets or decoys in maize
Source: BMC Genomics. 2015 Oct 15;16:793. doi: 10.1186/s12864-015-2024-0 (PMC4608266; doi:10.1186/s12864-015-2024-0)
Supplement: Additional file 5: — The sequence logos of the 12 conserved lincRNAs as miRNA targets. (ZIP 3605 kb) [file 12864_2015_2024_MOESM5_ESM.zip › Additional file 5/target-529-3p.pdf]

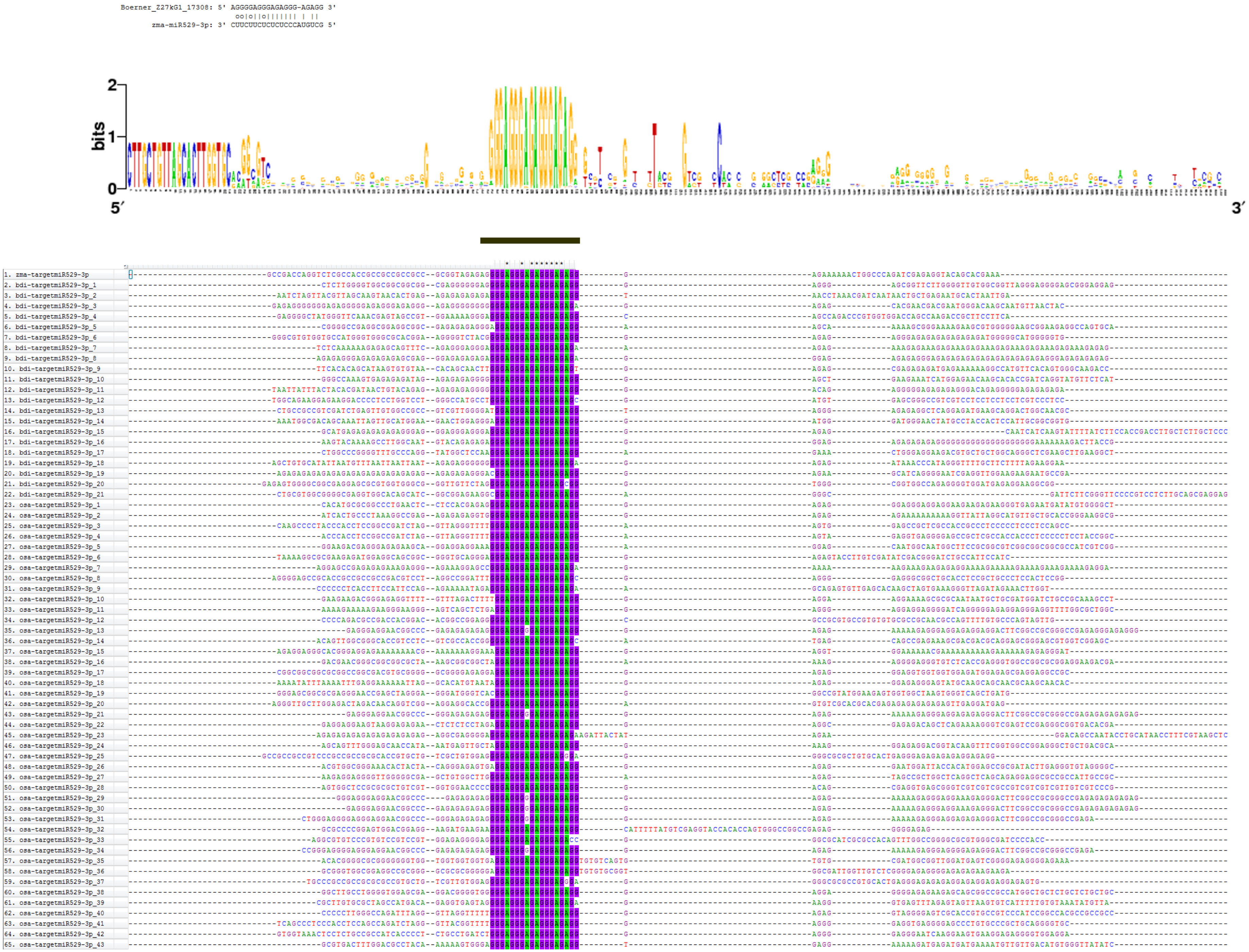

Boerner_Z27kG1_17308: 5' AGGGGAGGGAGAGGG-AGAGG 3'
oo|o||o||||||| | ||
zma-miR529-3p: 3' CUUCUUCUCUCUCCCAUGUCG 5'
bits
2
1
0
5′
3′
1. zma-targetmiR529-3p
2. bdi-targetmiR529-3p_1
3. bdi-targetmiR529-3p_2
4. bdi-targetmiR529-3p_3
5. bdi-targetmiR529-3p_4
6. bdi-targetmiR529-3p_5
7. bdi-targetmiR529-3p_6
8. bdi-targetmiR529-3p_7
9. bdi-targetmiR529-3p_8
10. bdi-targetmiR529-3p_9
11. bdi-targetmiR529-3p_10
12. bdi-targetmiR529-3p_11
13. bdi-targetmiR529-3p_12
14. bdi-targetmiR529-3p_13
15. bdi-targetmiR529-3p_14
16. bdi-targetmiR529-3p_15
17. bdi-targetmiR529-3p_16
18. bdi-targetmiR529-3p_17
19. bdi-targetmiR529-3p_18
20. bdi-targetmiR529-3p_19
21. bdi-targetmiR529-3p_20
22. bdi-targetmiR529-3p_21
23. osa-targetmiR529-3p_1
24. osa-targetmiR529-3p_2
25. osa-targetmiR529-3p_3
26. osa-targetmiR529-3p_4
27. osa-targetmiR529-3p_5
28. osa-targetmiR529-3p_6
29. osa-targetmiR529-3p_7
30. osa-targetmiR529-3p_8
31. osa-targetmiR529-3p_9
32. osa-targetmiR529-3p_10
33. osa-targetmiR529-3p_11
34. osa-targetmiR529-3p_12
35. osa-targetmiR529-3p_13
36. osa-targetmiR529-3p_14
37. osa-targetmiR529-3p_15
38. osa-targetmiR529-3p_16
39. osa-targetmiR529-3p_17
40. osa-targetmiR529-3p_18
41. osa-targetmiR529-3p_19
42. osa-targetmiR529-3p_20
43. osa-targetmiR529-3p_21
44. osa-targetmiR529-3p_22
45. osa-targetmiR529-3p_23
46. osa-targetmiR529-3p_24
47. osa-targetmiR529-3p_25
48. osa-targetmiR529-3p_26
49. osa-targetmiR529-3p_27
50. osa-targetmiR529-3p_28
51. osa-targetmiR529-3p_29
52. osa-targetmiR529-3p_30
53. osa-targetmiR529-3p_31
54. osa-targetmiR529-3p_32
55. osa-targetmiR529-3p_33
56. osa-targetmiR529-3p_34
57. osa-targetmiR529-3p_35
58. osa-targetmiR529-3p_36
59. osa-targetmiR529-3p_37
60. osa-targetmiR529-3p_38
61. osa-targetmiR529-3p_39
62. osa-targetmiR529-3p_40
63. osa-targetmiR529-3p_41
64. osa-targetmiR529-3p_42
65. osa-targetmiR529-3p_43

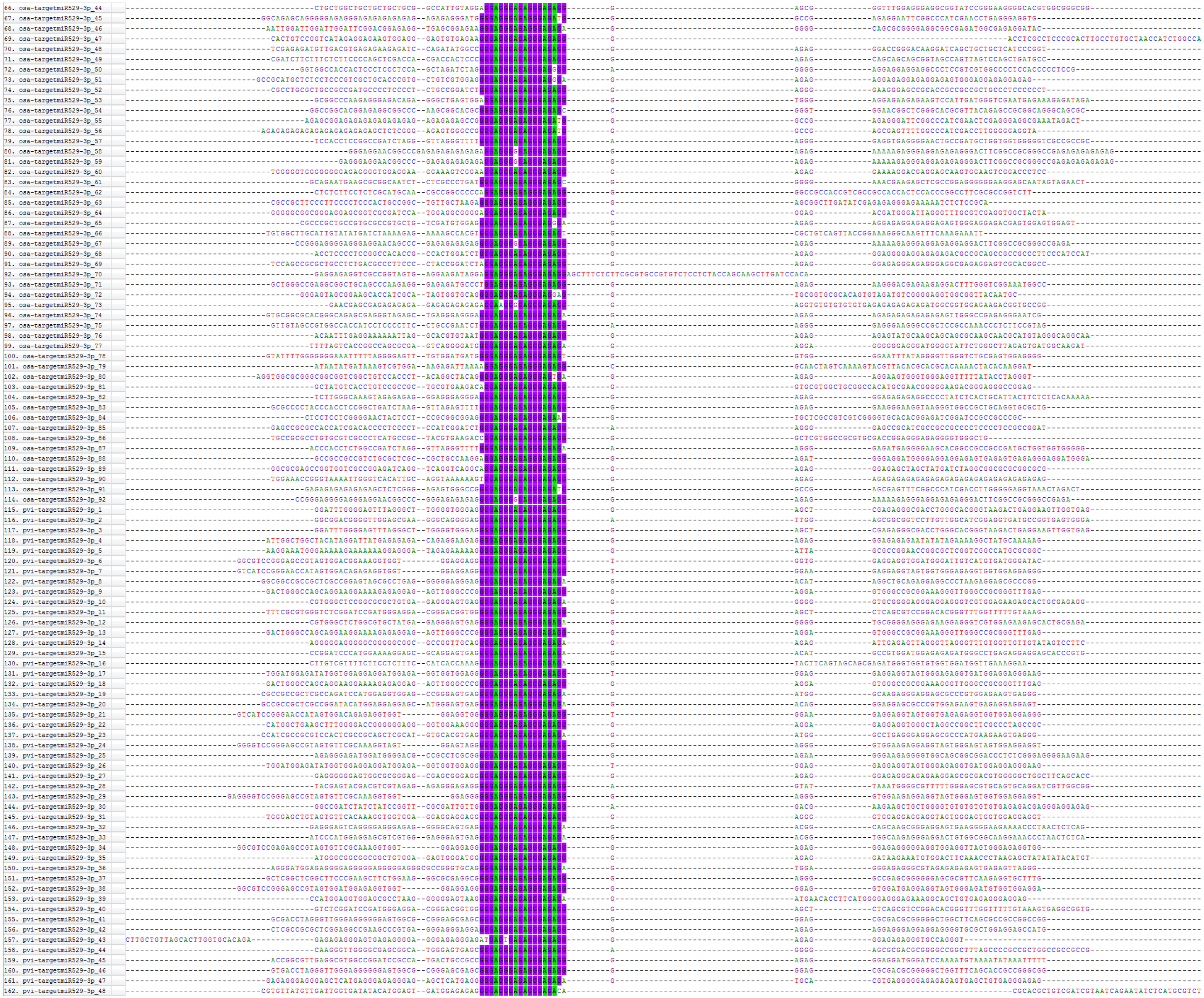
66. osa-targetmiR529-3p_44
67. osa-targetmiR529-3p_45
68. osa-targetmiR529-3p_46
69. osa-targetmiR529-3p_47
70. osa-targetmiR529-3p_48
71. osa-targetmiR529-3p_49
72. osa-targetmiR529-3p_50
73. osa-targetmiR529-3p_51
74. osa-targetmiR529-3p_52
75. osa-targetmiR529-3p_53
76. osa-targetmiR529-3p_54
77. osa-targetmiR529-3p_55
78. osa-targetmiR529-3p_56
79. osa-targetmiR529-3p_57
80. osa-targetmiR529-3p_58
81. osa-targetmiR529-3p_59
82. osa-targetmiR529-3p_60
83. osa-targetmiR529-3p_61
84. osa-targetmiR529-3p_62
85. osa-targetmiR529-3p_63
86. osa-targetmiR529-3p_64
87. osa-targetmiR529-3p_65
88. osa-targetmiR529-3p_66
89. osa-targetmiR529-3p_67
90. osa-targetmiR529-3p_68
91. osa-targetmiR529-3p_69
92. osa-targetmiR529-3p_70
93. osa-targetmiR529-3p_71
94. osa-targetmiR529-3p_72
95. osa-targetmiR529-3p_73
96. osa-targetmiR529-3p_74
97. osa-targetmiR529-3p_75
98. osa-targetmiR529-3p_76
99. osa-targetmiR529-3p_77
100. osa-targetmiR529-3p_78
101. osa-targetmiR529-3p_79
102. osa-targetmiR529-3p_80
103. osa-targetmiR529-3p_81
104. osa-targetmiR529-3p_82
105. osa-targetmiR529-3p_83
106. osa-targetmiR529-3p_84
107. osa-targetmiR529-3p_85
108. osa-targetmiR529-3p_86
109. osa-targetmiR529-3p_87
110. osa-targetmiR529-3p_88
111. osa-targetmiR529-3p_89
112. osa-targetmiR529-3p_90
113. osa-targetmiR529-3p_91
114. osa-targetmiR529-3p_92
115. pvi-targetmiR529-3p_1
116. pvi-targetmiR529-3p_2
117. pvi-targetmiR529-3p_3
118. pvi-targetmiR529-3p_4
119. pvi-targetmiR529-3p_5
120. pvi-targetmiR529-3p_6
121. pvi-targetmiR529-3p_7
122. pvi-targetmiR529-3p_8
123. pvi-targetmiR529-3p_9
124. pvi-targetmiR529-3p_10
125. pvi-targetmiR529-3p_11
126. pvi-targetmiR529-3p_12
127. pvi-targetmiR529-3p_13
128. pvi-targetmiR529-3p_14
129. pvi-targetmiR529-3p_15
130. pvi-targetmiR529-3p_16
131. pvi-targetmiR529-3p_17
132. pvi-targetmiR529-3p_18
133. pvi-targetmiR529-3p_19
134. pvi-targetmiR529-3p_20
135. pvi-targetmiR529-3p_21
136. pvi-targetmiR529-3p_22
137. pvi-targetmiR529-3p_23
138. pvi-targetmiR529-3p_24
139. pvi-targetmiR529-3p_25
140. pvi-targetmiR529-3p_26
141. pvi-targetmiR529-3p_27
142. pvi-targetmiR529-3p_28
143. pvi-targetmiR529-3p_29
144. pvi-targetmiR529-3p_30
145. pvi-targetmiR529-3p_31
146. pvi-targetmiR529-3p_32
147. pvi-targetmiR529-3p_33
148. pvi-targetmiR529-3p_34
149. pvi-targetmiR529-3p_35
150. pvi-targetmiR529-3p_36
151. pvi-targetmiR529-3p_37
152. pvi-targetmiR529-3p_38
153. pvi-targetmiR529-3p_39
154. pvi-targetmiR529-3p_40
155. pvi-targetmiR529-3p_41
156. pvi-targetmiR529-3p_42
157. pvi-targetmiR529-3p_43
158. pvi-targetmiR529-3p_44
159. pvi-targetmiR529-3p_45
160. pvi-targetmiR529-3p_46
161. pvi-targetmiR529-3p_47
162. pvi-targetmiR529-3p_48

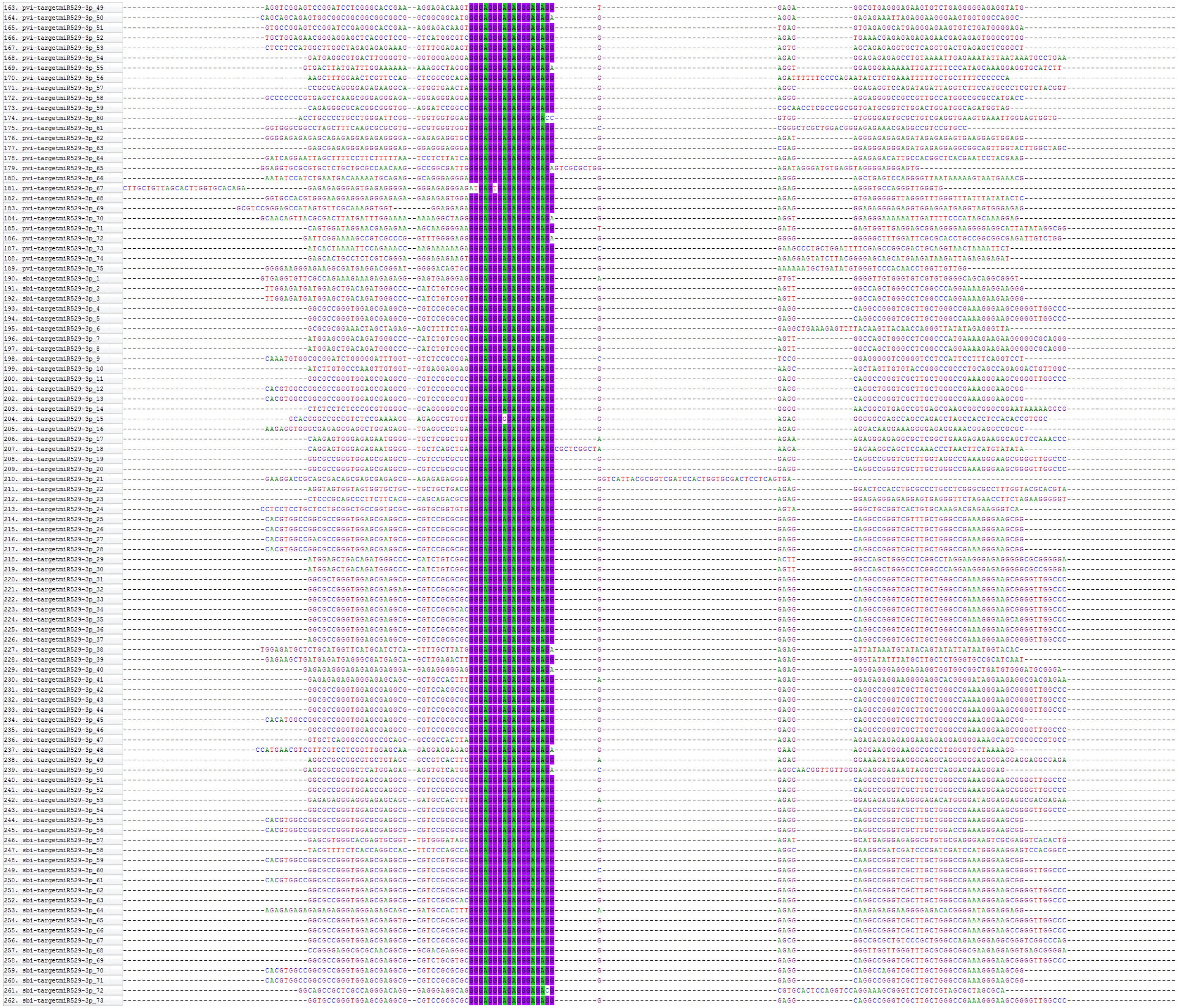
163. pvi-targetmiR529-3p_49
164. pvi-targetmiR529-3p_50
165. pvi-targetmiR529-3p_51
166. pvi-targetmiR529-3p_52
167. pvi-targetmiR529-3p_53
168. pvi-targetmiR529-3p_54
169. pvi-targetmiR529-3p_55
170. pvi-targetmiR529-3p_56
171. pvi-targetmiR529-3p_57
172. pvi-targetmiR529-3p_58
173. pvi-targetmiR529-3p_59
174. pvi-targetmiR529-3p_60
175. pvi-targetmiR529-3p_61
176. pvi-targetmiR529-3p_62
177. pvi-targetmiR529-3p_63
178. pvi-targetmiR529-3p_64
179. pvi-targetmiR529-3p_65
180. pvi-targetmiR529-3p_66
181. pvi-targetmiR529-3p_67
182. pvi-targetmiR529-3p_68
183. pvi-targetmiR529-3p_69
184. pvi-targetmiR529-3p_70
185. pvi-targetmiR529-3p_71
186. pvi-targetmiR529-3p_72
187. pvi-targetmiR529-3p_73
188. pvi-targetmiR529-3p_74
189. pvi-targetmiR529-3p_75
190. sbi-targetmiR529-3p_1
191. sbi-targetmiR529-3p_2
192. sbi-targetmiR529-3p_3
193. sbi-targetmiR529-3p_4
194. sbi-targetmiR529-3p_5
195. sbi-targetmiR529-3p_6
196. sbi-targetmiR529-3p_7
197. sbi-targetmiR529-3p_8
198. sbi-targetmiR529-3p_9
199. sbi-targetmiR529-3p_10
200. sbi-targetmiR529-3p_11
201. sbi-targetmiR529-3p_12
202. sbi-targetmiR529-3p_13
203. sbi-targetmiR529-3p_14
204. sbi-targetmiR529-3p_15
205. sbi-targetmiR529-3p_16
206. sbi-targetmiR529-3p_17
207. sbi-targetmiR529-3p_18
208. sbi-targetmiR529-3p_19
209. sbi-targetmiR529-3p_20
210. sbi-targetmiR529-3p_21
211. sbi-targetmiR529-3p_22
212. sbi-targetmiR529-3p_23
213. sbi-targetmiR529-3p_24
214. sbi-targetmiR529-3p_25
215. sbi-targetmiR529-3p_26
216. sbi-targetmiR529-3p_27
217. sbi-targetmiR529-3p_28
218. sbi-targetmiR529-3p_29
219. sbi-targetmiR529-3p_30
220. sbi-targetmiR529-3p_31
221. sbi-targetmiR529-3p_32
222. sbi-targetmiR529-3p_33
223. sbi-targetmiR529-3p_34
224. sbi-targetmiR529-3p_35
225. sbi-targetmiR529-3p_36
226. sbi-targetmiR529-3p_37
227. sbi-targetmiR529-3p_38
228. sbi-targetmiR529-3p_39
229. sbi-targetmiR529-3p_40
230. sbi-targetmiR529-3p_41
231. sbi-targetmiR529-3p_42
232. sbi-targetmiR529-3p_43
233. sbi-targetmiR529-3p_44
234. sbi-targetmiR529-3p_45
235. sbi-targetmiR529-3p_46
236. sbi-targetmiR529-3p_47
237. sbi-targetmiR529-3p_48
238. sbi-targetmiR529-3p_49
239. sbi-targetmiR529-3p_50
240. sbi-targetmiR529-3p_51
241. sbi-targetmiR529-3p_52
242. sbi-targetmiR529-3p_53
243. sbi-targetmiR529-3p_54
244. sbi-targetmiR529-3p_55
245. sbi-targetmiR529-3p_56
246. sbi-targetmiR529-3p_57
247. sbi-targetmiR529-3p_58
248. sbi-targetmiR529-3p_59
249. sbi-targetmiR529-3p_60
250. sbi-targetmiR529-3p_61
251. sbi-targetmiR529-3p_62
252. sbi-targetmiR529-3p_63
253. sbi-targetmiR529-3p_64
254. sbi-targetmiR529-3p_65
255. sbi-targetmiR529-3p_66
256. sbi-targetmiR529-3p_67
257. sbi-targetmiR529-3p_68
258. sbi-targetmiR529-3p_69
259. sbi-targetmiR529-3p_70
260. sbi-targetmiR529-3p_71
261. sbi-targetmiR529-3p_72
262. sbi-targetmiR529-3p_73

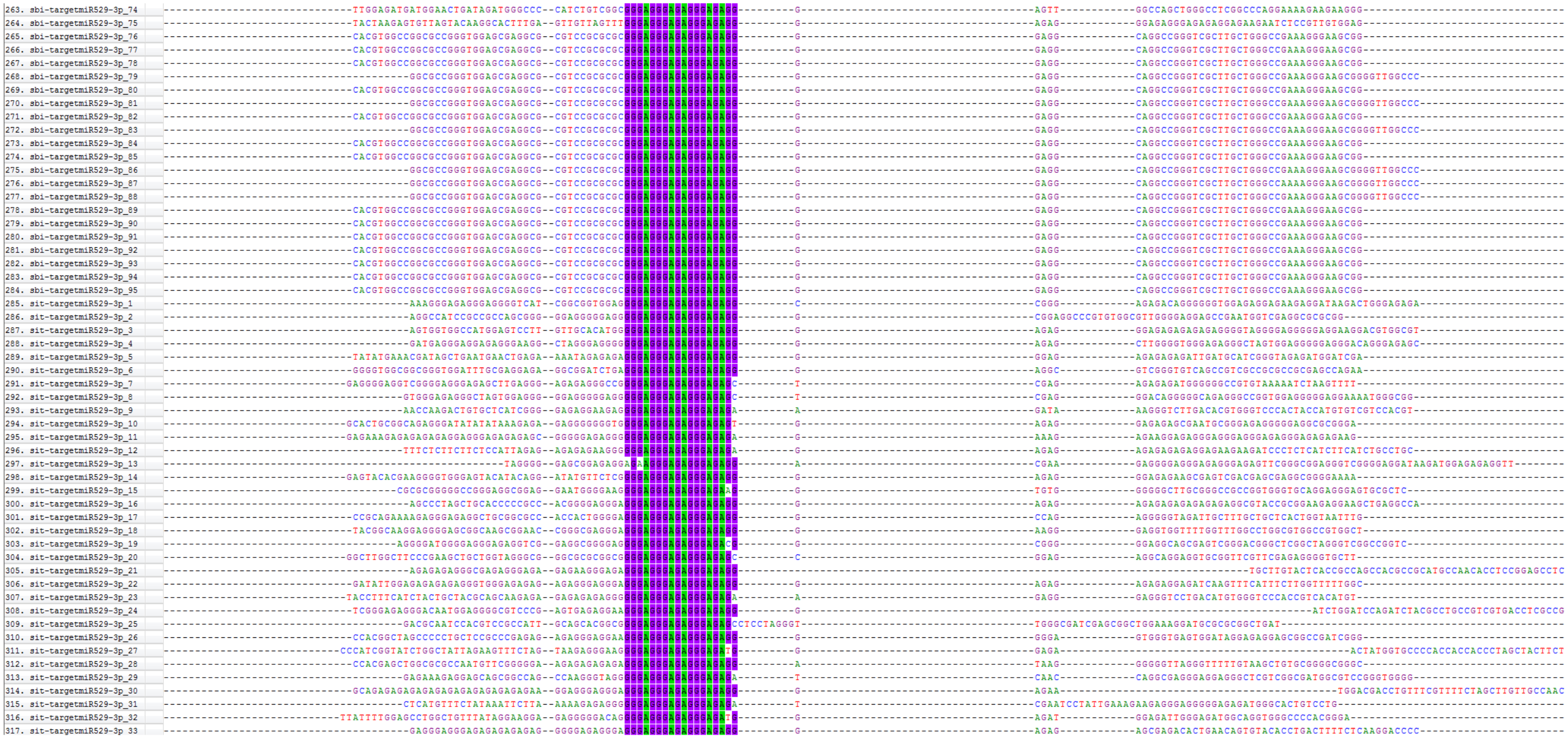
263. sbi-targetmiR529-3p_74
264. sbi-targetmiR529-3p_75
265. sbi-targetmiR529-3p_76
266. sbi-targetmiR529-3p_77
267. sbi-targetmiR529-3p_78
268. sbi-targetmiR529-3p_79
269. sbi-targetmiR529-3p_80
270. sbi-targetmiR529-3p_81
271. sbi-targetmiR529-3p_82
272. sbi-targetmiR529-3p_83
273. sbi-targetmiR529-3p_84
274. sbi-targetmiR529-3p_85
275. sbi-targetmiR529-3p_86
276. sbi-targetmiR529-3p_87
277. sbi-targetmiR529-3p_88
278. sbi-targetmiR529-3p_89
279. sbi-targetmiR529-3p_90
280. sbi-targetmiR529-3p_91
281. sbi-targetmiR529-3p_92
282. sbi-targetmiR529-3p_93
283. sbi-targetmiR529-3p_94
284. sbi-targetmiR529-3p_95
285. sit-targetmiR529-3p_1
286. sit-targetmiR529-3p_2
287. sit-targetmiR529-3p_3
288. sit-targetmiR529-3p_4
289. sit-targetmiR529-3p_5
290. sit-targetmiR529-3p_6
291. sit-targetmiR529-3p_7
292. sit-targetmiR529-3p_8
293. sit-targetmiR529-3p_9
294. sit-targetmiR529-3p_10
295. sit-targetmiR529-3p_11
296. sit-targetmiR529-3p_12
297. sit-targetmiR529-3p_13
298. sit-targetmiR529-3p_14
299. sit-targetmiR529-3p_15
300. sit-targetmiR529-3p_16
301. sit-targetmiR529-3p_17
302. sit-targetmiR529-3p_18
303. sit-targetmiR529-3p_19
304. sit-targetmiR529-3p_20
305. sit-targetmiR529-3p_21
306. sit-targetmiR529-3p_22
307. sit-targetmiR529-3p_23
308. sit-targetmiR529-3p_24
309. sit-targetmiR529-3p_25
310. sit-targetmiR529-3p_26
311. sit-targetmiR529-3p_27
312. sit-targetmiR529-3p_28
313. sit-targetmiR529-3p_29
314. sit-targetmiR529-3p_30
315. sit-targetmiR529-3p_31
316. sit-targetmiR529-3p_32
317. sit-targetmiR529-3p_33
