# Supplementary figures and images for: Genome-wide identification and functional analysis of lincRNAs acting as miRNA targets or decoys in maize
Source: BMC Genomics. 2015 Oct 15;16:793. doi: 10.1186/s12864-015-2024-0 (PMC4608266; doi:10.1186/s12864-015-2024-0)

oo | | | | | | | | | |

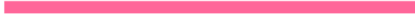[illegible]

Supplement: Additional file 5: — The sequence logos of the 12 conserved lincRNAs as miRNA targets. (ZIP 3605 kb) [file 12864_2015_2024_MOESM5_ESM.zip › Additional file 5/target-160b-3p_160g-3p.pdf]

```
Boerner_Z27kG1_01735: 5' AGAGCG-GGCACGUCAUGCACGC 3'
                        o || |||| |||||
zma-miR160c-3p: 3' AUACGAACCGUG--GUACGUGCG 5'
```

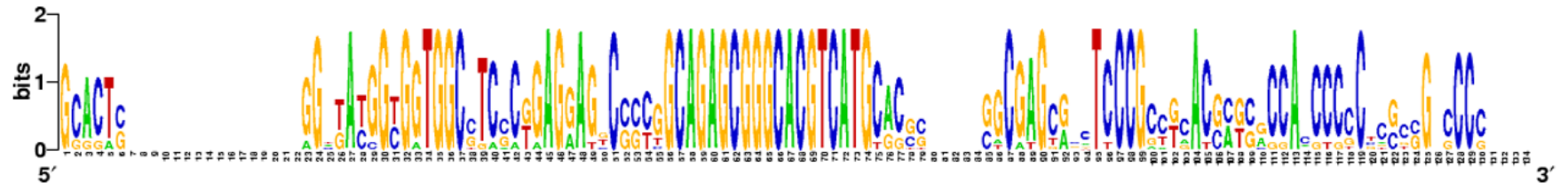[illegible]

Supplement: Additional file 7: — The sequence logos of the 10 conserved lincRNA as miRNA decoys. (ZIP 1503 kb) [file 12864_2015_2024_MOESM7_ESM.zip › Additional file 7/eTM-160c-3p.pdf]

```

      ||o|||||  |oo||oo|
zma-miR395k-5p: 3' UACACUUCACGAA--CUUCCUUUG 5'

```

zma-miR395k-5p: 3' UACACUUCACGAA---CUCCUUUG 5'

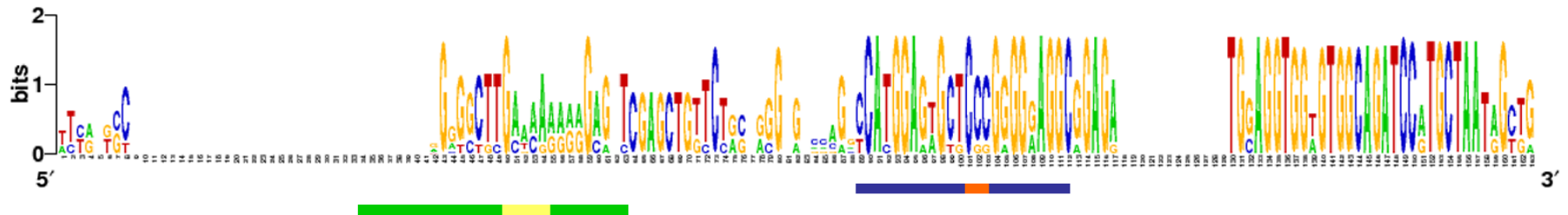[illegible]

Supplement: Additional file 7: — The sequence logos of the 10 conserved lincRNA as miRNA decoys. (ZIP 1503 kb) [file 12864_2015_2024_MOESM7_ESM.zip › Additional file 7/eTM-395k-5p.pdf]

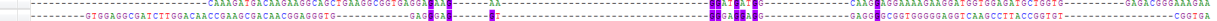

Supplement: Additional file 7: — The sequence logos of the 10 conserved lincRNA as miRNA decoys. (ZIP 1503 kb) [file 12864_2015_2024_MOESM7_ESM.zip › Additional file 7/eTM-482-3p.pdf]

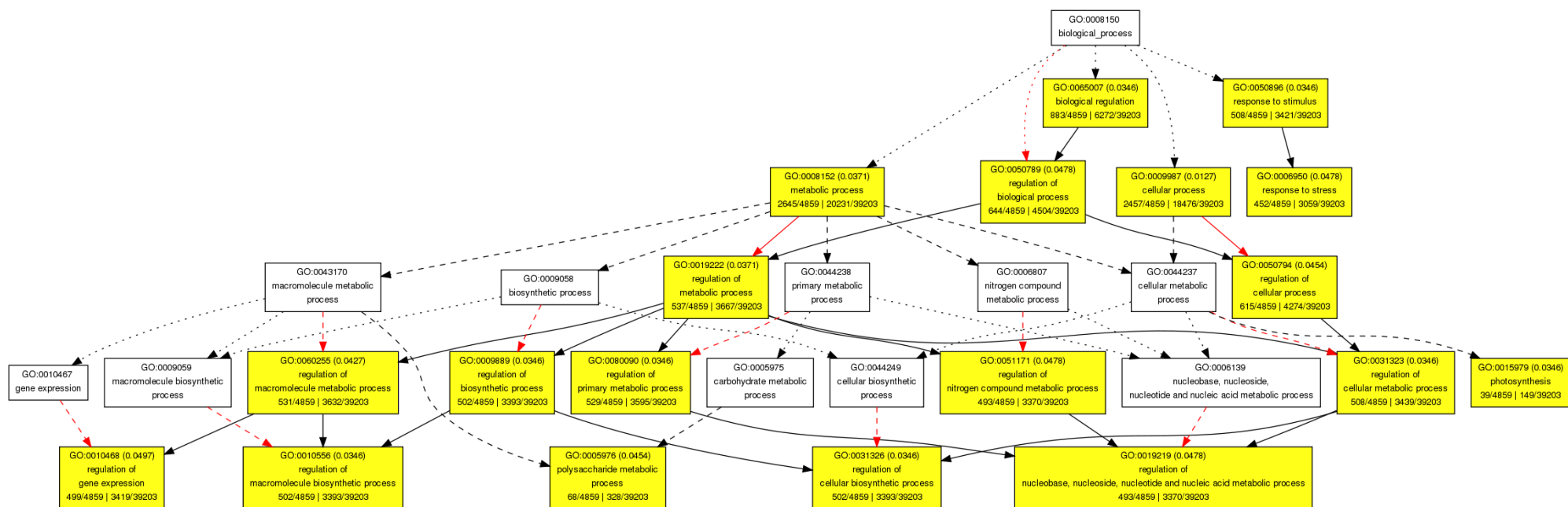

Supplement: Additional file 12: — GO enrichment analysis of maize lincRNAs functioning as miRNA targets associated with “biological processes”. (PDF 153 kb) [file 12864_2015_2024_MOESM12_ESM.pdf]

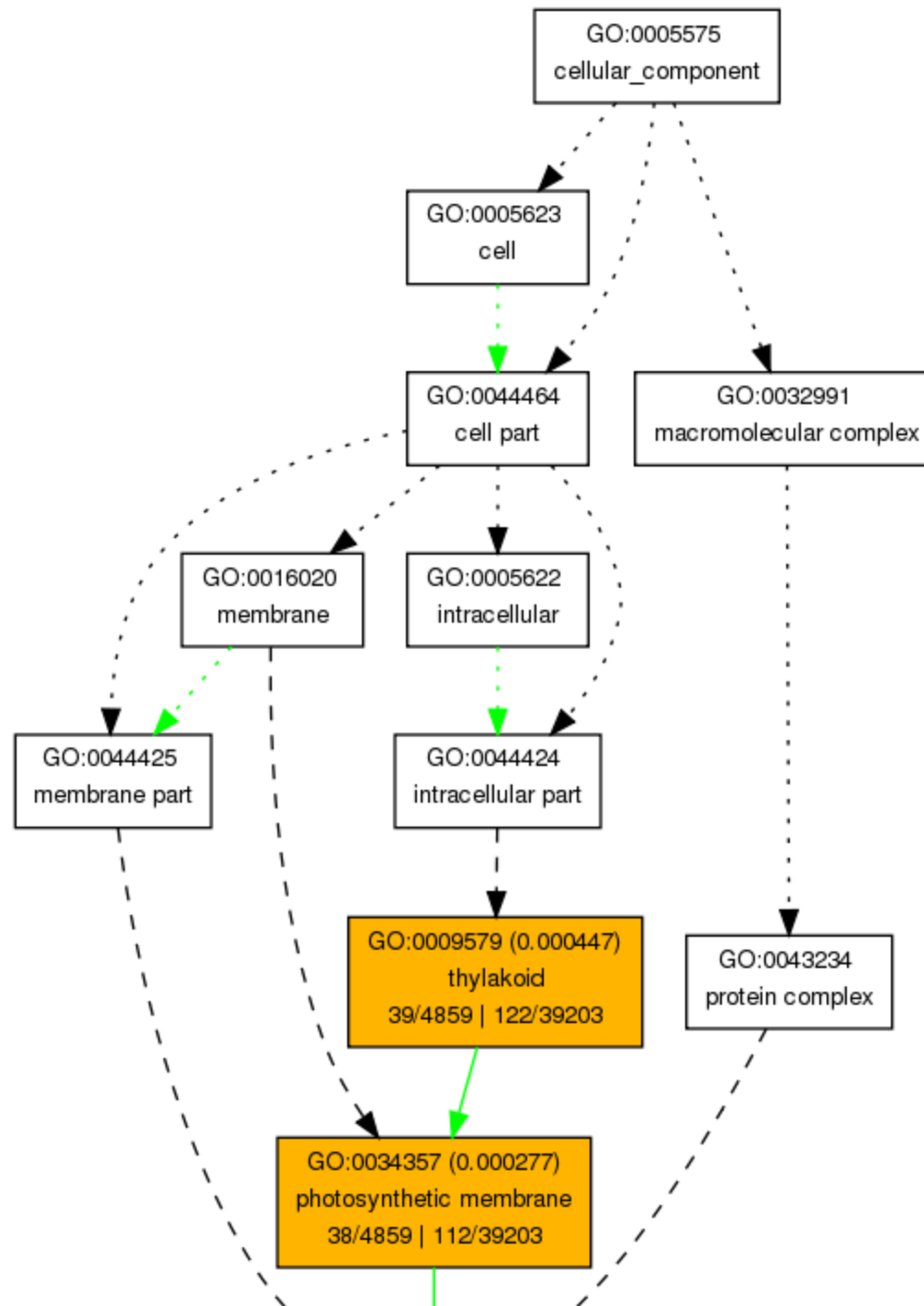

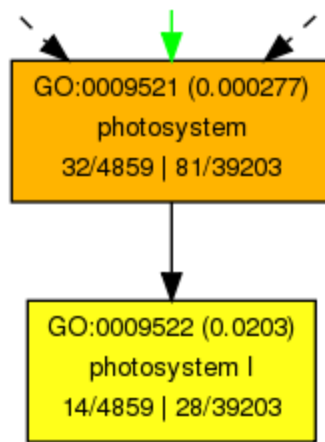

Supplement: Additional file 13: — GO enrichment analysis of maize lincRNAs functioning as miRNA targets associated with “cellular components”. (PDF 50 kb) [file 12864_2015_2024_MOESM13_ESM.pdf]

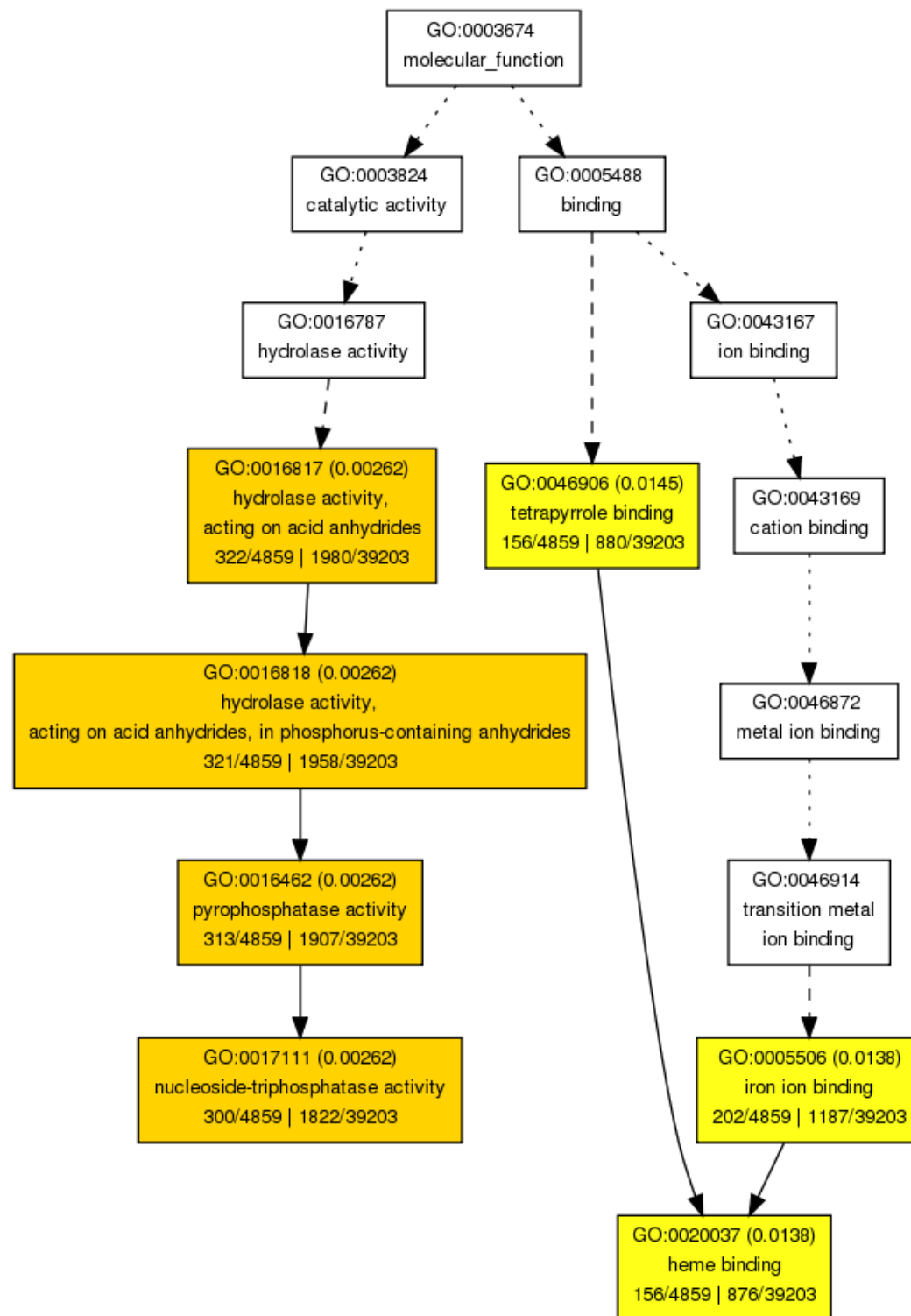

Supplement: Additional file 14: — GO enrichment analysis of maize lincRNAs functioning as miRNA targets associated with “molecular functions”. (PDF 65 kb) [file 12864_2015_2024_MOESM14_ESM.pdf]

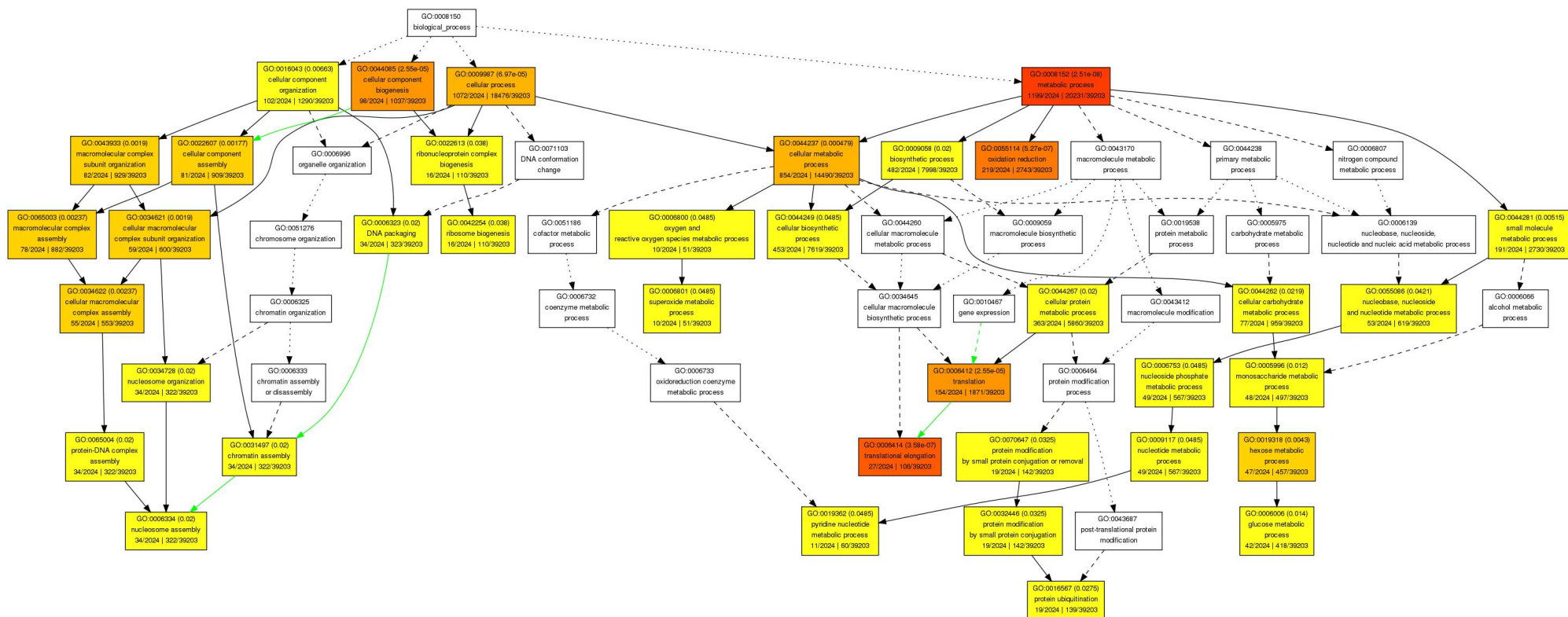

Supplement: Additional file 16: — GO enrichment analysis of maize lincRNAs functioning as miRNA decoys associated with “biological processes”. (PDF 1350 kb) [file 12864_2015_2024_MOESM16_ESM.pdf]

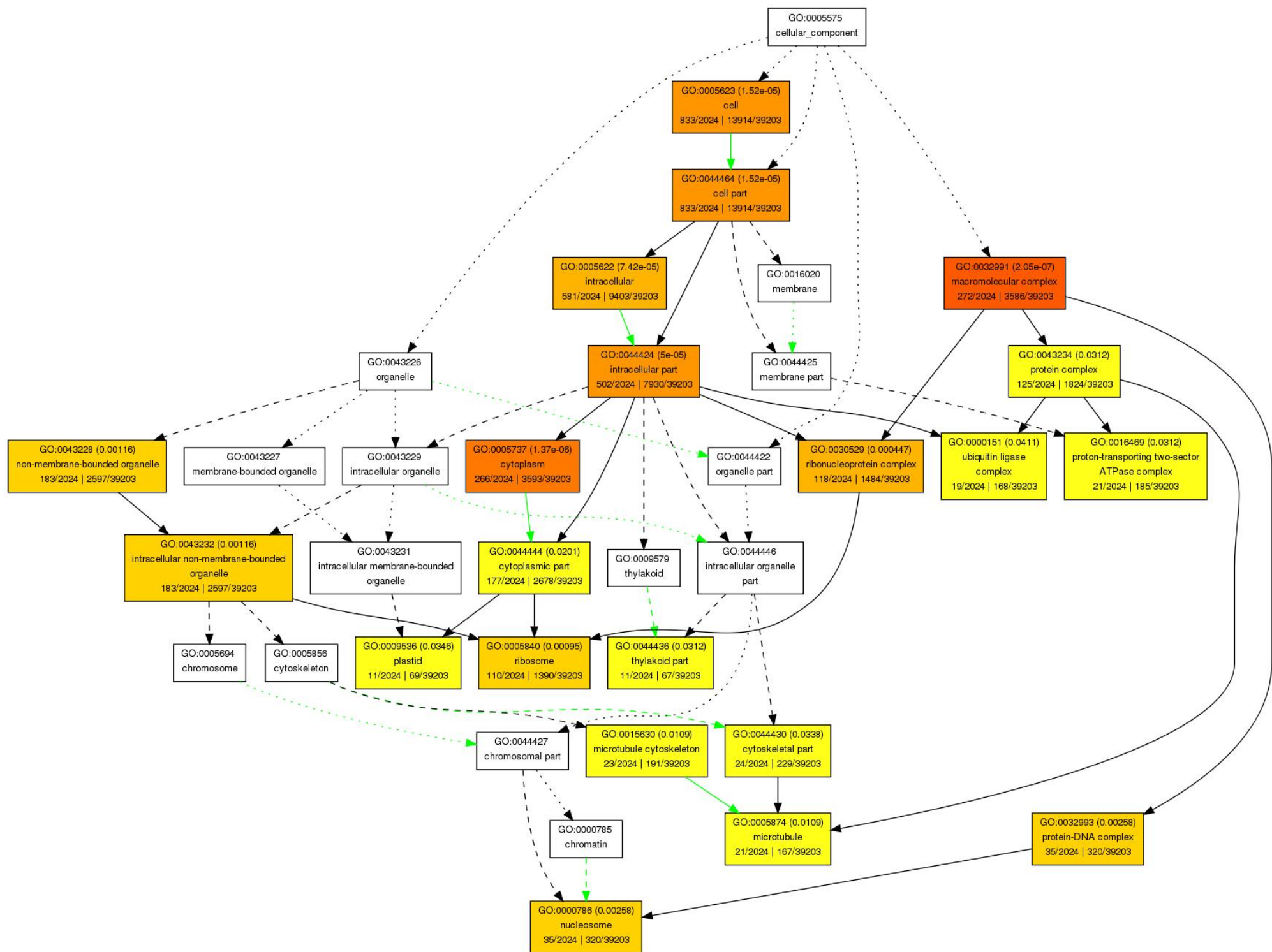

Supplement: Additional file 17: — GO enrichment analysis of maize lincRNAs functioning as miRNA decoys associated with “cellular components”. (PDF 773 kb) [file 12864_2015_2024_MOESM17_ESM.pdf]

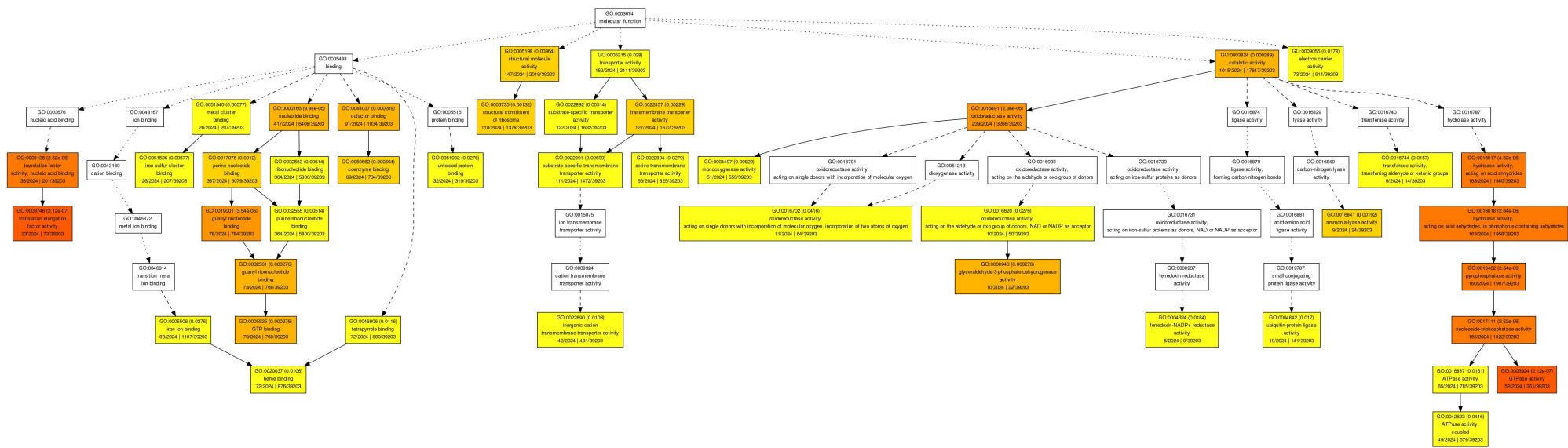

Supplement: Additional file 18: — GO enrichment analysis of maize lincRNAs functioning as miRNA decoys associated with “molecular functions”. (PDF 1498 kb) [file 12864_2015_2024_MOESM18_ESM.pdf]
