# Supplementary material for: Genome-wide identification and functional analysis of lincRNAs acting as miRNA targets or decoys in maize
Source: BMC Genomics. 2015 Oct 15;16:793. doi: 10.1186/s12864-015-2024-0 (PMC4608266; doi:10.1186/s12864-015-2024-0)
Supplement: Additional file 7: — The sequence logos of the 10 conserved lincRNA as miRNA decoys. (ZIP 1503 kb) [file 12864_2015_2024_MOESM7_ESM.zip › Additional file 7/eTM-159e-3p.pdf]

```
Boerner_Z27kG1_01522: 5' UGGAGCUUCGC-CAAGCCGAG 3'
                         |||||||o|   |||o||o|
    zma-miR159e-3p: 3' ACCUCGAGGGAAGUUUGGUUA 5'

Boerner_Z27kG1_22626: 5' GCGAGCUCCUG-CAGACCGAG 3'
                           |||||||o  ||o|||o|
    zma-miR159e-3p: 3' ACCUCGAGGGAAGUUUGGUUA 5'

  Li_TCONS_00012087: 5' AGGGGCUCUGUUGGAGCCAGC 3'
                          ||o||||o || o|o|||o
    zma-miR159e-3p: 3' ACCUCGAGGGAAGUUUGGUUA 5'

zhang_TCONS_00056321: 5' UGGAGCUCCCAGAAGGCCAGA 3'
                         ||||||||||   |oo|||o
    zma-miR159e-3p: 3' ACCUCGAGGGAAGUUUGGUUA 5'
```

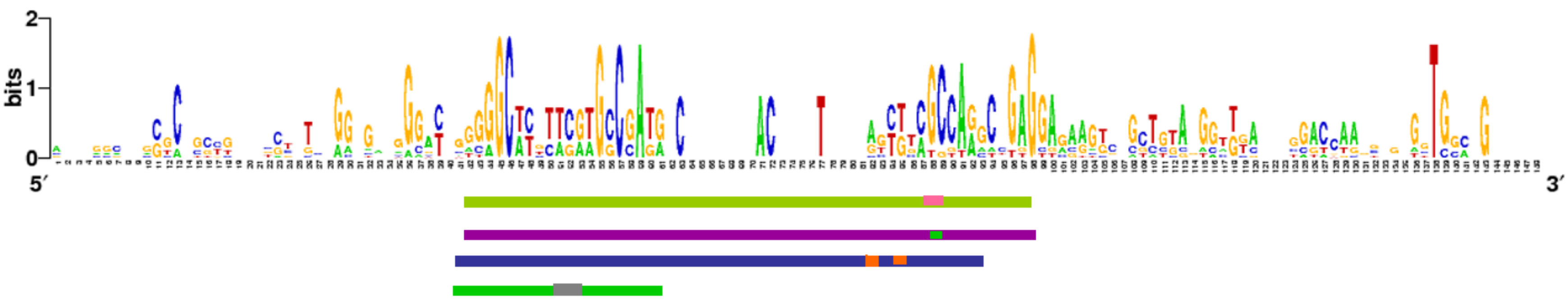


```
                                                                                                                          *
1. zma-eTMmiR159e-3p_1    ACGCCGCGTGCGCCGCCTTGCCGCCCGCGGAGAAGAGGCGGTGG-------------------------------------AGCTTCGCCAAGCCGAGGAACAGCGTGGCTGACGGCGGCGCCCGGCGGCCCTCCGT-----------
2. zma-eTMmiR159e-3p_2    AACTGCCACGCGCCGGTGTCTCACTGTTGATTCTCGGCCTCGCG-------------------------------------AGCTCCTGCAGACCGAGCGGAGCGGCATTGGACGGCGGAGCGCAAACTAGCGGTTT-----------
3. zma-eTMmiR159e-3p_3    CTGGGGAGCCCTAGTCCCGTATGTGTAAGGGGAAGGCGCCAGGGGCTCT--------------------------------GTTGGAGCCAGCCTGAGTACAAGTTACCCTCATGAAGCACCAGCATATGAT----------------
4. zma-eTMmiR159e-3p_4    AATCAAGAAAGGCTGGGGCAGCCCATCTGCGCTCAGCTCATGGAGCTCCCAGAAGGCCAGAACGCTTCCTACTGGCT----ACTGCAGCTAGACAGGGGTCAGG--------------------------------------------
5. bdi-eTMmiR159e-3p      GGTTGGCTTGGGCACTTGC----------GGAGGTGGACGGTTGGCTCT--------------------------------GTTGGAGCCAGGAGGTGGAGGTGGCGGGTGGCGTCTGGGAGCTCTCCAGCTCTAGCTCCCC------
6. pvi-eTMmiR159e-3p_1    -------------------CGGCTCTCCAGCGAGGGGATTGGGGGCATGTTCGTGCCGATG--------------------AGCTTCGCCAAGCCGAGGAGAAGTCAGCTGTAGGGTTTATTTGGACCAAGGAGGGGTG---------
7. pvi-eTMmiR159e-3p_2    -------------------CGGCTCTCCGGCGAGGGGATTGGGGGCATGTTCGTGCCGATG--------------------AGCTTCGCCAAGCCGAGGAGAAGTCAGCTGTAGGGTTTATTTGGACCAAGGAGGGGTG---------
8. pvi-eTMmiR159e-3p_3    ----------------------------GGCGAGGGGATTGGGGGCATGTTCGTGCCGATG--------------------AGCTTCGCCAAGCCGAGGAGAAGTCAGCTGTAGGGTTTATTTGGACCAAGGAGGGGTGGATGTAGGT
9. pvi-eTMmiR159e-3p_4    -----------TCCCCCGATATGGGGTGGGGGGAGGGATAGAGGGCTCT--------------------------------GTTGGAGCCAGCCTTAGGAAGGAGAGGTTCTGAACATGCGAGTGGTTAATCCAAACTGGCAG-----
10. pvi-eTMmiR159e-3p_5   -------------------CGGCTCTCCGGCGAGGGGATTGGGGGCATGTTCGTGCCGATG--------------------AGCTTCGCCAAGCCGAGGAGAAGTCAGCTGTAGGGTTTATTTGGACCAAGGAGGGGTG---------
11. sbi-eTMmiR159e-3p     -----------CCAGCTTCACTCGGTCAAAAGTCAGAACACACAGCTCCCAGAAGGCCAGAGCAAGCATCACACAGTATTCCCTGTCGCGTGGTTGAGGAACATGCTCCAC-------------------------------------
```
