# Supplementary material for: Genome-wide identification and functional analysis of lincRNAs acting as miRNA targets or decoys in maize
Source: BMC Genomics. 2015 Oct 15;16:793. doi: 10.1186/s12864-015-2024-0 (PMC4608266; doi:10.1186/s12864-015-2024-0)
Supplement: Additional file 7: — The sequence logos of the 10 conserved lincRNA as miRNA decoys. (ZIP 1503 kb) [file 12864_2015_2024_MOESM7_ESM.zip › Additional file 7/eTM-167f-3p.pdf]

```
Boerner_Z27kG1_02792: 5' CCGGCGACUGCGCGGCGGGCACGAUC 3'
                             | o|||||||    o||||||||
      zma-miR167f-3p: 3' CCACUUUGACGCG----UCGUGCUAG 5'
```

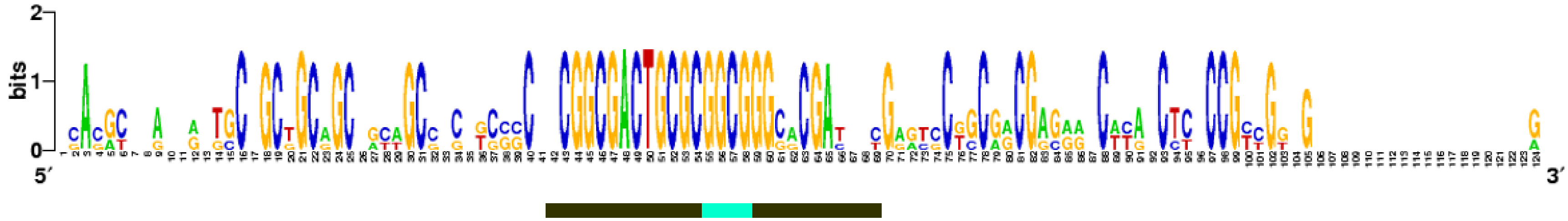


```
                                 *    *                  *  ******************              *  *  **     *    *   ***
zma-eTMmiR167f-3p  CGACGCCGAGCACTGCA---GCAGCCGTAGCCCCCTCGGCGCCGGCGACTGCGCGGCGGGCACGAT--CGAGTCCTGCGACGAGAAGCTCAGCTCGCCGTTGGA-------------------G
bdi-eTMmiR167f-3p  TCAGATATGAGGTTCCCGCTG-----------ACGGCCCCATCGGCGACTGCGCGGCGGGC-------------CGGCGGCGAGGGACATATCCTTCCGTCGGGGATCCGATCCGAGGTTCCCG
sbi-eTMmiR167f-3p  ----GCGGAGCACTGCAGCTGCAGCTGCAGCCGCAGCCCCCTCGGCGACTGCGCGGCGGGCACGAT--CGAGTCCTGCGACGAGAAGCTCAGCTCGCCGCCGTTG------------------G
sit-eTMmiR167f-3p  -CACGCGCACTGGGGCGGCGGCGGCGACTGCGCGGTGGGCGGCGGCGACTGCGCGGCGGGGGCGACAGTGGACGCGCCAGCGGCGGCCATGACTCCCCGC-----------------------A
```
