# Supplementary material for: Genome-wide identification and functional analysis of lincRNAs acting as miRNA targets or decoys in maize
Source: BMC Genomics. 2015 Oct 15;16:793. doi: 10.1186/s12864-015-2024-0 (PMC4608266; doi:10.1186/s12864-015-2024-0)
Supplement: Additional file 7: — The sequence logos of the 10 conserved lincRNA as miRNA decoys. (ZIP 1503 kb) [file 12864_2015_2024_MOESM7_ESM.zip › Additional file 7/eTM-169q-3p.pdf]

```
Boerner_Z27kG1_03458: 5' CUCGGCCC--GGGCCUGCC 3'
                         || o|||   o||||||||
       zma-miR169q-3p: 3' GAAUCGGUCUUCCGGACGG 5'

   Li_TCONS_00041379: 5' CGCCGCCGC-GGGCCUGCC 3'
                         |   |||o  o||||||||
       zma-miR169q-3p: 3' GAAUCGGUCUUCCGGACGG 5'

   Li_TCONS_00096947: 5' CGCAGCCACCACCGAAGGCCUGUC 3'
                         |  |||||     |||||||||o|
       zma-miR169q-3p: 3' GAAUCGGU-----CUUCCGGACGG 5'

   Li_TCONS_00064018: 5' CGCAGCCACCACCGAAGGCCUGUC 3' (104-127)
                         |  |||||     |||||||||o|
       zma-miR169q-3p: 3' GAAUCGGU-----CUUCCGGACGG 5'

   Li_TCONS_00064018: 5' CGCAGCCACCACCGAAGGCCUGUC 3' (129-152)
                         |  |||||     |||||||||o|
       zma-miR169q-3p: 3' GAAUCGGU-----CUUCCGGACGG 5'

zhang_TCONS_00028666: 5' ACCAGCCGGCCGGCCUGCU 3'
                            ||||o|  |||||||o
       zma-miR169q-3p: 3' GAAUCGGUCUUCCGGACGG 5'

zhang_TCONS_00056448: 5' CACAACCGGACACCGAGGCCUGCC 3'
                         |  | ||o||     |||||||||
       zma-miR169q-3p: 3' GAAUCGGUCU-----UCCGGACGG 5'
```

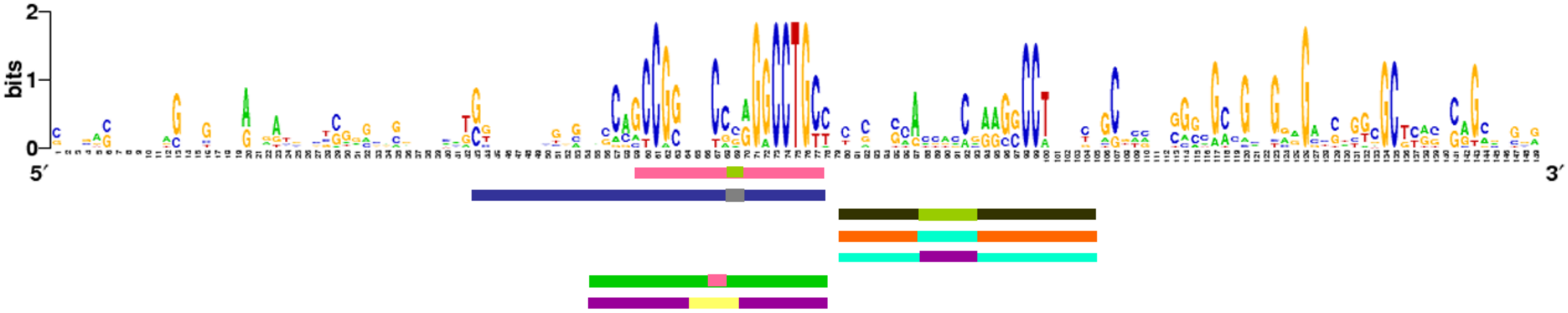


```
1. zma-eTMmiR169q-3p_1     GTGCA-----------CGCGGGACCCGCCGCCGCGGCCATGTCG-----TTCGGCTGGCTCGG---CCCGGGCCTGCCGT----------------------TCGGCGCCCGGGACGCCGCTGCGGAGGCGGA--------GGAGCCCG
2. zma-eTMmiR169q-3p_2     TGCCGCGCTCTAGACTACCGGAATTGCTCGCCAGGGCCTCTACG---------------CCGC---CGCGGGCCTGCCCTCCTTTCGTGGACCGGCCCCATCACGACGCCTTCGCC-------------------------------GG
3. zma-eTMmiR169q-3p_3     CCGGA--------------------------------GGCCTGT-----CGTGCAGCCGCCGC---TAGAGACCTGTCGCGCAGCCACCACCGAAGGCCT--GTCGCGCA---GCCACCACCGAAGGCCTGTC----GCGCAGCCGCCG
4. zma-eTMmiR169q-3p_4     CTAGA--------------------------------GACCTGT-----CGCGCAGCCACCAC---CGAAGGCCTGTCGCGCAGCCACCACCGAAGGCCT--GTCGCGCAGCCGCCGCCGCCGGAGACCTGTC----GCACAGC---AG
5. zma-eTMmiR169q-3p_5     CCGGA--------------------------------GGCCTGT-----CGTGCAGCCGCCGC---TAGAGACCTGTCGCGCAGCCACCACCGAAGGCCT--GTCGCGCAGCCGCCGCCGCCGGAGACCTGTC----GCGCAGC---AG
6. zma-eTMmiR169q-3p_6     CCAGT--------CGGTCTACAGACAGTGAGGGGATCGATATCT-----GCTAAACCAGCCGG---CC--GGCCTGCTTGTCCTCTAATTGAAGGGCCCTAGCTTAGCTAGATAGCAC-------------------------------
7. zma-eTMmiR169q-3p_7     CGCGA--------------GGTTTGTCGGAGGCGCGTGACTTGT-----CGAAGCACAACCGGACACCGAGGCCTGCC----------------------------------GGCGGTAGTGGCTGCACAGGGGCTCAGTCCTGGAGGA
8. bdi-eTMmiR169q-3p_1     GCCTAGAGGGGAGAGGAAGAAGATGGCCCGGGCAGAGAGAAGGG-----CGGGGGCCAGCCGG---CC--GGCCTGCTGCAGGAG-----------------AGAGCTAGAGGGGAGAGGAAGAA----------------------GA
9. bdi-eTMmiR169q-3p_2     ------------------GAAGATGGCCGGGGCGGAGAGAAGGG-----CGGGGGCCAGCCGG---CC--GGCCTGCTGCGGGAG-----------------AGAGCTAGAGGGGAGAGGAAGAAGATGAGGC----AGAGAGAAGGGT
10. bdi-eTMmiR169q-3p_3    CACCACACTCATGCTGCTGGTAATATTTCTGATACGGGTAAGCG------TCATGGCAGCCGG---CC--GGCCTGCTTACACCGA----------------ACGGCATCCCCAGTGCTGCAGGA----------------------GA
11. bdi-eTMmiR169q-3p_4    ------------------GAAGATGGCCGGGGCAGAGAGAAGGG-----AGGGGGCCAGCCGG---CC--GGCCTGCTACGGGTC-----------------TGAGCTAGAGGGGAGAAGAAGAAGATGAGGC----AGAGAGAAGGGC
12. bdi-eTMmiR169q-3p_5    GACAG----GCAGTTGGCAATAATAATTGGAAATGCTTTCTTGCGGACACGGCAACCAGCCGG---CC--GGCCTG----------------------------------------GATCATGGGGGTGGCGTGCGCTTCCGGCCTTTG
13. bdi-eTMmiR169q-3p_6    CGTCAGCACTCGCTCCAATAAGTCCTAGCTAGCAAGATCCATCC-----ATCCAGCCAGCCGG---CC--GGCCTGCCACTCGG------------------TCCGGATGGAGGGCGCCGGCGAGG---------------------AG
14. pvi-eTMmiR169q-3p      CGAGT-----------ACCATCGCCCCTCCTCCTCAACTCCGGC-----GAGCCCGACGCCGC---CGCGGGCCTGCCCTTCCAGA----------------CCTCCTCCGCCCGCGCCGACTCCGCCTTTAG--------------CA
15. sbi-eTMmiR169q-3p_1    GCCGC-----------GGGATGAGGCGGCGGAGGCGGTTTCTGT-----GGCCATGAGCTCGG---CCCGGGCCTGCCATCATCG-----------------CCGGCGACCGGCGAGCCGCTGGGGCTCTCGC----A---------CA
16. sbi-eTMmiR169q-3p_2    AAGAG-----------------GAGAGGAGAGGAGCCCAGCACG-----TCGGGACCAGCCGG---CC--GGCCTGCATC----------------------TCGTCGTCGTCGATGACGGCCGGGACCATGCGCTTGCTGGGCGGGGA
```
