# Supplementary material for: Genome-wide identification and functional analysis of lincRNAs acting as miRNA targets or decoys in maize
Source: BMC Genomics. 2015 Oct 15;16:793. doi: 10.1186/s12864-015-2024-0 (PMC4608266; doi:10.1186/s12864-015-2024-0)

```
Boerner_Z27kG1_04122: 5' AGGGGAGCCACUCCGGCGUCG 3'
                          o  ||||||  ||oo|o|||
     zma-miR171f-5p: 3' CUAACUCGGUACGGUUGUAGC 5'

   Li_TCONS_00027786: 5' CGUUGAGCCUCGUCGACAUCC 3'
                          o|||||||  |o|o|||||
     zma-miR171f-5p: 3' CUAACUCGGUACGGUUGUAGC 5'

   Li_TCONS_00096642: 5' GGUUGGGCUC-GUCGGCGUCG 3'
                         |o|||o||o  |o|oo|o|||
     zma-miR171f-5p: 3' CUAACUCGGUACGGUUGUAGC 5'
```

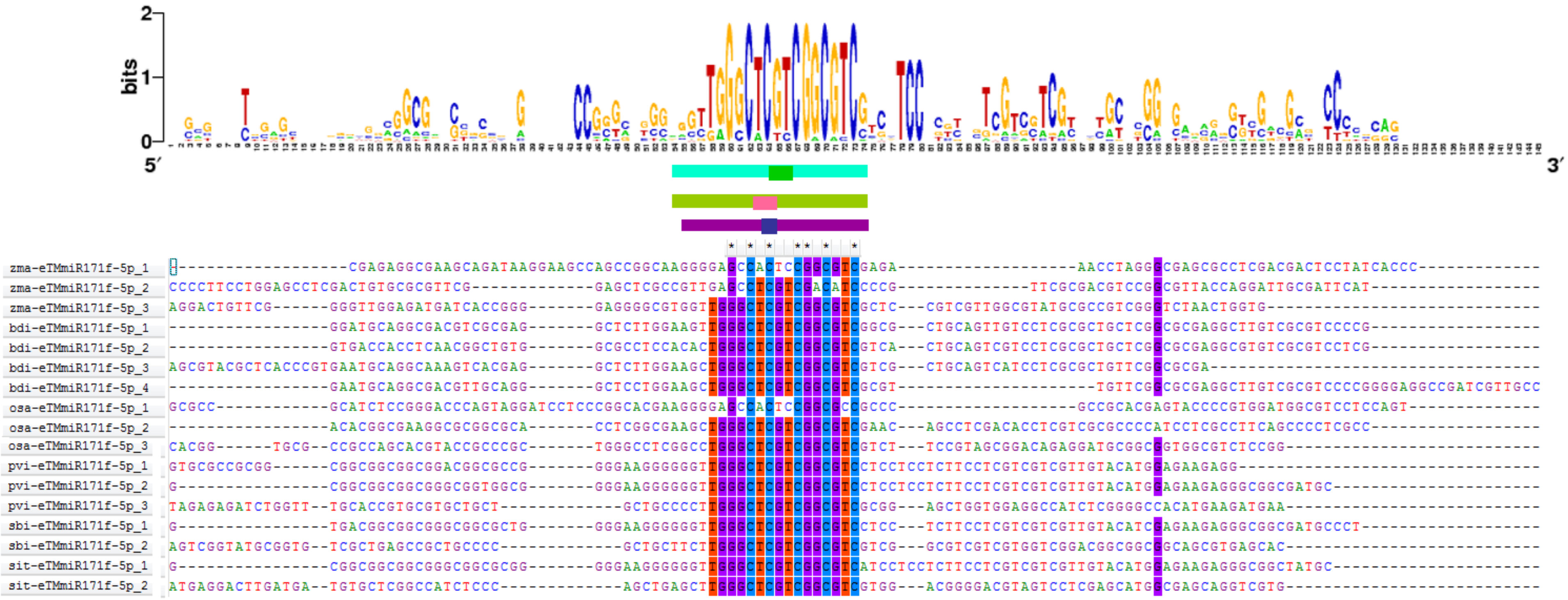

Supplement: Additional file 7: — The sequence logos of the 10 conserved lincRNA as miRNA decoys. (ZIP 1503 kb) [file 12864_2015_2024_MOESM7_ESM.zip › Additional file 7/eTM-171f-5p.pdf]
