# Supplementary material for: Genome-wide identification and functional analysis of lincRNAs acting as miRNA targets or decoys in maize
Source: BMC Genomics. 2015 Oct 15;16:793. doi: 10.1186/s12864-015-2024-0 (PMC4608266; doi:10.1186/s12864-015-2024-0)
Supplement: Additional file 7: — The sequence logos of the 10 conserved lincRNA as miRNA decoys. (ZIP 1503 kb) [file 12864_2015_2024_MOESM7_ESM.zip › Additional file 7/eTM-394b-3p_394a-3p.pdf]

```
Boerner_Z27kG1_10860: 5' UCAUGGCGGAGGAGUGCCCGCCC 3'
                         o  ||||o|    o|||||o||
    zma-miR394a/b-3p: 3' GUAACCGUCA---UACGGGUGGA 5'

   Li_TCONS_00072326: 5' CAUUGGGUUU-UGCCCACCG 3'
                         ||||||   | ||||||||
    zma-miR394a/b-3p: 3' GUAACCGUCAUACGGGUGGA 5'

   Li_TCONS_00089213: 5' GUGUGGCGGUAACUGCUCACCA 3'
                            ||||o|||  |||o||||
    zma-miR394a/b-3p: 3' GUAACCGUCAU--ACGGGUGGA 5'
```

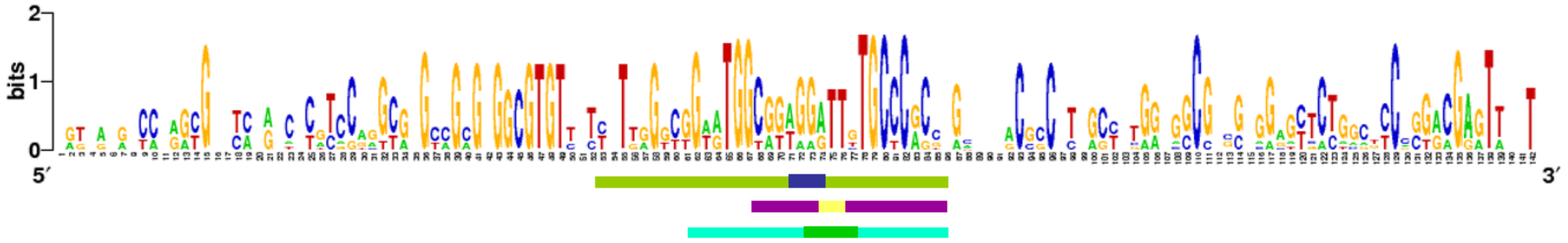


```
1. zma-eTMmiR394a/b-3p_1   -------------CGGTCCCGGTCCGTCCGAGCAAGCAGAGCGGCGTGTCGTTC---------ATGGCGGAGGA--GTGCCCGCCCAC---------------TGGCGGCGGCGTGGAGCTCTGGCTTCCGGACGAGTT---
2. zma-eTMmiR394a/b-3p_2   AGGTAGACTCGGGTGAGTACATCTTACCGAGGT--------------------------TTGTGTGGCATTGGGTTTTGCCCACCG----------TTTGGTGCGGTACCGCGGCGGAGCCCCAACACCGGTGAGA------
3. zma-eTMmiR394a/b-3p_3   -------GCAAGGCGTCCATGTCCTATCCAGGCGGG--------------ATTATTGGTCGGTGTGGCGGTAA---CTGCTCACCAGAGCAACCCCATCACTATGAAGCCGCACGGAGTCGATG------------------
4. bdi-eTMmiR394a/b-3p_1   CGTGGCGGCCGAGCGCCTCGAGCGCGCGCGCGC--------------------------------GGCGGAGGA--GTGCCCGCGGGAAGCGCGGCTCGGCCTGGAGCGCGAGGGAGCACTCCTCGGCCACGGC--------
5. bdi-eTMmiR394a/b-3p_2   -ATCAAGTCCCAATGGATCTACCACTTCCAGTT----------------------------------TATTGGGTTTTGCCCACCTGTCAGACGCCGTCGCCGTAGCGGCCGGCAGGGGTTCTGGAGC-------GATATAT
6. pvi-eTMmiR394a/b-3p     ----------------------CCCGTCCAAGCGTGCCGCGGGGCGTGTTTTCCTTGGGCGGAATGGCGGAGGA--GTGCCCGCGCGC------------------CGGCGGCGCCGAGTTCTGGCTCCCGGACGAGTTCCT
7. sit-eTMmiR394a/b-3p     --------------------GCACCGTACTGGCGTGTCGCGAG--GTGTTTCCTTGAGGCGGAATGGCGGAGGA--GTGCCCGGTAGC---------------TGGCGGCGTCGCGGAGTTCTGGCTTCCCGACGAGTT---
```
