# Supplementary material for: Genome-wide identification and functional analysis of lincRNAs acting as miRNA targets or decoys in maize
Source: BMC Genomics. 2015 Oct 15;16:793. doi: 10.1186/s12864-015-2024-0 (PMC4608266; doi:10.1186/s12864-015-2024-0)
Supplement: Additional file 7: — The sequence logos of the 10 conserved lincRNA as miRNA decoys. (ZIP 1503 kb) [file 12864_2015_2024_MOESM7_ESM.zip › Additional file 7/eTM-528a-3p_zma-miR528b-3p.pdf]

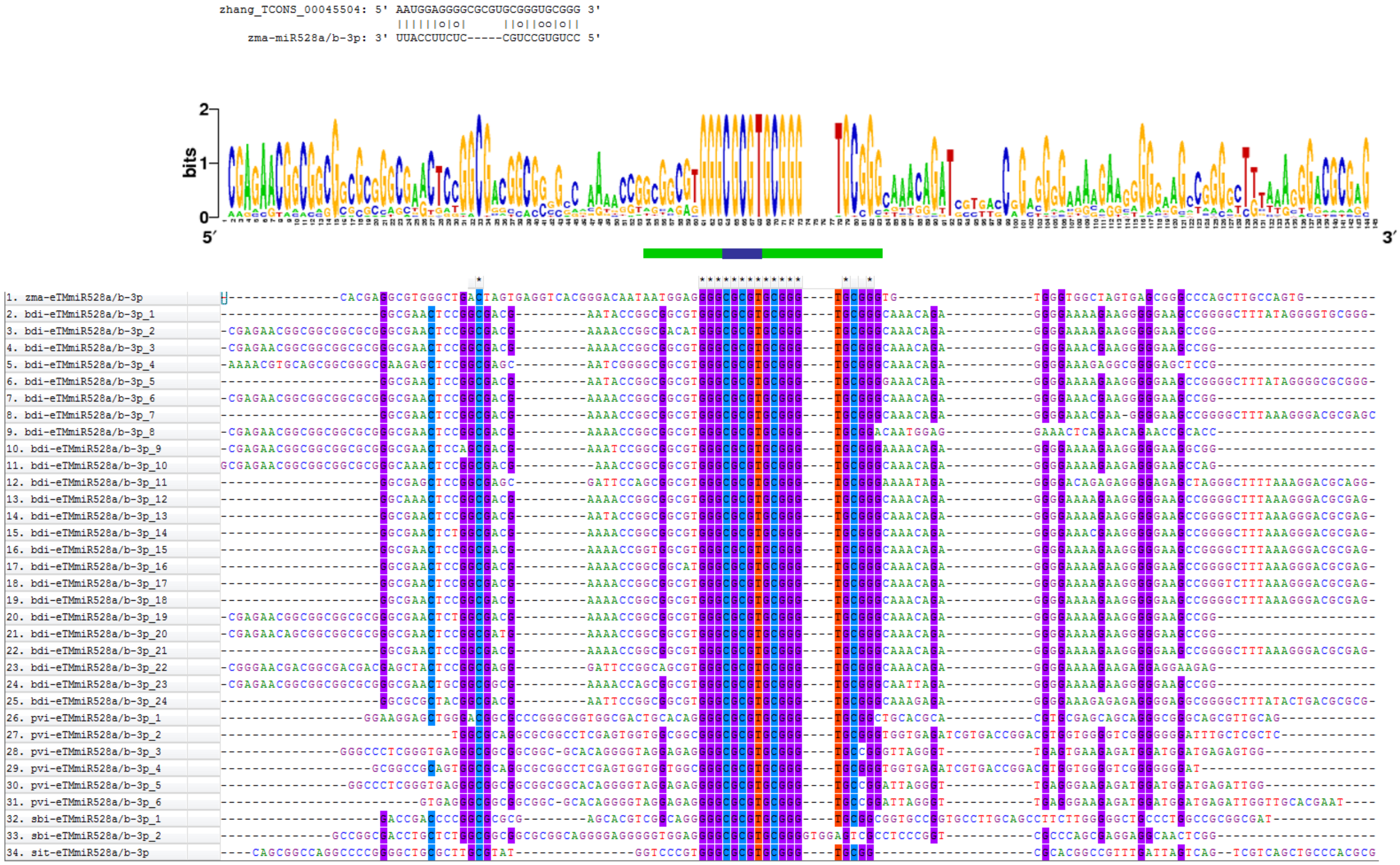
zhang_TCONS_00045504: 5' AAUGGAGGGGCGCGUGCGGGUGCGGG 3'
zma-miR528a/b-3p: 3' UUACCUUCUC-----CGUCCGUGUCC 5'
bits
2
1
0
5′
3′
1. zma-eTMmiR528a/b-3p
2. bdi-eTMmiR528a/b-3p_1
3. bdi-eTMmiR528a/b-3p_2
4. bdi-eTMmiR528a/b-3p_3
5. bdi-eTMmiR528a/b-3p_4
6. bdi-eTMmiR528a/b-3p_5
7. bdi-eTMmiR528a/b-3p_6
8. bdi-eTMmiR528a/b-3p_7
9. bdi-eTMmiR528a/b-3p_8
10. bdi-eTMmiR528a/b-3p_9
11. bdi-eTMmiR528a/b-3p_10
12. bdi-eTMmiR528a/b-3p_11
13. bdi-eTMmiR528a/b-3p_12
14. bdi-eTMmiR528a/b-3p_13
15. bdi-eTMmiR528a/b-3p_14
16. bdi-eTMmiR528a/b-3p_15
17. bdi-eTMmiR528a/b-3p_16
18. bdi-eTMmiR528a/b-3p_17
19. bdi-eTMmiR528a/b-3p_18
20. bdi-eTMmiR528a/b-3p_19
21. bdi-eTMmiR528a/b-3p_20
22. bdi-eTMmiR528a/b-3p_21
23. bdi-eTMmiR528a/b-3p_22
24. bdi-eTMmiR528a/b-3p_23
25. bdi-eTMmiR528a/b-3p_24
26. pvi-eTMmiR528a/b-3p_1
27. pvi-eTMmiR528a/b-3p_2
28. pvi-eTMmiR528a/b-3p_3
29. pvi-eTMmiR528a/b-3p_4
30. pvi-eTMmiR528a/b-3p_5
31. pvi-eTMmiR528a/b-3p_6
32. sbi-eTMmiR528a/b-3p_1
33. sbi-eTMmiR528a/b-3p_2
34. sit-eTMmiR528a/b-3p
