# Supplementary material for: Genome-wide identification and functional analysis of lincRNAs acting as miRNA targets or decoys in maize
Source: BMC Genomics. 2015 Oct 15;16:793. doi: 10.1186/s12864-015-2024-0 (PMC4608266; doi:10.1186/s12864-015-2024-0)

```
    Li_TCONS_00081252: 5' GAGGGGAGAGAGGGAGAGGGCUC 3'
                          ||| |||      |o||||o||||
   zma-miR319a/c-5p: 3' CUCACCUGA---CUUCUCUCGAG 5'

zhang_TCONS_00063429: 5' GGGUGGGUGAGGGGGAGAGGGCUC 3'
                         |o||||oo      |o||||o||||
   zma-miR319a/c-5p: 3' CUCACCUGA----CUUCUCUCGAG 5'
```

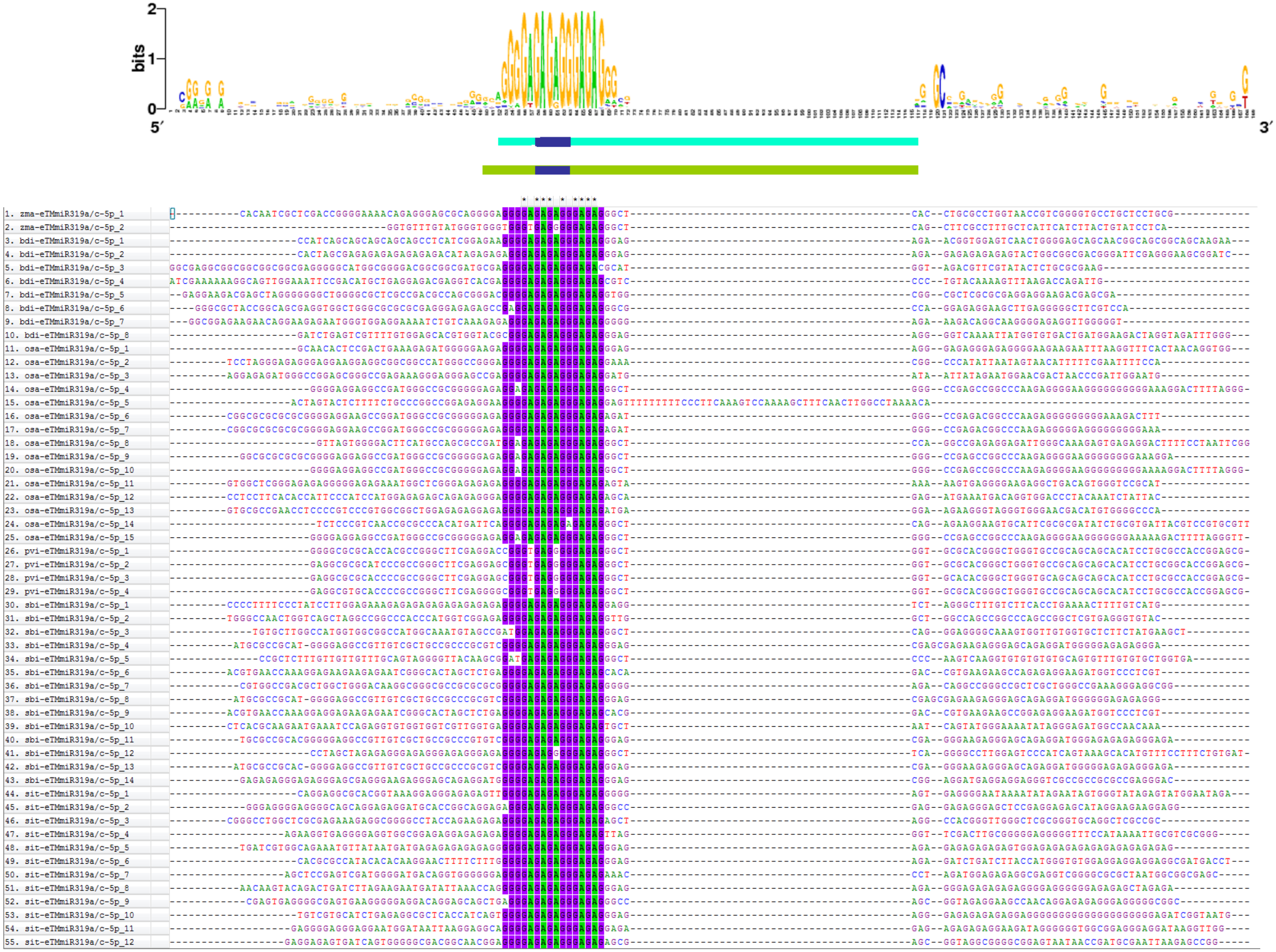

Supplement: Additional file 7: — The sequence logos of the 10 conserved lincRNA as miRNA decoys. (ZIP 1503 kb) [file eTM-319a-5p_319c-5p.pdf › Additional file 7/eTM-319a-5p_319c-5p.pdf]
